# Yoga or Strengthening Exercise for Knee Osteoarthritis: A Randomized Clinical Trial

**DOI:** 10.1001/jamanetworkopen.2025.3698

**Published:** 2025-04-08

**Authors:** Bedru J. Abafita, Ambrish Singh, Dawn Aitken, Changhai Ding, Steffany Moonaz, Andrew J. Palmer, Leigh Blizzard, Andrew Inglis, Stan J. J. Drummen, Graeme Jones, Kim L. Bennell, Benny Antony

**Affiliations:** 1 Menzies Institute for Medical Research, University of Tasmania, Hobart, Tasmania, Australia; 2 Clinical Research Centre, Zhujiang Hospital, Southern Medical University, Guangzhou, China; 3 Clinical Research Centre, Guangzhou First People’s Hospital, School of Medicine, South China University of Technology, Guangzhou, China; 4 Department of Clinical and Health Services Research, Southern California University of Health Sciences, Whittier; 5 Centre for Health, Exercise and Sports Medicine, Department of Physiotherapy, School of Health Sciences, University of Melbourne, Melbourne, Victoria, Australia

## Abstract

**Importance:**

There is limited evidence on the comparative effectiveness of different exercise modalities, such as yoga and strengthening exercises, for managing knee osteoarthritis (OA).

**Objective:**

To compare the effectiveness of yoga vs strengthening exercise for reducing knee pain over 12 weeks in patients with knee OA.

**Design, Setting, and Participants:**

This single-center, assessor-blinded (for nonpatient-reported outcomes), parallel-arm, active-controlled, superiority randomized clinical trial included adults aged 40 years or older with knee OA and knee pain levels of 40 or higher on a 100-mm visual analog scale (VAS) in Southern Tasmania, Australia. Participants were recruited from April 2021 to June 2022, and follow-up was completed in December 2022. Data were analyzed from May 2023 to July 2024.

**Interventions:**

Participants were randomized 1:1 to the yoga and strengthening exercise groups. Both groups attended 2 supervised and 1 home-based session per week for 12 weeks followed by 3 unsupervised home-based sessions per week for weeks 13 to 24.

**Main Outcomes and Measures:**

The primary outcome was the between-group difference in VAS score over 12 weeks assessed using a range of 0 (no pain) to 100 (worst possible pain) with a prespecified noninferiority margin of 10 mm. Secondary outcomes included knee pain over 24 weeks; Western Ontario and McMaster Universities Osteoarthritis Index (WOMAC) knee pain, function, and stiffness; patient global assessment; Osteoarthritis Research Society International–Outcome Measures in Rheumatology Clinical Trials response; physical performance measures; leg muscle strength; health-related quality of life assessed via the Assessment of Quality of Life–8 Dimensions (AQol-8D) utility score; depression assessed with the Patient Health Questionnaire–9; and neuropathic pain assessment over 12 and 24 weeks. Analyses were based on the intention-to-treat principle.

**Results:**

In total, 117 participants were randomized to the yoga (n = 58) or strengthening exercise (n = 59) program. Baseline characteristics of the participants were similar, with a mean (SD) age of 62.5 (8.3) years, and 85 participants (72.6%) were female. The mean (SD) baseline VAS knee pain score of 53.8 (16.0) indicated moderate knee pain. Over 12 weeks, the between-group mean difference in VAS knee pain change was −1.1 mm (95% CI, −7.8 to 5.7 mm), which was not statistically significant but remained within the prespecified noninferiority margin. Of 27 secondary outcomes assessed over 12 and 24 weeks, 7 were statistically significant in favor of yoga. The yoga group showed modestly greater improvements than the strengthening exercise group (between-group differences) over 24 weeks for WOMAC pain (−44.5 mm [95% CI, −70.7 to −18.3 mm]), WOMAC function (−139 mm [95% CI, −228.3 to −49.7 mm]), WOMAC stiffness (−17.6 mm [95% CI, −30.9 to −4.3 mm]), patient global assessment (−7.6 mm [95% CI, −15.1 to −0.2 mm]), and 40-m fast-paced walk test (1.8 [95% CI, 0.4-3.2]). In addition, the yoga group had a modestly greater improvement than the strengthening exercise for depression at 12 weeks (between-group difference in PHQ-9 score, −1.1 [95% CI, −1.9 to −0.2]) and quality of life at 24 weeks (between-group difference in AQoL-8D score, 0.04 [95% CI, 0.0 to 0.07]). Adverse events were similar in both groups and mild.

**Conclusion and relevance:**

In this randomized clinical trial, yoga did not significantly reduce knee pain compared with strengthening exercises. However, yoga was found to be noninferior to strengthening exercises, suggesting that integrating yoga as an alternative or complementary exercise option in clinical practice may help in managing knee OA.

**Trial registration:**

ANZCTR.org Identifier: ACTRN12621000066886.

## Key Points

**Question:**

Does yoga lead to a significant reduction in knee pain compared with strengthening exercises over 12 weeks in patients with knee osteoarthritis?

**Findings:**

In this randomized clinical trial of 117 participants, both yoga and strengthening exercises improved knee pain over 12 weeks, with no statistically significant between-group difference. Yoga was noninferior to strengthening exercises and showed modest improvements in knee symptoms, quality of life, and depression.

**Meaning:**

These findings suggest that integrating yoga as an alternative or complementary exercise option in clinical practice may help in managing knee osteoarthritis.

## Teaser

This randomized clinical trial compares the effectiveness of yoga vs a strengthening exercise program for reducing knee pain among adult patients with knee osteoarthritis.

## Introduction

Osteoarthritis (OA) is a prevalent musculoskeletal disorder that affects more than 595 million people worldwide,^1^ including about 51.8 million adults in the US and 3.2 million people in Australia.^2^ Knee OA is characterized by joint pain and dysfunction and is associated with impaired quality of life (QOL). Exercise therapy is recommended by all international guidelines as a first-line treatment for knee OA to improve pain and function.^3^ Both aerobic and strengthening exercises are effective, but there is limited evidence on the comparative effectiveness of different exercise modalities, such as yoga and strengthening exercises.^4^ To date, there is also uncertainty about the effect that mind-body exercise, such as yoga, has on knee OA.^4^

Yoga and strengthening exercises are 2 distinct interventions with potentially different mechanisms for relieving pain and improving physical function in knee OA. Strengthening exercises are primarily designed to improve muscle strength around the knee joint, enhancing joint stability and reducing pain caused by mechanical stress.^5^ In contrast, yoga incorporates physical postures, mindfulness, and breathing techniques, which may reduce pain through a combination of improved flexibility, stress reduction, and mind-body awareness.^6,7^

A systematic review pointed to the potential efficacy of yoga in improving pain, function, and stiffness in knee OA.^8^ However, the study highlighted the lack of methodologic quality and potential sources of bias within the original studies. A comprehensive network meta-analysis aimed at evaluating nonpharmacological interventions for pain relief in patients with OA highlighted that yoga was effective, especially among female participants, and yoga programs had high adherence.^9^

Many clinical guidelines recommend yoga for knee OA^4,10,11^; however, these recommendations remain conditional due to very low quality of evidence,^4,11^ often limited by small sample sizes,^12,13,14,15^ lack of prospective trial registration,^12,13,16^ high risk of bias,^12,13,14,16,17^ nonstandardized diagnostic criteria,^12,17^ and short follow-up durations.^12,13,15,16,17^ Most of the clinical trials compared yoga with usual care, while only a small number of trials directly compared yoga with another active intervention.^8,12,14^ Multiple international clinical practice guidelines recommend strengthening exercise for the management of knee and hip OA^4,10,11,18^; however, to our knowledge, no studies have directly compared yoga with an evidence-based strengthening exercise program.

Considering the limited availability of high-quality, conclusive evidence supporting the effectiveness of yoga for OA and the uncertainty about the most effective type of exercise for knee OA, this study aimed to assess the comparative effectiveness of yoga vs strengthening exercise over 24 weeks in patients with knee OA. We hypothesized that yoga would demonstrate superior efficacy compared with strengthening exercise in alleviating joint pain and improving functional limitations and QOL among patients with knee OA given the distinct mechanisms of yoga emphasizing mindfulness, flexibility, and holistic well-being, which are known to be independently associated with pain reduction.^6,7^ Additionally, establishing yoga as at least noninferior to strengthening exercise would support offering a patient-centered, holistic option that may enhance adherence and overall outcomes.

## Methods

### Trial Design and Oversight

The randomized comparative effectiveness trial of yoga and strengthening exercise for knee osteoarthritis (YOGA) study was a single-center, parallel-arm, assessor-blinded (for nonpatient-reported outcomes), active-controlled superiority, randomized clinical trial with a 1:1 allocation ratio and prespecified noninferiority margin. Participants were recruited from April 2021 to June 2022, and follow-up was completed in December 2022. The trial was registered at ACTRN12621000066886 prior to recruitment.^19^ The trial protocol was approved by the Tasmanian Health and Medical Human Research Ethics Committee. All participants gave written informed consent. Details of the trial protocol have been published^20^ and are provided in Supplement 1. This study report followed the 2013 Consolidated Standards of Reporting Trials Extension (CONSORT PRO Extension) for the reporting of patient-reported outcomes in randomized clinical trials.^21^

### Participants

We recruited 129 participants with knee OA from the Southern Tasmania region of Australia. We used various recruitment strategies, including collaboration with general practitioners, specialist rheumatologists, orthopedic surgeons, physiotherapists, and advertising through radio, TV, newspaper, social media, community notice boards, hospital notice boards, community newsletters, local press, physiotherapy practices, and exercise physiologists in the region. Participants were invited to the study center (Menzies Institute for Medical Research, Hobart, Australia) for a screening visit after the initial telephone screening. The visit comprised symptom assessment, medication history, safety for exercise clearance, and clinical assessment for American College of Rheumatology clinical criteria. The eMethods in Supplement 2 give a detailed list of eligibility criteria.

### Randomization and Blinding

Participants were randomly allocated to either the yoga program or the strengthening program with a 1:1 ratio allocation via computer-generated random numbers prepared by a statistician with no involvement in the trial. The administering institute maintained the code break for the full randomization schedule. Allocation concealment and blinding were maintained through the collaboration of 2 research nurses coordinating the trial. The clinical assessments were conducted by a research nurse who was unaware of the group allocation. Due to the nature of the intervention, participants were not blinded to the treatment allocation. Thus, patient-reported outcomes were not blinded.

### Interventions

A detailed description of the yoga and strengthening exercise protocol has been published^20^ and is included in the eAppendix in Supplement 2. Participants were randomly allocated to either a 24-week yoga program or strengthening program (each consisting of two 1-hour in-person group-based sessions and one 1-hour home-based session per week for the initial 12 weeks followed by 3 home-based sessions per week for another 12 weeks). Physiotherapists and yoga teachers led the group sessions during the first 12 weeks, with each trainer leading a group of 10 participants. Participants were instructed to continue their yoga or strengthening exercise routines at home for the subsequent 12 weeks. Weekly emails were sent through REDCap to encourage adherence to the home-based program, and leaflets and video instructions were provided to assist each participant with the home-based activities. The eAppendix in  Supplement 2 gives a detailed description of interventions.

### Outcomes Measures

We assessed key clinical outcomes focused on knee symptom severity drawn from previous clinical trials and recommendations by Osteoarthritis Research Society International (OARSI).^22^ Each participant was evaluated at baseline and at 4, 8, 12, 16, 20, and 24 weeks for visual analog scale (VAS) knee pain, Western Ontario and McMaster Universities Osteoarthritis Index (WOMAC), patient global assessment, and pain medication use. Additionally, assessments were conducted at baseline, 12 weeks, and 24 weeks for core physical function measures, leg muscle strength test, painDETECT, Patient Health Questionnaire–9 (PHQ-9), and assessment of QOL (Assessment of Quality of Life–8 Dimensions and EuroQol 5-Dimension 5-Level instruments) (eFigure 1 in Supplement 2).

### Primary Outcome

The primary outcome was a change in knee pain assessed by the VAS score (range, 0 to 100 mm, with higher numbers indicating greater pain) over 12 weeks. The minimal clinically important difference (MCID) for VAS knee pain between the groups was 15 mm.^23^

### Secondary Outcomes

Secondary outcomes^20^ were defined as the overall change from baseline to week 12 and the change from baseline to week 24 separately. Secondary outcomes included change in knee pain (assessed using the 100-mm VAS score) over 24 weeks, WOMAC pain score (range, 0-500 mm), WOMAC function (range, 0-1700 mm), WOMAC stiffness (range, 0-200 mm), and change in patient global assessment (range, 0-100 mm). An OARSI-recommended set of physical performance measures was also a secondary outcome,^24^ including a 30-second chair stand test (MCID, 2 stands),^25^ 40-m fast-paced walk test (MCID, 0.2 m/s),^25^ and stair climb test (the MCID has not been established), and health-related QOL assessed by the Assessment of Quality of Life–8 Dimensions (AQoL-8D; MCID score, 0.06)^26^ and EuroQol 5-Dimension 5-Level^27^ instruments scored from −0.04 to 1.00, with higher scores indicating better QOL^28^ and 1.00 indicating full health. Other secondary outcomes included depression assessed using the PHQ-9 score,^29^ neuropathic pain assessed by painDETECT (scores ranged from −1 to 38, with <12 indicating negative symptoms of neuropathic pain and ≥12, possible neuropathic pain),^30^ leg muscle strength assessed by dynamometry at the lower limb (involving both legs simultaneously), pain medication use, OARSI–Outcome Measures in Rheumatology Clinical Trials responder criteria to assess the treatment response,^31^ adverse events (AEs), and adherence to the intervention. We also estimated the noninferiority of VAS knee pain over 12 weeks based on a predefined noninferiority margin of 10 mm.^32^ Knee pain change from baseline to 24 weeks assessed using VAS and WOMAC scores in patients with painDETECT scores higher than 12 and in those with scores lower than 12, as an additional prespecified secondary outcome. The eMethods in Supplement 2 give detailed information on the secondary outcomes.

Secondary outcomes not reported here due to ongoing analyses include health economics, physical activity, gait characteristics, and body fat. Biochemical markers, a prespecified secondary outcome, were not assessed due to budget constraints.

### Adherence to Programs

Participants self-reported their adherence to the yoga or strengthening exercise program using an online logbook from baseline to 12 weeks and from baseline to 24 weeks. Adherence was defined as the percentage of prescribed sessions undertaken.

### AEs

Adverse events were monitored throughout the study and were defined as any issue persisting for more than 2 days or prompting the participant to seek additional treatment. All AEs were recorded and monitored throughout the study, and the chief investigator (B.A.) was notified within 24 hours if any serious AEs occurred.

### Sample Size Calculation

The power calculations for our study were based on a sample size that could have documented the superiority of yoga in comparison with strengthening by an amount that met the MCID. Using an MCID of 15 mm on the VAS pain scale (0 to 100), a 2-sided significance level of 0.05, 90% power, and an SD of 22.5 for the change in VAS pain score, a total of 98 participants was initially deemed to be necessary. Anticipating a dropout rate of around 20%, we adjusted the required participants to 123.

We determined a recruitment target of 126 participants to ensured adequate power for detecting the noninferiority of yoga compared with strengthening exercises. Noninferiority would be declared if the mean change in VAS knee pain score over 12 weeks in the yoga group was not significantly different from the mean change in the strengthening group, with a predefined margin of noninferiority set at 10 mm.^33^ Overall, 126 participants would provide 80% power to detect the noninferiority of yoga compared with strengthening exercise.

### Statistical Analysis

The primary analysis assessed the change in VAS knee pain score over 12 weeks. The primary analyses were performed from May 2023 to July 2024 based on the intention-to-treat principle and using all randomly assigned participants. We also conducted sensitivity analyses (based on only participants who completed the 12-week and 24-week follow-up periods) for the primary and secondary outcomes. A mixed-effects linear regression model was fitted with change from baseline in knee pain, WOMAC subscale (pain, function, and stiffness), patient global assessment, painDETECT score, PHQ-9 score, QOL-derived utility values, and physical performance measures, including fixed effects for treatment arm, week, and the treatment arm × week interaction. The correlated random variation within the repeated measures was addressed by using participant record identification as a random effect. Each model was adjusted for baseline values of corresponding outcomes, age, sex, and body mass index. Binary outcomes were analyzed using log binomial regression, fitted via a generalized linear model, and between-group comparisons were conducted using risk ratios and risk differences. The linear mixed-effects model included all participants and assumed the paradigm that the participants who were missing follow-up were missing at random.

A prespecified analysis was conducted to assess the noninferiority of yoga compared with strengthening exercises in managing knee pain over a 12-week follow-up period. The noninferiority margin was set at 10 mm, with noninferiority established if the 95% CI for the between-group change in VAS pain score did not cross this margin.^32^ To test the null hypothesis regarding the noninferiority of yoga compared with strengthening exercises in managing knee pain over 12 weeks, a 95% CI for the appropriate linear contrast was generated. Noninferiority was established if the lower boundary of the 95% CI excluded the noninferiority margin of 10 mm. The Cohen *d* effect size was calculated as the difference in treatment effects divided by the pooled SD estimated from the mixed model for repeated measures. Additional details on the statistical methods used can be found in the eMethods in Supplement 2. Statistical analysis was performed using Stata, version 18 (StataCorp LLC). Two-sided *P* < .05 was considered statistically significant, and no adjustment was made for multiple comparisons.

## Results

**Figure 1.**
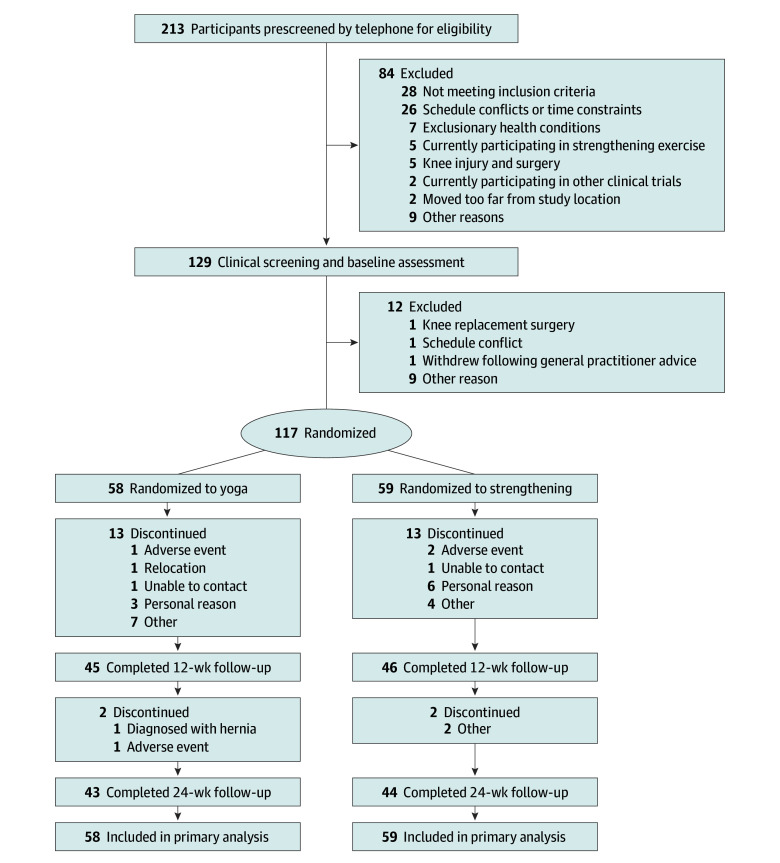
Screening, Randomization, and Completion of 12- and 24-Week Follow-Up

Figure 1 shows the participant flow through the study. Between June 2021 and June 2022, a total of 213 participants were screened, with 117 participants randomized to either the yoga program (n = 58) or the strengthening program (n = 59). Among them, 91 participants (77.8%) completed the week 12 assessment, and 87 participants (74.4%) completed the week 24 assessment. Baseline demographics and clinical characteristics of the 2 groups were comparable (Table 1). The mean (SD) age was 62.5 (8.3) years; 85 participants (72.6%) were female, and 32 participants (27.3%) were male. The mean (SD) baseline VAS knee pain score of 53.8 (16.0) indicated moderate knee pain.

**Table 1.** Baseline Characteristics of Participants by Group

Characteristic	Yoga (n = 58)		Strengthening (n = 59)	
	Total No.	Value^a^	Total No.	Value^a^
Age, y	58	61.2 (7.8)	59	63.7 (8.3)
Sex, No. (%)				
Female	58	41 (70.7)	59	44 (74.6)
Male	58	17 (29.3)	59	15 (25.4)
BMI	58	29.4 (5.8)	58	28.4 (4.9)
Waist-to-hip ratio	58	0.88 (0.08)	58	0.87 (0.07)
Symptoms				
Knee VAS, mm^b^	57	54.2 (15.1)	58	53.4 (16.8)
WOMAC^c^				
Total, mm	57	1011.3 (438.1)	57	962.3 (398.9)
Pain, mm	57	217.7 (88.2)	58	197.3 (80.9)
Function, mm	57	702.5 (327.3)	58	670.1 (297.0)
Stiffness, mm	57	91.0 (40.1)	57	94.1 (34.8)
Patient global assessment, mm^d^	57	35.7 (21.6)	57	33.1 (21.3)
Questionnaires				
PainDETECT^e^				
Score	54	7.2 (4.3)	55	7.6 (4.7)
No. (%)				
<12	54	46 (85)	55	43 (78)
≥12	54	8 (15)	55	12 (22)
PHQ-9 score^f^	57	4.6 (4.2)	57	3.9 (3.4)
Quality of life				
AQoL-8D utility score^g^	56	0.7 (0.1)	57	0.73 (0.1)
EQ-5D-5L index score^h^	57	0.8 (0.1)	57	0.83 (0.1)
Performance and strength				
Leg muscle strength, kg	57	66.3 (38.4)	58	59.6 (28.0)
30-s Chair stand test (repetition)	58	10.3 (2.7)	58	10.6 (2.1)
40-m Fast-paced walk test, m/s	58	1.8 (0.3)	57	1.8 (0.2)
Stair climb test, s	58	11.2 (5.3)	58	11.3 (3.4)
Laterality, No. (%)				
Unilateral symptoms	58	54 (93.1)	59	55 (93.2)
Bilateral symptoms	58	4 (6.9)	59	4 (6.8)
Pain medication, No. (%)				
Paracetamol	58	17 (29.3)	59	19 (32.2)
NSAIDs	58	19 (32.8)	59	17 (28.8)
Opioid	58	1 (1.7)	59	1 (1.7)
Topical analgesics	58	2 (3.4)	59	0
Gabapentin	58	1 (1.7)	59	1 (1.7)
Dietary supplement, No. (%)^i^	58	11 (19.0)	59	19 (32.2)

Abbreviations: AQoL-8D, Assessment of Quality of Life-8 Dimensions; BMI, body mass index (calculated as weight in kilograms divided by height in meters squared); EQ-5D-5L, European Quality of Life 5-Dimension 5-level; PHQ-9, Patient Health Questionnaire-9; NSAIDs, nonsteroidal anti-inflammatory drugs; VAS, visual analog scale; WOMAC, Western Ontario and McMaster Universities Osteoarthritis.

^a^ Data are presented as mean (SD) value unless otherwise indicated. Percentages may not total 100 because of rounding.

^b^ Scores range from 0 (no pain) to 100 (worst pain possible).

^c^ The WOMAC indexes range from 0 to 500 for pain, from 0 to 1700 for function, and from 0 to 200 for stiffness, with higher scores indicating more severe symptoms.

^d^ Assessed with the use of VAS; range 0 (very well) to 100 (very poor).

^e^ A 13-item screening survey for neuropathic pain with scores ranging from −1 to 38; less than 12 indicates negative symptoms of neuropathic pain, and 12 or greater indicates possible neuropathic pain.

^f^ Assesses depression; range is 0 to 27, with higher scores indicating more severe symptoms.

^g^ Obtained by AQoL utility formulae; scores range from −0.04 to 1.00, with −0.04 representing the worst possible health-related QOL; and 1.00, full health-related QOL.

^h^ Calculated using the UK utility index and range from −0.04 to 1.00, with −0.04 representing the worst possible health-related QOL; and 1.00, full health-related QOL.

^i^ Turmeric, glucosamine, chondroitin, algae extract, collagen, zingiber officinale, piper nigrum, ashwagandha, garlic, and fish oil (omega-3).

### Primary Outcome

There was no significant between-group difference in the change in mean VAS knee pain over 12 weeks (between-group difference, −1.1 mm [95% CI, −7.8 to 5.7 mm]; *P* = .76; Cohen *d*, −0.2 [95% CI, −1.6 to 1.2]) (Table 2 and eFigure 2 in Supplement 2). Compared with strengthening exercise, the VAS knee pain score reduction in the yoga group was within the prespecified noninferiority margin of 10 mm over 12 weeks (eFigure 4 in Supplement 2). Both groups showed improvement in VAS knee pain score over 12 weeks (yoga: −17.7 mm [95% CI, −22.4 to −13.0 mm]; strengthening: −16.7 mm [95% CI, −21.4 to −11.8 mm]). Primary outcome analysis in the per-protocol population (complete cases) at week 12 showed similar results (eTable 1 in Supplement 2).

**Table 2.** Change in Primary and Secondary Outcome Measures Over 12 and 24 Weeks for 117 Participants Within and Between the Yoga and Strengthening Groups

Outcome	Change within groups, mean (95% CI)		Difference in change between groups, mean (95% CI)	Cohen *d* (95% CI)	*P* value
	Yoga	Strengthening			
VAS knee pain score, mm^a^^,^^b^					
Week 12	−17.7 (−22.4 to −13.0)	−16.7 (−21.4 to −11.8)	−1.1 (−7.8 to 5.7)	−0.2 (−1.6 to 1.2)	.76
Week 24	−24.4 (−29.2 to −19.6)	−18.6 (−23.6 to −13.6)	−5.8 (−12.9 to 1.2)	−1.1 (−2.5 to 0.3)	.11
WOMAC pain index, mm^a^^,^^c^					
Week 12	−59.8 (−77.4 to −42.2)	−49.2 (−67 to −31.3)	−10.6 (−35.9 to 14.6)	−0.6 (−1.9 to 0.8)	.41
Week 24	−92.4 (−110.5 to −74.3)	−47.9 (−66.6 to −29.2)	−44.5 (−70.7 to −18.3)	−2.2 (−3.6 to −0.8)	.001
WOMAC function index, mm^a^^,^^c^					
Week 12	−183.2 (−243.1 to −123.4)	−120 (−181.8 to −58.9)	−63.1 (−149.3 to 23)	−0.9 (−2.3 to 0.5)	.15
Week 24	−268.5 (−330.2 to −206.9)	−129.6 (−193.3 to −65.9)	−139 (−228.3 to −49.7)	−1.9 (−3.3 to 0.6)	.002
WOMAC stiffness index, mm^a^^,^^c^					
Week 12	−26.6 (−35.4 to −17.8)	−18.4 (−27.6 to −9.3)	−8.2 (−21.1 to 4.7)	−0.8 (−2.2 to 0.5)	.22
Week 24	−40.3 (−49.4 to −31.2)	−22.6 (−32.1 to −13.2)	−17.6 (−30.9 to −4.3)	−1.8 (−3.1 to −0.4)	.009
Patient global assessment, mm^a^^,^^d^					
Week 12	−6.3 (−11.3 to −1.3)	−3.2 (−8.4 to 1.9)	−3.1 (−10.3 to 4.2)	−0.6 (−2.0 to 0.7)	.41
Week 24	−12.1 (−17.2 to −6.8)	−4.3 (−9.7 to 0.9)	−7.6 (−15.1 to −0.2)	−1.6 (−2.9 to −0.2)	.04
PainDETECT questionnaire^a^^,^^e^					
Week 12	−1.2 (−2.3 to −0.2)	−0.4 (−1.4 to 0.7)	−0.8 (−2.4 to 0.7)	−0.9 (−2.3 to 0.5)	.30
Week 24	−2.3 (−3.4 to −1.2)	−1.6 (−2.8 to −0.5)	−0.7 (−2.3 to 0.9)	−0.7 (−2.1 to 0.7)	.40
PHQ-9 score^a^^,^^f^					
Week 12	−1.06 (−1.68 to −0.45)	0.03 (−0.60 to 0.66)	−1.10 (−1.99 to −0.21)	−2.0 (−3.4 to −0.7)	.02
Week 24	−0.64 (−1.28 to 0.00)	0.09 (−0.75 to 0.56)	−0.55 (−1.47 to 0.37)	−1.0 (−2.4 to 0.4)	.24
AQol-8D utility score^g^^,^^h^					
Week 12	0.05 (0.03 to 0.07)	0.03 (0.00 to 0.05)	0.02 (−0.01 to 0.05)	1.0 (−0.3 to 2.4)	.20
Week 24	0.06 (0.04 to 0.08)	0.03 (0.00 to 0.05)	0.04 (0.00 to 0.07)	1.7 (0.3 to 3.1)	.03
EQ-5D-5L index score^g^^,^^i^					
Week 12	0.03 (0.01 to 0.05)	0.00 (−0.02 to 0.03)	0.03 (0.00 to 0.06)	1.4 (−0.02 to 2.8)	.10
Week 24	0.05 (0.03 to 0.07)	0.02 (0.00 to 0.04)	0.03 (0.00 to 0.06)	1.4 (0.01 to 2.8)	.08
Leg muscle strength, kg^g^					
Week 12	17.8 (13.3 to 22.3)	21.2 (16.8 to 25.6)	−3.4 (−9.8 to 2.9)	−0.8 (−2.2 to 0.6)	.29
Week 24	22.0 (17.4 to 26.5)	23.4 (18.9 to 27.8)	−1.4 (−7.9 to 5.0)	−0.4 (−1.8 to 0.9)	.67
30-s Chair stand test, repetitions^g^					
Week 12	1.9 (1.3 to 2.6)	1.8 (1.1 to 2.4)	0.2 (−0.8 to 1.1)	0.3 (−1.1 to 1.7)	.72
Week 24	3.5 (2.8 to 4.1)	3.2 (2.5 to 3.8)	0.3 (−0.6 to 1.2)	0.5 (−0.9 to 1.9)	.51
40-m Fast-paced walk test, m/s^g^					
Week 12	0.07 (0.04 to 0.1)	0.05 (0.02 to 0.09)	0.02 (−0.03 to 0.07)	0.6 (−0.7 to 2.0)	.44
Week 24	0.13 (0.09 to 0.17)	0.07 (0.04 to 0.11)	0.06 (0.01 to 0.11)	1.8 (0.4 to 3.2)	.03
Stair climb test, s^a^					
Week 12	−1.6 (−2.1 to −1.2)	−1.4 (−1.8 to −0.9)	−0.3 (−0.9 to 0.4)	−0.7 (−2.1 to 0.7)	.38
Week 24	−2.3 (−2.8 to −1.9)	−1.9 (−2.4 to −1.5)	−0.4 (−1.1 to 0.2)	−1.1 (−2.4 to 0.3)	.18
OARSI-OMERACT response, No. (%)					
Week 12	20 (44.4)	14 (32.6)	1.4 (0.8 to 2.3)	NA	.26
Week 24	24 (58.5)	19 (50.0)	1.2 (0.8 to 1.8)	NA	.45

Abbreviations: AQoL-8D, Assessment of Quality of Life-8 dimensions; EQ-5D-5L, European Quality of Life 5-Dimension 5-Level; PHQ-9, Patient Health Questionnaire-9; MCID, minimal clinically important difference; OARSI-OMERACT, Osteoarthritis Research Society International–Outcome Measure in Rheumatology Clinical Trial; NA, not applicable; VAS, visual analog scale; WOMAC, Western Ontario and McMaster Universities Osteoarthritis.

^a^ Negative within-group change indicates improvement. Negative between-group difference favors yoga.

^b^ Range is 0 (no pain) to 100 (worst pain possible) (MCID, 15).

^c^ Ranges from 0 to 500 for pain, 0 to 1700 for function, and 0 to 200 for stiffness; higher scores indicate more severe symptoms.

^d^ Assessed with VAS: 0 (very well) to 100 (very poor).

^e^ A 13-item screening survey for neuropathic pain; scores range from −1 to 38 (<12, negative neuropathic pain; ≥12, possible neuropathic pain).

^f^ Assesses depression. Range is 0 to 27; higher scores indicate more severe symptoms.

^g^ Positive within-group change indicates improvement. Positive between-group difference favors yoga.

^h^ Scores range from −0.04 to 1.00; higher scores indicate better QOL (MCID, 0.06).

^i^ Calculated by the UK utility index, ranging from −0.04 to 1.00; higher scores indicate better QOL.

### Secondary Outcomes

Secondary outcomes are presented in Table 2 and eTable 2 in Supplement 2. There was no significant between-group difference for the change in VAS pain score over 24 weeks (between-group difference: −5.8 mm [95% CI, −12.9 to 1.2 mm]; *P* = .11; Cohen *d*, −1.1 [95% CI, −2.5 to 0.3]).

Among the 27 secondary outcomes assessed over 12 and 24 weeks, 7 were statistically significant in favor of yoga. Over 12 weeks, there were no statistically significant differences between groups for any of the secondary outcomes apart from the PHQ-9 score favoring the yoga group (between-group difference, −1.1 [95% CI, −1.9 to −0.2]). However, this between-group difference was not seen over 24 weeks (Table 2).

**Figure 2.**
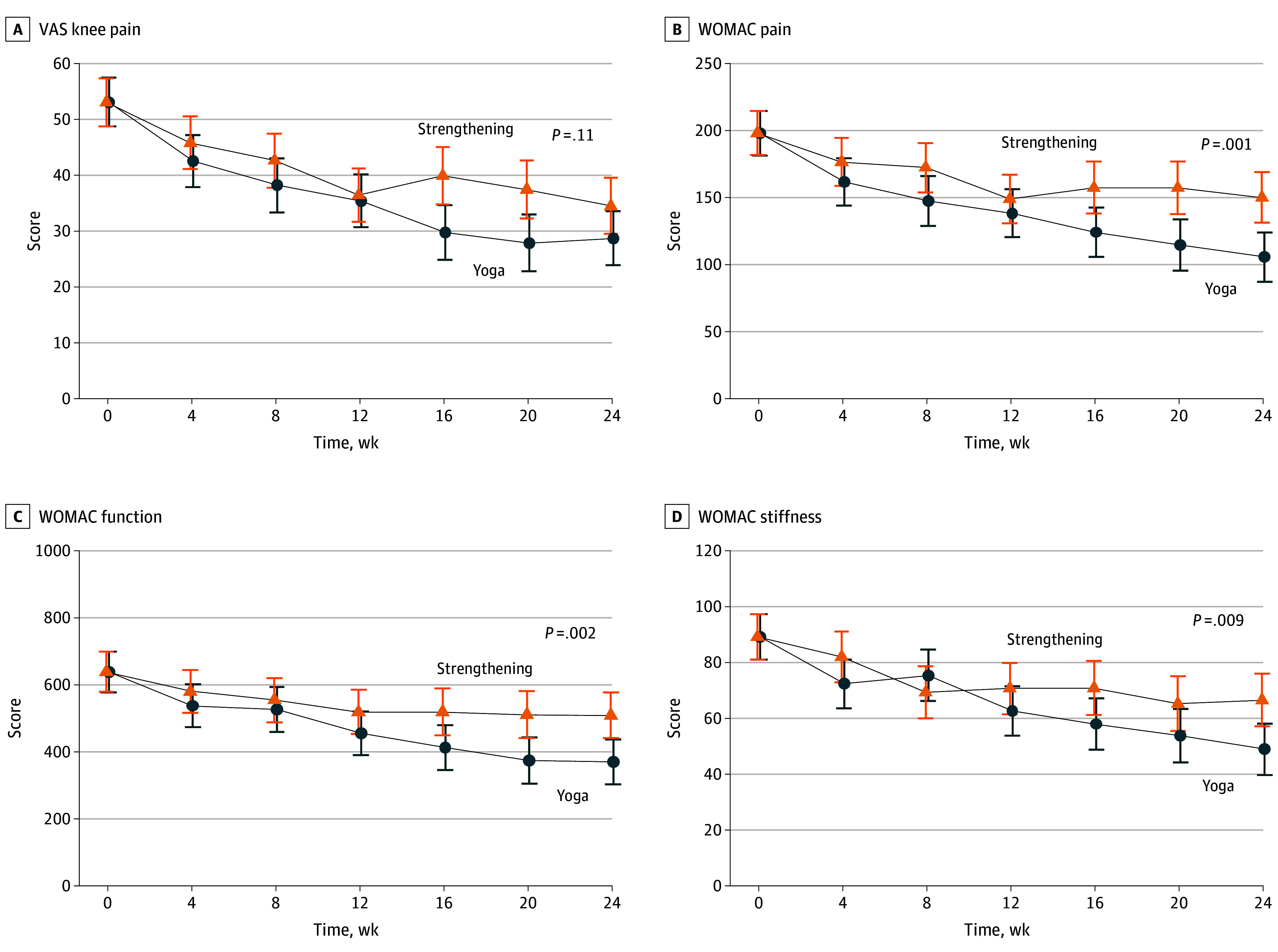
Mean Visual Analog Scale (VAS) Knee Pain and Western Ontario and McMaster Universities Osteoarthritis (WOMAC) Index Subscale Scores in the Yoga and Strengthening Groups Over 24 Weeks Values are adjusted means, error bars indicate 95% CIs, and *P* values correspond to between-group differences observed at 24 weeks. Data are estimates from linear mixed effect models with all 117 participants included in the analysis. Scores on the VAS range from 0 to 100, with higher scores indicating more severe symptoms. Scores on the WOMAC pain index ranged from 0 to 500; on the WOMAC function index, from 0 to 1700; and on the WOMAC stiffness index, from 0 to 200, all 3 with higher scores indicating more severe symptoms.

Over 24 weeks, the yoga group had modestly greater improvement (between-group differences) in WOMAC pain (−44.5 mm [95% CI, −70.7 to −18.3 mm]), function (−139 mm [95% CI, −228.3 to −49.7 mm]), and stiffness (−17.6 mm [95%CI, −30.9 to −4.3 mm]) scores; AQoL-8D–derived utility score (0.04 [95% CI, 0.0-0.07]; patient global assessment of symptoms; and the 40-m fast-paced walk test compared with the strengthening group. The 95% CI of the between-group difference in the change of other secondary outcomes over 24 weeks included the null (Table 2 and Figure 2). Results for the sensitivity analyses (based only on participants who completed week 12 and week 24) (eTable 1 in Supplement 2) were consistent with those presented in Table 2. Change in pain medication and supplement use is presented in eTable 4 in Supplement 2.

Adherence to yoga and strengthening exercises is shown in eTable 5 in Supplement 2. Over the 12-week period, both groups showed similar adherence, attending a mean (SD) of 25.1 (9.8) sessions in the yoga group and 26.0 (8.6) sessions in the strengthening exercise group. Mean (SD) completion rates were 69.8% (27.2%) for yoga and 71.9% (23.9%) for strengthening exercises over 12 weeks. Home exercise adherence from weeks 13 to 24 was higher in the yoga group than in the strengthening exercise group, with the mean (SD) percentage of prescribed sessions performed at 70.2% (28.5%) vs 60.0% (29.5%), respectively. From weeks 1 to 24, the mean (SD) weekly session attendance was 2.4 (0.9) for yoga and 2.3 (1.0) for strengthening exercises, and the groups attended a mean (SD) of 63.1% (31.1%) yoga sessions and 60.3% (27.8%) strengthening exercise sessions. Participant fidelity to the protocol was high in both groups (eTable 6 in Supplement 2), with no crossover between the yoga and strengthening exercise groups.

### A Priori Subgroup Analyses

The results of a priori subgroup analyses are given in eTable 3 and eFigure 3 in Supplement 2. The results showed no significant difference in the effects of yoga and strengthening exercises on VAS knee pain and WOMAC pain in participants with vs without possible neuropathic-like pain (eResults in Supplement 2).

### AEs

Adverse event rates are shown in Table 3. At least 1 AE was reported by 22 participants (37.9%) in the yoga group and 16 participants (27.1%) in the strengthening group over 24 weeks, mostly unrelated to the interventions. No serious AE was reported. There were 9 AEs in the yoga group (15.5%) and 4 AEs in the strengthening exercise group (6.8%) that were considered possibly and probably related to the interventions. The incidence of intervention-related AEs was higher in the yoga group. Aggravated knee pain occurred 7 times in the yoga group (n = 6) and 6 times in the strengthening exercise group (n = 5).

**Table 3.** Reported AEs Over 24 Weeks

Events	Participants, No. (%)	
	Yoga (n = 58)	Strengthening (n = 59)
Any AE^a^	32 (55.2)	26 (44.1)
≥1 AE	22 (37.9)	16 (27.1)
Serious AE	0	0
Death	0	0
Treatment related	9 (15.5)	4 (6.8)
Withdrawal related to AE	2 (3.4)	2 (3.4)
Type of AE		
Musculoskeletal and joint pain	16 (27.6)	13 (22.0)
Increased knee pain	6 (10.3)	5 (8.5)
Back pain	4 (6.9)	5 (8.5)
Hip pain	3 (5.2)	1 (1.7)
Other musculoskeletal-related AE^b^	3 (5.2)	2 (3.4)
Nonmusculoskeletal	14 (24.1)	13 (22.0)
Influenza virus infection	5 (8.6)	5 (8.5)
COVID-19	1 (1.7)	3 (5.1)
COVID-19 vaccine AE	1 (1.7)	1 (1.7)
Injury^c^	5 (8.6)	0
Knee replacement surgery	0	1 (1.7)
Cataract surgery	1 (1.7)	0
Other^d^	1 (1.7)	3 (5.1)

Abbreviation: AE, adverse event.

^a^ Could include multiple AEs in a single participant.

^b^ Included polymyalgia, ankle pain, heel pain, groin injury, and nerve pain in the lower leg.

^c^ Included falling, twisted knee injury, and hand injury.

^d^ Included headache, transient ischemic attack, and general unwellness.

## Discussion

In this randomized clinical trial, we investigated the comparative effectiveness of yoga and strengthening exercise for treating knee OA over 24 weeks. We found that the change in VAS knee pain over 12 weeks was not significantly different between the 2 groups and was within our prespecified noninferiority margin of 10 mm. Both groups reported pain reduction following treatment that was of a magnitude deemed to be clinically relevant. Over 12 weeks, none of the secondary outcome measures showed differences between the 2 groups except for a small improvement in depression favoring the yoga group. At the 24-week assessment, modestly greater improvements in several outcomes, including WOMAC pain, function, and stiffness scores; health-related QOL; patient global assessment; and the 40-m fast-paced walk test, were reported for the yoga group compared with the strengthening group.

There are several possible explanations for our findings. Our study compared yoga with an active, evidence-based strengthening exercise, distinguishing it from previous studies that compared yoga with traditional strengthening exercise or a combination of aerobic and strengthening exercise. Another potential reason could be a type II error. However, the study had statistical power exceeding 90% to detect clinically important differences if these had been present. Perhaps the placebo effect should not be underestimated,^34^ as both yoga and strengthening exercises may have induced a placebo effect, leading to improvements in outcomes across both groups. This effect could mask any true difference in patient-reported outcomes between the interventions, as individuals in both groups may have perceived benefits from engaging in structured exercises.^35^ However, it is important to note that we compared 2 active interventions, and even if a placebo effect was present, comparability between the groups was maintained.

Our study was designed as a comparative effectiveness trial and had the recommended exercise therapy (strengthening exercise) as the control arm. We ensured that any potential contextual effects were comparable between the 2 groups by maintaining a similar number of patient-therapist contacts during the intervention period. Unlike our findings for our primary outcome, Cheung et al^12^ reported a significantly greater reduction in knee pain (VAS score) with yoga compared with a combination of aerobic and strengthening exercise over 8 weeks. However, similar to our findings at 24 weeks, they reported modest benefit of yoga compared with a combination of aerobic and strengthening exercise for WOMAC pain and function and VAS pain scores. A study assessing an unsupervised online yoga program demonstrated modest improvements in physical function, though below the MCID, and no significant reduction in knee pain over 12 weeks compared with an online education group among individuals with knee OA.^36^

Our secondary outcomes included a prolonged home-based intervention period of up to 24 weeks, and we found modest statistically significant differences in multiple secondary outcomes, suggesting a medium-term benefit of yoga compared with strengthening exercise. Home adherence to yoga was higher than for strengthening exercise, which might be partly responsible for the difference in secondary outcomes at 24 weeks. These results are similar to a recent meta-analysis that reported yoga is superior in mitigating pain, enhancing function, and alleviating stiffness among individuals with knee OA.^8^ The secondary outcomes highlight the potential for yoga to be a viable medium-term alternative to strengthening exercise for improving pain, function, stiffness, and QOL in individuals with knee OA. Furthermore, the observed reduction in depression in the yoga group over 12 weeks shows the potential mental health benefits of yoga compared with strengthening exercise, as depression is a common comorbidity in knee OA and yoga may be well-suited for patients with both conditions.

Our results indicated a modest effect of yoga compared with strengthening exercise over 24 weeks. However, this effect size was smaller than that reported in a meta-analysis comparing yoga with exercise (combination of aerobic and strengthening exercise, strengthening exercise, and physiotherapy-based exercise) for OA,^8^ possibly indicating a more realistic effect when compared with an active intervention in knee OA.

Both groups demonstrated good safety and adherence overall; however, the incidence of AEs was slightly higher in the yoga group. This may be attributed to participants being unfamiliar with some of the yoga poses. Additionally, some AEs in the yoga group, such as falls, were unrelated to the intervention.

### Strengths and Limitations

A strength of our study is that we compared yoga with an active evidence-based strengthening exercise program. Previous studies have predominantly explored the benefits of yoga compared with an attention control or health education group,^13,15,17,36,37^ but our approach offered several advantages. First, it allowed us to directly assess the relative effects of 2 active interventions, providing insights into which approach might yield better outcomes for knee OA management. Second, this study added yoga to the clinician toolbox as an alternative exercise option, supporting shared decision-making with patients on self-management strategies. The holistic approach of yoga, emphasizing flexibility, balance, and mental well-being, may offer promise for individuals with knee OA, especially those who find conventional strengthening exercises challenging. While some yoga poses may initially cause discomfort for some participants, it complements strengthening exercises as a viable option to improve physical and mental health. Moreover, the active comparator design improved the methodologic quality of the study by making comparable contextual effects between groups that could bias the outcomes. We also powered our study to detect both superiority and noninferiority. A notable strength is that both the evidence-based yoga program and the strengthening exercise program were designed by experts in the field.

Some limitations warrant caution in interpreting our findings. We randomized fewer participants to receive the interventions than the planned sample (117 instead of 126) due to the COVID-19 pandemic. However, we had higher than 90% power to detect superiority, even with 117 participants. This was a program designed specifically for patients with arthritis and does not reflect the safety or effectiveness of general community-based yoga classes. Participants were recruited from a single Australian state, which may limit the generalizability of the findings globally. While our findings are promising, further research is needed to investigate the long-term effects of yoga and strengthening exercises beyond the 24-week period, providing insights into the sustainability of benefits. Additionally, investigating the mechanisms underlying the observed improvements, such as pain, function, stiffness, physical performance, and depression, could yield a deeper understanding of how these interventions exert their effects.

## Conclusions

In this randomized clinical trial, a 12-week yoga program did not significantly reduce knee pain compared with a strengthening program among adults with knee OA. The yoga program was noninferior to the strengthening program, as both groups reported pain reductions that were deemed to be clinically relevant over 12 weeks. Secondary outcomes favoring yoga, including depression, health-related QOL, patient global assessment, the 40-m fast-paced walk test, and WOMAC pain, function, and stiffness scores, should be interpreted as exploratory. Overall, these findings suggest that integrating yoga as an alternative or complementary exercise option in clinical practice may help in managing knee OA.

## Notes

Supplement 1.
Trial Protocol

Supplement 2. **eMethods.** Additional details **eResults.** A priori subgroup analyses **eTable 1.** Sensitivity analyses based on the per-protocol population on change in study endpoints between the yoga and strengthening groups over 12- and 24-weeks **eTable 2.** Change in knee symptoms (VAS & WOMAC) from baseline to all time points **eTable 3.** Change in knee symptoms (VAS & WOMAC) from baseline to all time points in participants with possible neuropathic pain and without neuropathic pain at baseline **eTable 4.** Change in pain medication and supplements over 12- and 24-weeks **eTable 5.** Adherence measures over the first 12 and 24 weeks for participants in yoga and strengthening groups **eTable 6.** Participants' fidelity to intervention protocol across groups, reported as n (%) unless otherwise indicated **eFigure 1.** Study schedule of the trial **eFigure 2.** Mean VAS knee pain and WOMAC scores (95% CI) in the yoga and strengthening groups over 12 weeks **eFigure 3.** Change in VAS and WOMAC scores over 12 and 24 weeks, stratified by baseline PainDETECT score, for the yoga and strengthening groups **eFigure 4.** Non-inferiority analysis of VAS knee pain score at 12 weeks **eAppendix.** Yoga and strengthening exercises: intervention description/manual

Supplement 3.
Data Sharing Statement

## References

[1] Steinmetz JD, Culbreth GT, Haile LM, ; GBD 2021 Osteoarthritis Collaborators. Global, regional, and national burden of osteoarthritis, 1990-2020 and projections to 2050: a systematic analysis for the Global Burden of Disease Study 2021. Lancet Rheumatol. 2023;5(9):e508-e522. doi:10.1016/S2665-9913(23)00163-7 37675071 PMC10477960

[2] Long H, Liu Q, Yin H, . Prevalence trends of site-specific osteoarthritis from 1990 to 2019: findings from the Global Burden of Disease Study 2019. Arthritis Rheumatol. 2022;74(7):1172-1183. doi:10.1002/art.42089 35233975 PMC9543105

[3] Duong V, Oo WM, Ding C, Culvenor AG, Hunter DJ. Evaluation and treatment of knee pain: a review. JAMA. 2023;330(16):1568-1580. doi:10.1001/jama.2023.19675 37874571

[4] Guideline for the management of knee and hip osteoarthritis second edition. The Royal Australian College of General Practitioners. Accessed March 3, 2025. https://www.racgp.org.au/FSDEDEV/media/documents/Clinical%20Resources/Guidelines/Joint%20replacement/Guideline-for-the-management-of-knee-and-hip-OA-2nd-edition.pdf

[5] Zeng CY, Zhang ZR, Tang ZM, Hua FZ. Benefits and mechanisms of exercise training for knee osteoarthritis. Frontiers in Physiology. 2021;12. 10.3389/fphys.2021.794062 PMC8716769 34975542

[6] Büssing A, Ostermann T, Lüdtke R, Michalsen A. Effects of yoga interventions on pain and pain-associated disability: a meta-analysis. J Pain. 2012;13(1):1-9. doi:10.1016/j.jpain.2011.10.001 22178433

[7] Wren AA, Wright MA, Carson JW, Keefe FJ. Yoga for persistent pain: new findings and directions for an ancient practice. Pain. 2011;152(3):477-480. doi:10.1016/j.pain.2010.11.017 21247696 PMC3040510

[8] Lauche R, Hunter DJ, Adams J, Cramer H. Yoga for osteoarthritis: a systematic review and meta-analysis. Curr Rheumatol Rep. 2019;21(9):47. doi:10.1007/s11926-019-0846-5 31338685

[9] Zhang Q, Young L, Li F. Network meta-analysis of various nonpharmacological interventions on pain relief in older adults with osteoarthritis. Am J Phys Med Rehabil. 2019;98(6):469-478. doi:10.1097/PHM.0000000000001130 31094857

[10] Geenen R, Overman CL, Christensen R, . EULAR recommendations for the health professional’s approach to pain management in inflammatory arthritis and osteoarthritis. Ann Rheum Dis. 2018;77(6):797-807. doi:10.1136/annrheumdis-2017-212662 29724726

[11] Kolasinski SL, Neogi T, Hochberg MC, . 2019 American College of Rheumatology/Arthritis Foundation guideline for the management of osteoarthritis of the hand, hip, and knee. Arthritis Care Res (Hoboken). 2020;72(2):149-162. doi:10.1002/acr.24131 31908149 PMC11488261

[12] Cheung C, Wyman JF, Bronas U, McCarthy T, Rudser K, Mathiason MA. Managing knee osteoarthritis with yoga or aerobic/strengthening exercise programs in older adults: a pilot randomized controlled trial. Rheumatol Int. 2017;37(3):389-398. doi:10.1007/s00296-016-3620-2 27913870 PMC5569242

[13] Cheung C, Wyman JF, Resnick B, Savik K. Yoga for managing knee osteoarthritis in older women: a pilot randomized controlled trial. BMC Complement Altern Med. 2014;14(1):160. doi:10.1186/1472-6882-14-160 24886638 PMC4038088

[14] Kuntz AB, Chopp-Hurley JN, Brenneman EC, . Efficacy of a biomechanically-based yoga exercise program in knee osteoarthritis: a randomized controlled trial. PLoS One. 2018;13(4):e0195653. doi:10.1371/journal.pone.0195653 29664955 PMC5903657

[15] Moonaz SH, Bingham CO III, Wissow L, Bartlett SJ. Yoga in sedentary adults with arthritis: effects of a randomized controlled pragmatic trial. J Rheumatol. 2015;42(7):1194-1202. doi:10.3899/jrheum.141129 25834206 PMC4490021

[16] Ebnezar J, Nagarathna R, Yogitha B, Nagendra HR. Effect of integrated yoga therapy on pain, morning stiffness and anxiety in osteoarthritis of the knee joint: a randomized control study. Int J Yoga. 2012;5(1):28-36. doi:10.4103/0973-6131.91708 22346063 PMC3276929

[17] Park J, McCaffrey R, Newman D, Liehr P, Ouslander JG. A pilot randomized controlled trial of the effects of chair yoga on pain and physical function among community-dwelling older adults with lower extremity osteoarthritis. J Am Geriatr Soc. 2017;65(3):592-597. doi:10.1111/jgs.14717 28008603 PMC5357158

[18] Bannuru RR, Osani MC, Vaysbrot EE, . OARSI guidelines for the non-surgical management of knee, hip, and polyarticular osteoarthritis. Osteoarthritis Cartilage. 2019;27(11):1578-1589. doi:10.1016/j.joca.2019.06.011 31278997

[19] A randomised comparative effectiveness trial of yoga and strengthening exercise for knee osteoarthritis. ANZCTR.org identifier: ACTRN12621000066886. Updated January 1, 2021. Accessed November 8, 2023. https://www.anzctr.org.au/Trial/Registration/TrialReview.aspx?id=380369&isReview=true

[20] Singh A, Aitken D, Moonaz S, . A randomised controlled trial of *yog*a and strengthening exercise for knee osteo*a*rthritis: protocol for a comparative effectiveness trial (YOGA Trial). J Funct Morphol Kinesiol. 2022;7(4):84. doi:10.3390/jfmk7040084 36278745 PMC9624302

[21] Calvert M, Blazeby J, Altman DG, Revicki DA, Moher D, Brundage MD; CONSORT PRO Group. Reporting of patient-reported outcomes in randomized trials: the CONSORT PRO extension. JAMA. 2013;309(8):814-822. doi:10.1001/jama.2013.879 23443445

[22] McAlindon TE, Driban JB, Henrotin Y, . OARSI clinical trials recommendations: design, conduct, and reporting of clinical trials for knee osteoarthritis. Osteoarthritis Cartilage. 2015;23(5):747-760. doi:10.1016/j.joca.2015.03.005 25952346

[23] Tubach F, Ravaud P, Martin-Mola E, . Minimum clinically important improvement and patient acceptable symptom state in pain and function in rheumatoid arthritis, ankylosing spondylitis, chronic back pain, hand osteoarthritis, and hip and knee osteoarthritis: results from a prospective multinational study. Arthritis Care Res (Hoboken). 2012;64(11):1699-1707. doi:10.1002/acr.21747 22674853

[24] Dobson F, Hinman RS, Roos EM, . OARSI recommended performance-based tests to assess physical function in people diagnosed with hip or knee osteoarthritis. Osteoarthritis Cartilage. 2013;21(8):1042-1052. doi:10.1016/j.joca.2013.05.002 23680877

[25] Wright AA, Cook CE, Baxter GD, Dockerty JD, Abbott JH. A comparison of 3 methodological approaches to defining major clinically important improvement of 4 performance measures in patients with hip osteoarthritis. J Orthop Sports Phys Ther. 2011;41(5):319-327. doi:10.2519/jospt.2011.3515 21335930

[26] AQoL-8D 2014. Assessment of Quality of Life. September 1, 2023. https://www.aqol.com.au/index.php/aqolinstruments?id=58

[27] EQ-5D-5L. EuroQol Research Foundation. Accessed September 1, 2023. https://euroqol.org/eq-5d-instruments/eq-5d-5l-about/

[28] Hawthorne G, Osborne R. Population norms and meaningful differences for the Assessment of Quality of Life (AQoL) measure. Aust N Z J Public Health. 2005;29(2):136-142. doi:10.1111/j.1467-842X.2005.tb00063.x 15915617

[29] Löwe B, Kroenke K, Herzog W, Gräfe K. Measuring depression outcome with a brief self-report instrument: sensitivity to change of the Patient Health Questionnaire (PHQ-9). J Affect Disord. 2004;81(1):61-66. doi:10.1016/S0165-0327(03)00198-8 15183601

[30] Freynhagen R, Baron R, Gockel U, Tölle TR. painDETECT: a new screening questionnaire to identify neuropathic components in patients with back pain. Curr Med Res Opin. 2006;22(10):1911-1920. doi:10.1185/030079906X132488 17022849

[31] Pham T, van der Heijde D, Altman RD, . OMERACT-OARSI initiative: Osteoarthritis Research Society International set of responder criteria for osteoarthritis clinical trials revisited. Osteoarthritis Cartilage. 2004;12(5):389-399. doi:10.1016/j.joca.2004.02.001 15094138

[32] Committee for Proprietary Medicinal Products. Points to consider on switching between superiority and non-inferiority. Br J Clin Pharmacol. 2001;52(3):223-228. doi:10.1046/j.1365-2125.2001.01397-3.x 11560553 PMC2014556

[33] Ehrich EW, Davies GM, Watson DJ, Bolognese JA, Seidenberg BC, Bellamy N. Minimal perceptible clinical improvement with the Western Ontario and McMaster Universities osteoarthritis index questionnaire and global assessments in patients with osteoarthritis. J Rheumatol. 2000;27(11):2635-2641. 11093446

[34] Hafliðadóttir SH, Juhl CB, Nielsen SM, . Placebo response and effect in randomized clinical trials: meta-research with focus on contextual effects. Trials. 2021;22(1):493. doi:10.1186/s13063-021-05454-8 34311793 PMC8314506

[35] Benedetti F. Placebo and the new physiology of the doctor-patient relationship. Physiol Rev. 2013;93(3):1207-1246. doi:10.1152/physrev.00043.2012 23899563 PMC3962549

[36] Bennell KL, Schwartz S, Teo PL, . Effectiveness of an unsupervised online yoga program on pain and function in people with knee osteoarthritis: a randomized clinical trial. Ann Intern Med. 2022;175(10):1345-1355. doi:10.7326/M22-1761 36122378

[37] Park J, Newman D, McCaffrey R, Garrido JJ, Riccio ML, Liehr P. The effect of chair yoga on biopsychosocial changes in English- and Spanish-speaking community-dwelling older adults with lower-extremity osteoarthritis. J Gerontol Soc Work. 2016;59(7-8):604-626. doi:10.1080/01634372.2016.1239234 27661469 PMC5177482

