# Supplementary material for: Yoga or Strengthening Exercise for Knee Osteoarthritis: A Randomized Clinical Trial
Source: JAMA Netw Open. 2025 Apr 8;8(4):e253698. doi: 10.1001/jamanetworkopen.2025.3698 (PMC11979726; doi:10.1001/jamanetworkopen.2025.3698)
Supplement: Supplement 1. — Trial Protocol [file jamanetwopen-e253698-s001.pdf]

Supplement 1

Study Protocol: A randomised comparative effectiveness trial of YOGa and strengthening exercise for knee osteoArthritis (YOGA Trial)

TABLE OF CONTENTS

This supplement includes the following documents:

1. Original study protocol approved by the ethics committee.
2. Summary of changes made to the study protocol.
3. Statistical Analysis Plan.
4. Summary of changes made to the Statistical Analysis Plan.

Study Protocol: A randomised comparative effectiveness trial of YOGa and strengthening exercise for knee osteoArthritis

(YOGA Trial)

Version number 1.6, dated 12 Sep 2022

Principle investigator
Dr Benny Antony[1], Senior Research Fellow

Chief investigators
Dr Steffany Moonaz[2], Director of Clinical and Academic Research
Prof Kim Bennell[3], Director of the Centre for Health, Exercise and Sports Medicine
Prof Graeme Jones[1], Rheumatologist and Professorial Research Fellow
Assoc Prof Dawn Aitkens[1], Research Fellow and Head of Menzies Musculoskeletal Unit
Prof Andrew Palmer[1], Head of the Menzies Health Economics Unit
Prof Leigh Blizzard[1], Head of the Menzies Statistical Unit

Affiliations
[1]Menzies Institute for Medical Research, University of Tasmania
[2]Maryland University of Integrative Health, USA
[3]Department of Physiotherapy, School of Health Sciences, Faculty of Medicine, Dentistry and Health Sciences, University of Melbourne

Funding
This trial is funded by the Rebecca L Cooper Medical Research Foundation.

Summary of proposal in layman's terms

Osteoarthritis is a common joint disorder for which there is no cure. Knee osteoarthritis is the most common form of osteoarthritis. Exercise is a core treatment for knee osteoarthritis. Muscle strengthening exercise has been shown to improve pain and function and is recommended by clinical guidelines. Yoga is a mind-body exercise intervention that can provide increased flexibility, muscle strength, physical balancing, improved fitness, and has demonstrated pain-relieving properties. Yoga, with its potential for improving musculoskeletal function, nervous system, and sympathetic activity, may therefore have greater benefits than commonly used strengthening exercise.

Previous studies of yoga in knee osteoarthritis patients have been of low-quality with a high risk of bias and did not perform subgroup analyses to identify patients who may specifically benefit from yoga. This randomised controlled trial aims to compare the effectiveness of the 12-week yoga program (12 weeks of yoga (3x/week)) on knee pain in knee osteoarthritis patients with a priori subgroup analysis of knee osteoarthritis patients with neuropathic pain compared to strengthening exercise. If successful, yoga can be easily implemented as a complementary treatment along with conventional treatment to improve the management of knee osteoarthritis.

Abstract:
Osteoarthritis (OA) is one of the major causes of pain and disability in the Australian population. Knee OA is the most common form of OA. Currently, there is no disease-modifying treatment available for knee OA.

Knee OA can be categorised by its manifestation characteristics at the structural and symptomatic levels. While biomarkers including MRI markers have enabled stratification of OA patients based on structural abnormalities, analyses of data from large observational studies and clinical trials have suggested the existence of distinct pain phenotypes (1-3) in knee OA. The pain phenotype can be defined as a subtype of OA that shares a distinct underlying pain mechanism. The pain phenotype of OA is complex and may be influenced by psychological factors (e.g. depression, pain-related fear, and anxiety) (4-6) and central pain processing (central sensitisation) (7, 8). The common discrepancies observed between the symptom severity and structural findings of knee OA may also be attributed to these factors (9). Pain is maintained by nociceptive, central, or neuropathic inputs and there is evidence of these in OA (10-12). Various studies using painDETECT have reported a 5-50% prevalence of neuropathic pain type in knee OA populations (10, 13).

Current pharmacological treatments for patients with symptomatic OA are palliative and primarily focus on pain relief. There is evidence that non-pharmacological treatments, such as exercise therapy (aerobic, muscle-strengthening, etc.) improve pain, function, depression, and quality of life (QoL) in people with OA (14). However, many studies are of low quality and had a high risk of bias, mainly due to comparing to a usual care arm (14-16). Increasing evidence demonstrates that neuropathic pain may contribute to the pain experience for a subgroup of OA patients. Therefore, it is possible that OA patients will show greater benefit from centrally-acting non-pharmacological therapies such as yoga (7, 17-20).

Yoga is a mind-body therapy, and the physical component of yoga provides exercise that is consistent with recommendations for knee OA (21, 22), while the mind component has the potential to increase psychological well-being and reduce stress(23, 24), which can influence central sensitisation. Only a few studies have evaluated the effect of yoga in patients with OA. Current guidelines for the treatment of knee OA suggest a 'conditional recommendation' for yoga as an adjunctive form of exercise, and only for short-term management. The guidelines highlighted the lack of evidence and the poor quality of the existing evidence. A systematic review of yoga intervention studies (including RCT and non-RCT) in OA reported a significant effect on pain and mobility. However, they reported a lack of data on QoL and mental health (25). Small sample sizes, shorter duration of follow-up, lack of an active comparator, and poor methodological quality of previous studies prevent definitive conclusions.

The primary aim of this 24 weeks randomised controlled trial (RCT) is to investigate the effectiveness of a 12-week group-based *yoga program* (12 weeks of yoga (3x/week)) compared to a group-based *strengthening exercise program* (3x/week) on the knee pain (assessed using VAS) in knee OA patients over 12 weeks. We hypothesise that a group-based yoga program will be superior to the group based strengthening program.

A secondary aim is to assess whether the effects are moderated by the presence of neuropathic pain such that individuals with neuropathic pain respond better to a yoga program compared to strengthening exercise program than those without neuropathic pain.

If successful, yoga can be easily implemented as a complementary treatment along with conventional treatment to improve the management of knee OA.

Background:

Osteoarthritis (OA) is the most common joint disorder in the world and affects over 2.2 million people in Australia, with a direct health expenditure of $3.5 billion in 2015–16 representing 28% of overall expenditure on musculoskeletal conditions and 3% of total disease expenditure (26). Given the ageing and increasingly obese population, it is not surprising that the national joint replacement registry (AOANJRR) revealed a 128% increase in knee replacement procedures from 2003 to 2018 (27).

Despite its substantial disease burden, there is no approved disease-modifying treatment available for OA. Traditional pharmaceutical therapies, i.e. analgesics, corticosteroids, and non-steroidal anti-inflammatory drugs (NSAIDs) are, at best, only modestly effective for pain and show no effect on joint structures, with end-stage OA treated with costly joint replacements. There is evidence for non-pharmacological treatments, such as exercise (aerobic, muscle-strengthening, etc.) and physical therapy which suggests that it reduces pain, improves function, and quality of life (QoL) in OA populations; however, many studies are of low quality and high risk of bias mainly by comparing to a usual care arm (15, 16, 28).

We have shown that OA is a heterogeneous disease with multiple phenotypes that may require somewhat different approaches for each patient in order to optimise treatment. Analyses of large observational and clinical trial data have suggested the existence of distinct pain phenotypes in knee OA (1-3, 13). This pain phenotype of OA can be defined as a subtype of OA that shares a distinct underlying pain mechanism. The pain phenotype of OA is complex and may be influenced by psychological factors (e.g. depression, pain-related fear, and anxiety) (4-6) and neural sensitisation (7, 8). The occasional discrepancies observed between the symptom severity and structural findings of knee OA and the existence of persistent pain even after total knee replacement also indicates these factors (9, 29).

There is increasing clinical evidence to suggest that neuropathic pain may contribute to the pain experience in OA patients (10). Given that central sensitisation plays a role in chronic neuropathic pain, centrally-acting physical exercises may be effective (7, 13, 17). The animal models of severe OA have shown features of neuropathy and link the associated nerve damage to the progression or severity of OA (30, 31). Preliminary studies in OA patients with neuropathic pain phenotype have demonstrated that treatment with the non-standard analgesics such as tricyclic antidepressants and gabapentinoids, may be more effective than conventional treatments (13).

Yoga is a mind-body exercise constituting physical postures (*asanas*), breathing practices (*pranayama*), meditative mental focus (*dhyana*), and relaxation as the four standard components (32). With the increasing prevalence of yoga use globally, it is becoming a more popular exercise to achieve and maintain well-being and health (33-35). Yoga has been used clinically as a therapeutic intervention for improvement in pain, stiffness, swelling, and mobility symptoms in older adults and is recommended by several clinical guidelines for individuals with OA (21, 36-40). However, the current guidelines for the treatment of knee OA suggest a ‘conditional recommendation’ for yoga as an adjunctive form of exercise, and only for short-term management. The guidelines highlighted the lack of evidence and the poor quality of the existing evidence.

The mechanisms of action of yoga in OA may extend beyond the exercise component. The physical component of yoga provides exercise that is consistent with recommendations for knee OA, while the mind component of yoga has the potential to increase psychological well-being and reduce stress, improve function, and alleviate OA associated pain (41). A previous

trial has reported that sitting meditation without any physical activity component improved function in OA patients (42). A network meta-analysis assessing the effects of various non-pharmacological interventions on pain relief in patients with OA reported yoga as the most effective intervention for patients and with a high adherence rate (43). Another recent systematic review synthesizing evidence of effectiveness and safety of yoga for OA patients identified nine randomized controlled trials of low methodological quality and potential risk of bias and provided a weak recommendation to support the use of yoga in knee OA (44). Hence, given the relative scarcity of robust, high-quality conclusive evidence for yoga in OA, we aim to evaluate the effect of yoga, compared to strengthening exercise in patients with knee OA and to assess whether the effects are greater with yoga in the subgroup of patients with neuropathic pain than in those without neuropathic pain.

Comparative effectiveness research (CER) is a research methodology that reduces the translation gap between clinical research and everyday clinical practice to enable the delivery of the most cost-effective health care to the target population (45). Although CER evidence can be synthesized in the form of systematic reviews, meta-analysis, and decision modelling using the existing real-world data (RWD) [e.g. administrative claims, electronic medical records, registries and other clinical cohorts, and case-control studies], RCTs are still the most rigorous method of generating highest quality CER evidence (46). Moreover, physical therapy/exercise, of which yoga is a part, is the top priority research area for assessing the patient-centred comparative safety and effectiveness of therapies in the OA population (47). Hence, we plan to include CER components by comparing two active physical exercise programs (i.e. *yoga program* or *strengthening exercise program*) and assess the patients' reported outcome and subsequent cost-effectiveness of *yoga program* versus *strengthening exercise program* in the symptomatic knee OA population. We hypothesise that yoga will outperform strengthening exercise and we aim to demonstrate that, compared with strengthening exercise, yoga may be an effective and cost-effective therapy for managing pain and reducing the functional limitations that impact the QoL in patients with knee OA.

Study aims and hypotheses:
The primary aim of this 24-week RCT is to compare the effectiveness of a 12-week *yoga program* (12 weeks of group-based yoga (3x/week)) on reducing knee pain (assessed using VAS) over 12 weeks in knee OA patients compared to a 12-week group-based *strengthening exercise program* (3x/week). We hypothesise that yoga will outperform strengthening exercise and we aim to demonstrate that, compared with strengthening exercise, yoga may be an effective and cost-effective therapy for managing pain and reducing the functional limitations that impact the QoL in patients with knee OA. A secondary aim is to assess whether the presence of neuropathic pain moderates the effects of yoga compared to strengthening exercise.

Methods:
Study design:
Single centre comparative effectiveness RCT.

Study participants:
We will recruit 126 knee OA participants with knee pain from the southern Tasmania region of Australia. We will use a proven recruitment strategy, including collaboration with general practitioners, specialist rheumatologists, orthopaedic surgeons, physiotherapists, and advertising through radio, TV, newspaper, social media, community notice boards, hospital notice boards, community newsletters (e.g. Arthritis Tasmania), local press and newsletters (e.g. council newsletters such as the Glenorchy Gazette, Eastern Shore Sun, Hobart City Council equivalent), physiotherapy practice's (e.g. BodySystem, Allcare physiotherapy, Integrated Physiotherapy, etc.) and GPs and exercise physiologist practising in the region. This is the approach we have taken to successfully recruit participants for the large community-based trials across a range of musculoskeletal conditions. We will also reach out to the participants of the previous studies for knee osteoarthritis to seek their interest in participating in this trial.

Inclusion criteria:
1. Aged ≥40 years
2. Knee pain on most days for at least six months
3. VAS knee pain intensity of ≥40 mm in the last month.
4. Meet American College of Rheumatology (ACR) clinical criteria for the diagnosis of knee OA
5. Be willing to participate in a group yoga program or group strengthening exercise program three times per week for 12-weeks and can attend on the days/times of the week that scheduled classes are running.

Exclusion criteria:
1. Patients currently or in the past three months engaged in strengthening exercise or yoga program
2. Other forms of inflammatory arthritis (especially rheumatoid arthritis and gout).
3. A significant knee injury that required treatment within the last six months.
4. Arthroscopy or open surgery in the index knee in the last six months or planned in the next 6-8 months.
5. Injections of corticosteroids (last three months) or hyaluronic acid (last six months) in the index knee.
6. Pregnancy or breastfeeding.
7. Currently participating in any other drug/device/exercise clinical trial related to OA.
8. Presence of any serious medical illness or condition that may preclude a 24-week follow up.

9. Any condition that precludes safe participation in exercise (i.e. fails the safety for exercise clearance; see below for the procedure for this).
10. Unable to walk without a gait aid.
11. Inability to provide informed consent in English.
12. Plan to start exercise or another new treatment for knee OA in the next six months.
13. Planned absences (e.g. trips away) of >2 weeks maximum during the 12-week period.

Screening:
Volunteers will be telephone screened for general eligibility and invited to Menzies for a screening visit which comprises of symptom assessments, medication history questionnaires, safety for exercise clearance, and clinical assessment for ACR criteria. In the case of bilateral eligible knees, the most symptomatic knee will be the study knee. In the case of bilaterally eligible knees with equal symptoms, the dominant knee will be studied.

Safety for exercise clearance:
During the screening, the Adult Pre-exercise Screening Tool (stage 1) will be administered. The tool was developed by Exercise and Sports Science Australia (ESSA), Fitness Australia and the Sports Medicine Australia (SMA) to identify those individuals with a known disease, or signs or symptoms of a disease, who may be at a higher risk of an adverse event during physical activity/exercise. There are seven items listed on the questionnaire. Individuals who answer yes to any of the seven questions will be referred to their GP for safety for exercise clearance before they can be enrolled in our study.

This approach of screening patients for exercise participation is in line with the recommendation from the American College of Sports Medicine. In particular, it will help identify individuals at risk of exercise-related sudden cardiac death and/or acute myocardial infarction while also avoiding excessive physician referrals which create barriers to exercise participation.

Randomisation and blinding:
Allocation of participants in a 1:1 ratio to either a 12-week *yoga program* or *strengthening exercise program* will be based on computer-generated random numbers prepared by a statistician with no involvement in the trial. Code-break for the full randomisation schedule will be maintained by the administering institute.

The assessors will be blinded to treatment allocation for objective outcome measures. Allocation concealment and blinding will be ensured by having two research nurses coordinate the trial. Assessments will be taken by the research nurse blinded to the group allocation.

Treatments:
Overall, 126 eligible participants will be randomly allocated to a 24-week group-based *yoga program* or *strengthening exercise program* (each with two 1-hour group-based sessions and one 1 hour home based session per week). Group sessions will be led by physiotherapist/exercise physiologists/yoga teachers for the first 12 weeks. Trainers will lead a group of approximately 10 participants. Participants will be instructed to continue their *yoga* or *strengthening exercise* at home for another 12 weeks. Using REDCap, weekly email follow-up will be done with the participants to confirm the compliance to the home-based program, and video instructions will be given to each participant to support the home-based program.

An evidence-based *yoga program* designed by a yoga expert and researcher - Dr Steffany Moonaz from the Maryland University of Integrative Health, USA will be delivered by yoga

teachers. Each yoga class will begin with a breathing exercise (*pranayama*) and chanting (10min), warm-up, and moving sequences (asanas, 40 min). Classes will end with deep relaxation (*savasana*) and meditation (10 min). Classes will include an application of yoga philosophy and replace negative thoughts with positive ones (*pratipaksa bhavanam*). Details about interventions are in the appendix.

The strengthening exercise therapy regimen will be designed by Prof Kim Bennell as a group-based progressive strengthening exercise program based on those used in past clinical trials and shown to be effective. Details about interventions are in the appendix.

Safety:
The participants will be asked to come to Menzies centre for the face-to-face group-based *yoga program* or *strengthening exercise program* wherever possible; however, in the event of participants not being unable to come to Menzies centre (due to COVID-19 related restrictions/lockdown) the *yoga program*/*strengthening exercise* classes will be run on zoom with the same teacher.

First 12 weeks of intervention in both groups will be via face-to-face classes and via zoom sessions if there is a lockdown due to Covid restrictions. The participants will be given a detailed information sheet on their yoga/ exercise sessions which they were doing in the first 12 weeks to continue in the next 12 weeks. We will give them short video of the individual sessions for reference.

Considering the COVID-19 situation, we have put the precautions below in place that will be observed during the trial.

1. The participants will enter the Menzies Institute for Medical Research facility after the routine temperature screening.
2. The group-based session will be performed in the rooms with designated COVID-19 capacity to accommodate the group of patients.
3. Patients will receive separate the yoga/exercise mat and elastic band that will not be shared with other participants.

Adverse events will be recorded throughout the study, and CIs will be notified of any serious events within 24 hours.

Adverse events will be monitored throughout the study. Yoga or exercise teachers and the unblinded research assistant will monitor AEs. Adverse events will be defined as any treatment-related problem that lasts for >2 days and/or caused the patient to seek other treatment. Potential AEs from this study include increased knee pain or pain at other sites, temporary muscle stiffness, falls or other injuries related to the exercise. There is a very rare chance of an exercise-induced heart attack or sudden death. AEs in our knee OA exercise trials have mostly been mild and transient and generally comprise of increased knee pain or pain at other sites, which has resolved by individualising the program to the participant's capabilities.

The teachers leading the yoga/strengthening exercise classes will ensure the exercises are performed safely. It is usual to feel some pain/discomfort in the knee during the exercise but the amount of pain will be monitored. When levels of pain are deemed unacceptable by the participant and/or teacher, the exercise program will be modified accordingly. Participants will use similar principles in the unsupervised home sessions. In particular, they will be advised that if the pain has not subsided to usual levels by the next day or there has been increased night pain or swelling in the knee, the exercise program should be modified by reducing the

intensity or frequency of the exercises or by leaving out one or more exercises that may be contributing to the increase in symptoms.

Analgesic use:
To maintain the pragmatic nature of the study, there will be no constraints with regard to the use of analgesic medications. All participants will be allowed to continue taking the medications that they are taking at their screening visit for the duration of the trial. Participants will be asked to keep medications as stable as possible, but if a participant requires an increase in analgesics this will be permitted, and the reason for the dose increase and the dose used will be documented.

Outcomes and assessments:
There will be four study clinic visits (screening, baseline/week 0, week 12, week 24) for objective assessments such as core physical function measures, muscle strength, neuromuscular variables, and functional balance. Pain and function will be assessed at each time point (week 0, 4, 8,12,16, 20, and 24) using a 100mm visual analogue scale (VAS) and asked: "On this line, how would you rate your symptoms in the last week"? We will also assess symptoms using the WOMAC scale. We will include other questionnaires on psychological, QoL (AQoL and EQ-5D), as well as adherence to therapy and healthcare utilisation of the treatment for knee OA.

Primary outcome:
Change in knee pain assessed by VAS for over 12 weeks. Pain will be evaluated at each time point using a 100mm VAS and asked: "On this line, how would you rate your overall average knee pain in the last one week"? For knee pain assessed using VAS, terminal descriptors will be 0mm = no pain, 100mm = worst pain possible.

Secondary outcomes: (overall change from baseline to week 12 and overall change from baseline to week 24 are separate outcomes)

1. Change in VAS knee pain over 12 weeks in patients with painDETECT>12
2. Change in VAS knee pain over 24 weeks.
3. Change in WOMAC knee pain, WOMAC stiffness, and WOMAC knee function over 12 weeks and over 24 weeks.
4. Change in physical function as assessed by 30s Chair Stand Test, 40m Fast Walk Test and Stair Climb Test over 12 and over 24 weeks
5. OMERACT_OARSI responders (meeting responder criteria) at week 12, and 24 weeks
6. Change in biomarker (urinary CTX-II, serum COMP, and serum hyaluronan) and systemic inflammatory markers (hs-CRP, IL-6, TNF-a) over 12, and over 24 weeks
7. Change in Patient Health Questionnaire (PHQ-9) scales over 12, and over 24 weeks
8. Change in patient global satisfaction score over 12, and over 24 weeks
9. Change in painDETECT scores over 12, and over 24 weeks
10. Change in leg muscle strength (quadriceps) over 12, and over 24 weeks
11. Change in gait speed from baseline to week 12 and baseline to 24 weeks

Health Economics Outcomes (secondary outcomes):
We will assess health state utility (HSU) values, resource utilisation, and costs throughout the study. Health-related quality of life (HRQoL) and utility will be assessed using the Assessment of Quality of Life (AQoL-8D) and the EuroQol 5 dimensions (EQ-5D-5L) instruments. The health economic outcomes to be collected as the secondary outcome will include:

12. Change in concomitant medications (assessed using self-reported medication history questionnaire) from baseline to 12 and baseline to 24 weeks and associated costs.

13. Self-reported adherence to the intervention and comparator program from baseline to 12 and baseline to 24 weeks assessed using online logbook and defined as the percentage of prescribed session undertaken.
14. Change in QoL (assessed by AQoL-8D and EQ-5D-5L) from baseline to week 12 and baseline to 24 weeks.
15. Change in health resource utilisation (assessed using a self-reported questionnaire) at 12 and 24 weeks.

Other outcomes:

16. Change in gait characteristics such as step length, double support time, step width, and step time from baseline to week 12 and baseline to 24 weeks.
17. Change in body fat will be assessed using bioelectrical impedance analysis (BIA) (BIA analyser, Quantum II, RJL Systems, Michigan, USA) at baseline, week 12, week 24. We will assess fat-free mass, percentage fat-free mass, fat mass and percentage fat mass.

The health economic measures to be collected at baseline, 12-week, and week 24 include: health service use including visits to GPs, specialist doctors, physiotherapists; medical imaging, procedures, tests and investigations for knee pain; hospital admissions and attendances for knee pain, and community service use for knee pain. We will also collect information on costs for transport and specialised equipment purchased/hired due to knee pain. We will collect information about employment, days absent from work, presenteeism, and sick leave due to knee pain. To assist with cost estimation, we will collect information about participants' concession, health care card & private health insurance status.

In combination with the clinical data, these data will be used to conduct a cost-effectiveness analysis of *yoga program* versus *strengthening exercise program*. We will test that hypothesis that *yoga program* will be cost-effective than the *strengthening exercise program*.

Demographics and Medicare Number

At the screening, we will collect information on sex and date of birth. At baseline, we will record participants' Medicare numbers for future data linkage.

The OARSI-OMERACT responder criteria: will be employed to generate a responder categorical variable (0 = non-responder, 1 = responder) based on improvement in WOMAC pain, function, and patient's global assessment. Patients' global assessment was evaluated using a 100 mm VAS.

Core physical function measures: including 30–second chair stand test, 40m (10m × 4) fast-paced walk test and stair climb test, will be performed as recommended by the OARSI guidelines for clinical trials at baseline and week 12 using the same equipment and at the same location. The total number of chair stand in 30 seconds, time to complete 40 m walk with three turns and time to ascend and descend a 9-step stair (20cm step height and handrail) will be recorded.

PHQ-9:

Depression may be an effect modifier. It will be assessed using the Patient Health Questionnaire (PHQ-9) at weeks 0, 12, and 24. The PHQ-9 is a validated tool for screening, diagnosing, monitoring, and measuring the severity of depression. It consists of nine questions, based on the Diagnostic and Statistical Manual of mental disorders (DSM-IV) criteria for the diagnosis of major depressive disorders in patients with medical illnesses.

Procedure regarding the identification and management of moderate to severe depressive symptoms in the PHQ-9 scale:

A score of 15 or more (out of 27) on the PHQ-9 is provisionally diagnostic of moderate to severe major depression. Furthermore, question 9 assesses suicide ideation. Any positive response (i.e. "several days", "more than half the days", or "nearly every day") warrants immediate management.

As part of a duty of care, PHQ-9 scores will be assessed immediately by a member of the research team at the study visit [baseline, 12 weeks (3 months), 24 weeks (6 months)]. It will be screened for scores of 15 or more, or any positive response to question 9. Any participants with scores 15 or more or with positive responses for suicide ideation will be encouraged to contact their GP. We will also provide these participants with phone numbers for support services, including Lifeline and Beyond Blue. Any positive responses and actions taken will be logged and maintained on a study register.

Gait characteristics
Gait will be assessed using the footfalls recorded on the GAITRite system, a 4.6 m computerized walkway. Measures gait characteristics such as gait speed (cm/s), step length (cm), double support time (ms), step width (cm), and step time (ms) will be directly obtained from the GAITRite software.

Physical activity (using accelerometers)
We will ask the participants to wear accelerometers for a week before the start of intervention, 12 weeks and 24 weeks. We will assess the change in objectively measured physical activity between the groups.

Change in medication use
All participants will be asked to continue therapy with the medications they were receiving at their screening visit for the duration of the trial. They will also be asked to keep their medication use as stable as possible; any medication changes were documented with the reason, drug name, and dose and were classified as commenced or increased, discontinued or decreased, or stable use or nonuse. A rescue medication, paracetamol, will be provided if the participant requested it. Medication use will be recorded at baseline and each follow-up period.

Data integrity and management
We will collect all data using REDCap, a secure web application for managing databases. Paper copies of participant questionnaires will be stored in locked filing cabinets, with restricted access. Electronic data will be kept on password-protected servers, separating the identifying and non-identifying information. The codes linking data to identifying participant information will be kept separately from the study data, under password protection, and with restricted access. Only members of the study team who need to contact trial participants, enter data, or perform data quality control will have access to identifiable information. Daily backups of all electronic data will occur to minimize any risk of lost data.
Blood results will be identifiable. Only our research staff and pathology staff will have access to these, and they will be stored in the same manner as outlined above.
After the completion of trial, paper copies of data will be archived in secure storage. Any identifiers will not be removed, in case of follow-up of study participants being necessary, but the electronic data will continue to be maintained in a secure electronic database, separating identifying and non-identifying information. This will remain password-protected and with access given only to the investigators unless otherwise authorized by the investigators.

Items/Variables	Screening	Baseline (week 0)	Week 4	Week 8	Week 12	Week 16	Week 20	Week 24
Informed consent	x							
Randomisation		x						
Safety for exercise clearance	x							
ACR clinical criteria for knee OA	x							
Medicare number		x						
**Clinical measures**								
Bloods (stored for cartilage/ synovium/ inflammatory markers)		x			x			x
Core physical function tests		x			x			x
Leg muscle strength test		x			x			x
Height and weight		x			x			x
Gait characteristics		x			x			x
Body composition (using BIA)		x			x			x
Physical activity (using accelerometers)		x						x
**Questionnaires**								
Knee pain VAS	x	x	x	x	x	x	x	x
Knee WOMAC		x	x	x	x	x	x	x
PainDETECT		x			x			x
PHQ-9		x			x			x
Patient global evaluation		x	x	x	x	x	x	x
Health Economics Outcomes:<br>*Medication cost diary*<br>*Health service utilization (visit to GP, practice nurses, and any other health professionals (e.g. physiotherapists))*<br>*Employment/Days off work*<br>*Concession/Health Care Card*<br>*Private Health Insurance*<br>*Transport & Specialised Equipment Costs*		x			x			x

Safety (AEs)			x	x	x	x	x	x
EQ-5D and AQoL-8D		x			x			x
Consent to contact for future studies								x
Early withdrawal information		As required						

Sample Size calculation:
Our power calculations are based on a sample size that can document the minimal clinically important difference (MCID) in VAS knee pain superior to yoga compared to strengthening exercise. Based on the MCID of 15mm in VAS pain [a numerical scale ranging from 0 (no problem) to 100 (maximum problem)], two-sided significance level of 0.05, 90% power and SD of VAS pain change 22.5, we will need a total of 98 participants. Assuming a worse scenario of 20% dropout, we will need 126 participants.

The above sample size will allow us to detect a non-inferiority of yoga with strengthening exercise. Non-inferiority would be declared if the mean change in knee pain (assessed using VAS) in the yoga group was not significantly worse than the mean change in the strengthening exercise group, within a pre-stated margin of non-inferiority (Δ), in this case, set at 10 mm. Overall, 126 participants will give us 80% power to detect the non-inferiority of yoga compared with strengthening exercise.

Statistical Analyses:
The comparisons of pain and other continuous scores will be made using a repeated–measures mixed model with terms of treatment, time, and corresponding baseline values as covariates. The effect of treatment will be evaluated by the intervention by time interaction, and then proceed to the main effects model with only group and time. We will also assess the effects of potential confounders or interaction with treatment by covariates, including age, sex, BMI, disease severity, comorbidities, health status, and use of pain medications. A 2-sided P-value less than 0.05 will be considered to indicate the statistical significance, and the results will be shown as the between-group differences with 95% confidence intervals (95% CIs) of the differences.

We will explore the role of clustering effects in the trial by performing a sensitivity analysis examining the implications of clustering by the instructor by adding an instructor random effect to the mixed-effects model.

Cost-effectiveness analysis:
We will perform the cost-effectiveness analysis (CEA) by measuring the costs and benefits in the *yoga program* group and the *strengthening exercise program group*. Mean differences in total costs and benefits between the *yoga program* and *strengthening exercise program* at 12 weeks and 24 weeks follow-up period will be calculated. Incremental cost-effectiveness ratios (ICERs) will be determined by dividing the difference in total costs by the difference in total benefits for both groups (equation below). We will also conduct the subgroup analysis based on the degree of knee pain at baseline, the adherence to the *yoga program*, gender, socioeconomic status.

Measurement of costs
The health questionnaires completed by participants at baseline, 12-weeks, and 24-months will provide data on “health service use”. This data will include visits to GPs, practice nurses, and any other health professionals (e.g. physiotherapists) for the treatment and/or management of knee OA. To estimate total costs, we will assign unit costs for each visit to a health care professional. Unit costs will be obtained from national published sources such as the appropriate Australian Annual Medicare Statistics providing health service unit cost and the Indexation of Medicare Benefits Schedule (48).

Measurement of benefit

The AQoL-8D and EQ-5D-5L will be used to determine HSU for each participant. Mean scores and measures of dispersion will be calculated for both groups. Quality-adjusted life-years (QALYs) will be calculated using two approaches: change from baseline (CfB), and area under the curve (AUC) approach with/without linear regression (49, 50).

Uncertainty and sensitivity analysis
A summary measure of the uncertainty of costs and effects will be presented using cost-effectiveness acceptability curves (CEAC). The CEAC will show a range of probabilities of an intervention being cost-effectiveness at different ceiling thresholds (i.e. a maximum amount that decision-makers are willing to pay for a unit of benefit). To test the robustness of the results, a series of sensitivity analyses will be conducted to explore the variability in estimating cost-effectiveness.

$$ICER = \frac{mean\ cost\ yoga\ program\ group\ - mean\ cost\ physical\ therapy\ group}{mean\ QALY\ yoga\ program\ group - mean\ QALY\ physical\ therapy\ group}$$

Timelines:

Milestones	2020				2021				2022			
Ethics and study set–up	X	X	X	X	X							
Recruitment, screening, and initiation of intervention						X	X	X				
24 weeks visit for final outcomes								X	X	X		
Data cleaning, analysis and write up										X	X	X

Significance:
While it is established that the physical component of yoga provides the exercise that is consistent with recommendations for knee OA, the mental component has the potential to increase psychological well-being reducing stress, which can influence the central pain processing and alleviate the pain symptoms. If yoga can improve the pain and function in knee OA, it may slow the progression to joint replacement, thus contributing to cost savings to the health system. The proposed study represents an innovative approach to this and lends itself to easy implementation as yoga is a popular exercise program and can easily be incorporated in the program of management of knee OA.

References:

1. Deveza LA, Melo L, Yamato TP, Mills K, Ravi V, Hunter DJ. Knee osteoarthritis phenotypes and their relevance for outcomes: a systematic review. Osteoarthritis Cartilage. 2017;25(12):1926-41.
2. Kittelson AJ, Stevens-Lapsley JE, Schmiege SJ. Determination of Pain Phenotypes in Knee Osteoarthritis: A Latent Class Analysis Using Data From the Osteoarthritis Initiative. Arthritis Care Res (Hoboken). 2016;68(5):612-20.
3. Dell'Isola A, Steultjens M. Classification of patients with knee osteoarthritis in clinical phenotypes: Data from the osteoarthritis initiative. PLoS One. 2018;13(1):e0191045.
4. Scopaz KA, Piva SR, Wisniewski S, Fitzgerald GK. Relationships of fear, anxiety, and depression with physical function in patients with knee osteoarthritis. Arch Phys Med Rehabil. 2009;90(11):1866-73.
5. Somers TJ, Keefe FJ, Pells JJ, Dixon KE, Waters SJ, Riordan PA, et al. Pain catastrophizing and pain-related fear in osteoarthritis patients: relationships to pain and disability. J Pain Symptom Manage. 2009;37(5):863-72.
6. Heuts PH, Vlaeyen JW, Roelofs J, de Bie RA, Aretz K, van Weel C, et al. Pain-related fear and daily functioning in patients with osteoarthritis. Pain. 2004;110(1-2):228-35.
7. Arendt-Nielsen L, Nie H, Laursen MB, Laursen BS, Madeleine P, Simonsen OH, et al. Sensitization in patients with painful knee osteoarthritis. Pain. 2010;149(3):573-81.
8. Arendt-Nielsen L, Egsgaard LL, Petersen KK, Eskehave TN, Graven-Nielsen T, Hoeck HC, et al. A mechanism-based pain sensitivity index to characterize knee osteoarthritis patients with different disease stages and pain levels. Eur J Pain. 2015;19(10):1406-17.
9. Finan PH, Buenaver LF, Bounds SC, Hussain S, Park RJ, Haque UJ, et al. Discordance between pain and radiographic severity in knee osteoarthritis: findings from quantitative sensory testing of central sensitization. Arthritis Rheum. 2013;65(2):363-72.
10. Dimitroulas T, Duarte RV, Behura A, Kitas GD, Raphael JH. Neuropathic pain in osteoarthritis: a review of pathophysiological mechanisms and implications for treatment. Semin Arthritis Rheum. 2014;44(2):145-54.
11. Ohtori S, Orita S, Yamashita M, Ishikawa T, Ito T, Shigemura T, et al. Existence of a neuropathic pain component in patients with osteoarthritis of the knee. Yonsei Med J. 2012;53(4):801-5.
12. Polat CS, Dogan A, Sezgin Ozcan D, Koseoglu BF, Kocer Akselim S. Is There a Possible Neuropathic Pain Component in Knee Osteoarthritis? Arch Rheumatol. 2017;32(4):333-8.
13. Thakur M, Dickenson AH, Baron R. Osteoarthritis pain: nociceptive or neuropathic? Nat Rev Rheumatol. 2014;10(6):374-80.
14. Hurley M, Dickson K, Hallett R, Grant R, Hauari H, Walsh N, et al. Exercise interventions and patient beliefs for people with hip, knee or hip and knee osteoarthritis: a mixed methods review. Cochrane Database Syst Rev. 2018;4:Cd010842.
15. Goh SL, Persson MSM, Stocks J, Hou Y, Lin J, Hall MC, et al. Efficacy and potential determinants of exercise therapy in knee and hip osteoarthritis: A systematic review and meta-analysis. Ann Phys Rehabil Med. 2019;62(5):356-65.
16. Wang SY, Olson-Kellogg B, Shamliyan TA, Choi JY, Ramakrishnan R, Kane RL. Physical therapy interventions for knee pain secondary to osteoarthritis: a systematic review. Ann Intern Med. 2012;157(9):632-44.
17. Lluch E, Torres R, Nijs J, Van Oosterwijck J. Evidence for central sensitization in patients with osteoarthritis pain: a systematic literature review. Eur J Pain. 2014;18(10):1367-75.
18. Dobson JL, McMillan J, Li L. Benefits of exercise intervention in reducing neuropathic pain. Front Cell Neurosci. 2014;8:102.
19. Telles S, Sayal N, Nacht C, Chopra A, Patel K, Wnuk A, et al. Yoga: Can it be integrated with treatment of neuropathic pain? Ann Neurosci. 2019;26(2):82-91.
20. Vallath N. Perspectives on yoga inputs in the management of chronic pain. Indian J Palliat Care. 2010;16(1):1-7.
21. McAlindon TE, Bannuru RR, Sullivan MC, Arden NK, Berenbaum F, Bierma-Zeinstra SM, et al. OARSI guidelines for the non-surgical management of knee osteoarthritis. Osteoarthritis Cartilage. 2014;22(3):363-88.
22. Nelson AE, Allen KD, Golightly YM, Goode AP, Jordan JM. A systematic review of recommendations and guidelines for the management of osteoarthritis: The chronic osteoarthritis management initiative of the U.S. bone and joint initiative. Semin Arthritis Rheum. 2014;43(6):701-12.
23. Maddux RE, Daukantaite D, Tellhed U. The effects of yoga on stress and psychological health among employees: an 8- and 16-week intervention study. Anxiety Stress Coping. 2018;31(2):121-34.
24. de Manincor M, Bensoussan A, Smith C, Fahey P, Bourchier S. Establishing key components of yoga interventions for reducing depression and anxiety, and improving well-being: a Delphi method study. BMC Complement Altern Med. 2015;15:85-.
25. Kan L, Zhang J, Yang Y, Wang P. The Effects of Yoga on Pain, Mobility, and Quality of Life in Patients with Knee Osteoarthritis: A Systematic Review. Evid Based Complement Alternat Med. 2016;2016:6016532.

26. Welfare AIoHa. Osteoarthritis Canberra: AIHW; 2019 [Available from: https://www.aihw.gov.au/reports/chronic-musculoskeletal-conditions/osteoarthritis.
27. (AOANJRR) AOANJRR. Hip, Knee & Shoulder Arthroplasty: 2019 Annual Report Adelaide: AOANJRR; 2019 [Available from: https://aoanjrr.sahmri.com/documents/10180/668596/Hip%2C+Knee+%26+Shoulder+Arthroplasty/c287d2a3-22df-a3bb-37a2-91e6c00bfcf0.
28. DeRogatis M, Anis HK, Sodhi N, Ehiorobo JO, Chughtai M, Bhave A, et al. Non-operative treatment options for knee osteoarthritis. Ann Transl Med. 2019;7(Suppl 7):S245.
29. Wylde V, Hewlett S, Learmonth ID, Dieppe P. Persistent pain after joint replacement: prevalence, sensory qualities, and postoperative determinants. Pain. 2011;152(3):566-72.
30. Thakur M, Rahman W, Hobbs C, Dickenson AH, Bennett DL. Characterisation of a peripheral neuropathic component of the rat monoiodoacetate model of osteoarthritis. PLoS One. 2012;7(3):e33730.
31. Rahman W, Bauer CS, Bannister K, Vonsy JL, Dolphin AC, Dickenson AH. Descending serotonergic facilitation and the antinociceptive effects of pregabalin in a rat model of osteoarthritic pain. Mol Pain. 2009;5:45.
32. Taibi DM, Vitiello MV. A pilot study of gentle yoga for sleep disturbance in women with osteoarthritis. Sleep Med. 2011;12(5):512-7.
33. Cramer H, Ward L, Steel A, Lauche R, Dobos G, Zhang Y. Prevalence, Patterns, and Predictors of Yoga Use: Results of a U.S. Nationally Representative Survey. Am J Prev Med. 2016;50(2):230-5.
34. Clarke TC, Black LI, Stussman BJ, Barnes PM, Nahin RL. Trends in the use of complementary health approaches among adults: United States, 2002-2012. Natl Health Stat Report. 2015(79):1-16.
35. Barnes PM, Bloom B, Nahin RL. Complementary and alternative medicine use among adults and children: United States, 2007. Natl Health Stat Report. 2008(12):1-23.
36. Cheung C, Park J, Wyman JF. Effects of Yoga on Symptoms, Physical Function, and Psychosocial Outcomes in Adults with Osteoarthritis: A Focused Review. Am J Phys Med Rehabil. 2016;95(2):139-51.
37. Juhl C, Christensen R, Roos EM, Zhang W, Lund H. Impact of exercise type and dose on pain and disability in knee osteoarthritis: a systematic review and meta-regression analysis of randomized controlled trials. Arthritis Rheumatol. 2014;66(3):622-36.
38. Patel NK, Newstead AH, Ferrer RL. The effects of yoga on physical functioning and health related quality of life in older adults: a systematic review and meta-analysis. J Altern Complement Med. 2012;18(10):902-17.
39. Zhang W, Moskowitz RW, Nuki G, Abramson S, Altman RD, Arden N, et al. OARSI recommendations for the management of hip and knee osteoarthritis, Part II: OARSI evidence-based, expert consensus guidelines. Osteoarthritis Cartilage. 2008;16(2):137-62.
40. Bernstein S. Yoga Benefits for Arthritis: Arthritis Foundation; 2020 [Available from: https://www.arthritis.org/health-wellness/healthy-living/physical-activity/yoga/yoga-benefits-for-arthritis.
41. Practitioners TRACoG. Guideline for the management of knee and hip osteoarthritis: The Royal Australian College of General Practitioners Ltd; 2018 [Available from: https://www.racgp.org.au/download/Documents/Guidelines/Musculoskeletal/guideline-for-the-management-of-knee-and-hip-oa-2nd-edition.pdf.
42. Park J, McCaffrey R, Dunn D, Goodman R. Managing osteoarthritis: comparisons of chair yoga, Reiki, and education (pilot study). Holist Nurs Pract. 2011;25(6):316-26.
43. Zhang Q, Young L, Li F. Network Meta-Analysis of Various Nonpharmacological Interventions on Pain Relief in Older Adults With Osteoarthritis. Am J Phys Med Rehabil. 2019;98(6):469-78.
44. Lauche R, Hunter DJ, Adams J, Cramer H. Yoga for Osteoarthritis: a Systematic Review and Meta-analysis. Curr Rheumatol Rep. 2019;21(9):47.
45. Hahn OM, Schilsky RL. Randomized controlled trials and comparative effectiveness research. J Clin Oncol. 2012;30(34):4194-201.
46. Luce BR, Kramer JM, Goodman SN, Connor JT, Tunis S, Whicher D, et al. Rethinking randomized clinical trials for comparative effectiveness research: the need for transformational change. Ann Intern Med. 2009;151(3):206-9.
47. Gierisch JM, Myers ER, Schmit KM, McCrory DC, Coeytaux RR, Crowley MJ, et al. Prioritization of patient-centered comparative effectiveness research for osteoarthritis. Ann Intern Med. 2014;160(12):836-41.
48. Online M. Indexation of Medicare Benefits Schedule (MBS) items from 1 July 2018: MBS; [Available from: http://www.mbsonline.gov.au/internet/mbsonline/publishing.nsf/Content/MBSIndexation-July2018.
49. Horsman J, Furlong W, Feeny D, Torrance G. The Health Utilities Index (HUI): concepts, measurement properties and applications. Health Qual Life Outcomes. 2003;1:54.
50. Manca A, Hawkins N, Sculpher MJ. Estimating mean QALYs in trial-based cost-effectiveness analysis: the importance of controlling for baseline utility. Health Econ. 2005;14(5):487-96.

SUMMARY OF PROTOCOL CHANGES

Protocol Version number 1.6, dated 12 Sep 2022

1. There were no major deviations from the protocol, except for the rescheduling of clinic visits due to COVID-19 restrictions.
2. We acknowledge that additional outcome measures outlined in trial protocol, such as cost-effectiveness (health economics) outcomes, physical activity, gait characteristics, and body fat, are not presented in this manuscript. As the analysis of these data is still ongoing, they have not been included. We remain committed to thoroughly exploring and reporting these outcomes in separate publications.
3. We have collected the serum and urine samples and stored them for analyses. However, we are yet to do the biochemical markers proposed in the protocol and registry due to the lack of funding.

Study Protocol: A randomised comparative effectiveness trial of YOGa and strengthening exercise for knee osteoArthritis (YOGA Trial)

Version number 1.6, dated 12 Sep 2022

Trial registration: anzctr.org.au Identifier: ACTRN12621000066886.

Principle investigator

Dr Benny Antony[1], Senior Research Fellow

Chief investigators

Dr Steffany Moonaz[2], Director of Clinical and Academic Research

Prof Kim Bennell[3], Director of the Centre for Health, Exercise and Sports Medicine

Prof Graeme Jones[1], Rheumatologist and Professorial Research Fellow

Assoc Prof Dawn Aitkens[1], Research Fellow and Head of Menzies Musculoskeletal Unit

Prof Andrew Palmer[1], Head of the Menzies Health Economics Unit

Prof Leigh Blizzard[1], Head of the Menzies Statistical Unit

Affiliations

[1]Menzies Institute for Medical Research, University of Tasmania

[2]Maryland University of Integrative Health, USA

[3]Department of Physiotherapy, School of Health Sciences, Faculty of Medicine, Dentistry and Health Sciences, University of Melbourne

Funding

This trial is funded by the Rebecca L Cooper Medical Research Foundation.

STATISTICAL ANALYSIS PLAN (SAP)

There was no separate Statistical analysis plan prepared for this study apart from what was entered into the protocol. These parts of statistical analysis are taken from the protocol, which are available in these documents.

SAP as described in the approved version of protocol.

Statistical consideration

Hypothesis, primary endpoints (i.e., primary outcome variables), and secondary endpoints

Research hypothesis:

Primary alternative hypothesis: Yoga program will be superior to strengthening exercise program on reducing knee pain (assessed using VAS) over 12 weeks in knee OA patients.

Secondary alternative hypothesis: Yoga program will improve secondary outcomes (namely VAS knee pain over 24 weeks, WOMAC pain, WOMAC function, WOMAC stiffness, OMERACT-OARSI responders, patient global assessment, painDETECT score, Depression (assessed by patient health questionnaire), quality of life (AQol-8D and EQ-5D-5L), leg muscle strength, core physical performance measures (30-s chair stand test, 40-m fast-paced walk and stair climbs test), and self-reported adherence compared to strengthening exercise.

Primary outcome measure:

Change in overall average knee pain assessed by visual analogue scale (VAS) over 12 weeks.

Pain will be evaluated at each time point using a 100 mm VAS and asked: "On this line, how would you rate your overall average knee pain in the last one week?" For knee pain assessed using VAS, terminal descriptors will be 0 mm = no pain to100 mm = worst pain possible.

Secondary outcome measures:

1. Change in VAS knee pain over 24 weeks.
2. Change in WOMAC pain, WOMAC function, and WOMAC stiffness over 12 weeks and over 24 weeks.
3. Change in VAS knee pain over 12 weeks in patients with painDETECT>12
4. Change in core physical performance measures as assessed by 30 s chair stand test, 40 m fast walk test, and stair climb test over 12 and over 24 weeks.
5. Change in Patient Health Questionnaire (PHQ-9) scales over 12 and over 24 weeks.
6. Change in patient global assessment score (assessed using a 100 mm VAS) over 12 and over 24 weeks.
7. Change in neuropathic pain, as assessed by the painDETECT questionnaire, over 12 and over 24 weeks.
8. Change in leg muscle strength will be assessed by leg muscle strength dynamometry at the lower limb (involving both legs simultaneously) over 12 and over 24 weeks.
9. Self-reported adherence to the yoga or strengthening exercise program from baseline to 12 and baseline to 24 weeks will be assessed using an online logbook and defined as the percentage of prescribed sessions undertaken.
10. The OARSI-OMERACT responder criteria: This will be employed to generate a responder categorical variable (0 = non-responder, 1 = responder) based on improvement in WOMAC pain, function, and patient global assessment.
11. Change in QoL (assessed by AQoL-8D and EQ-5D-5L) from baseline to week 12 and baseline to 24 weeks.
12. Change in concomitant pain medications.

Data analysis:

The comparisons of pain and other continuous outcomes will be done employing a repeated measures mixed model incorporating terms of treatment, time, and respective baseline values as covariates. We will also assess the effects of potential confounders or interaction with treatment by covariates, including age, sex, BMI, disease severity, comorbidities, health status, and use of pain medications. A two-sided p-value less than 0.05 will be considered to indicate the statistical significance, and the results will be shown as the between-group differences with 95% confidence intervals (95% CIs) of the differences.

We will explore the role of clustering effects in the trial by performing a sensitivity analysis examining the implications of clustering by the instructor by adding an instructor random effect to our mixed-effects model.

We will also perform an exploratory analysis to investigate whether the presence or absence of neuropathic pain, as assessed by the painDETECT questionnaire, is a potential moderator that influences response to treatment for the primary outcome at 12 weeks. To assess the moderation of the effect by painDETECT (binary moderator), an interaction term between the randomised group and the potential moderator will be included in outcome regression models.

Sample size calculation.

Our power calculations are based on a sample size that can document the superiority of yoga compared to strengthening exercise by an amount that meets the minimal clinically important difference (MCID). Based on the MCID of 15 mm in VAS pain (a numerical scale ranging from 0 (no problem) to 100 (maximum problem)), the two-sided significance level of 0.05, 90% power, and SD of VAS pain change 22.5, we will need a total of 98 participants.

Assuming a scenario of around 20% dropout, we will need 126 participants. The above sample size will allow us to detect a non-inferiority of yoga with strengthening exercise. Non-inferiority would be declared if the mean change in knee pain (assessed using VAS) in the yoga group was not significantly worse than the mean change in the strengthening exercise group, within a pre-stated margin of non-inferiority (D), in this case, set at 10 mm. Overall, 126 participants will give us 80% power to detect the non-inferiority of yoga compared with strengthening exercise.

Statistical analysis used in final manuscript.

1. Statistical methods (details of primary analyses)

Analyses were performed using Stata version 18 (Stata Corporation, Inc., College Station, TX, USA) and a two-sided P value of 0.05 was deemed statistically significant.

Normality was assessed by examining the distribution of residuals, and homogeneity of variance was evaluated through visual inspection of plots of residuals versus fitted values.

1. Linear Mixed Effects Model (Repeated-measures mixed-effects model)

We chose mixed-effects models for the primary analyses because they are well-suited to handle longitudinal data with repeated measures over time. These models account for the correlation within subjects by including random effects, which allows each participant to serve as their own control and reduces bias arising from individual differences. Moreover, mixed-effects models are flexible in accommodating missing data under the assumption that data are missing at random. Importantly, no imputation procedures for missing data were performed, as these were not pre-specified in the original study protocol.

STATA functions used: -mixed function in STATA (version 18) was used for the modelling of linear mixed effects model. The -margins command was used to calculate the estimated value of the outcome measure at each time point. The -lincom function was used to calculate the within- and between-group differences over time.

The outcomes for which we used linear mixed effects models were visual analog scale (VAS) knee pain, Western Ontario and McMaster University Index (WOMAC) subscales (pain, function, and stiffness), patient global assessment, Depression (as assessed by patient health questionaries), quality of life derived utility values, core physical performance measures (as assessed by 30 s chair stand test, 40 m fast walk test, and stair climb test), neuropathic pain (as assessed by painDETECT questionnaire) and leg muscle strength. First, the main effects are treatment group, time, and interaction of treatment group with time. The presence of time interaction allows us to estimate the treatment effect at different time points (each follow-up). Second, the model was also adjusted for baseline values for each continuous outcome, age, sex, and BMI. Furthermore, the interaction terms for each confounder with time were added. Third, participant ID was included as a random effect allowing random intercepts. This addresses the correlation within repeated measures. Fourth, the mixed-effects model was fitted using default setting of -mixed function (independent covariance and the restricted maximum likelihood method).

2. Binomial regression

For the binomial regression, -glm function was used to estimate the risk ratio or risk difference by specifying the options family () and link (). Treatment group was included as the univariate predictor of OARSI-OMERACT responders.

- risk ratio by -glm with family (binomial) and link (log),
- risk difference by -glm with family (binomial) and link (identity).

SAP-summary of changes

Few changes were made, and while not updated in the original protocol, published protocol version and registry.

1. **Original Protocol**: We stated we will explore the role of clustering effects in the trial by performing a sensitivity analysis examining the implications of clustering by the instructor by adding an instructor random effect to our mixed-effects model.

   ANZCTR – Registration: We will explore the role of clustering effects in the trial by performing a sensitivity analysis examining the implications of clustering by the instructor by adding an instructor random effect to our mixed-effects model.

   Open access protocol publication: We will explore the role of clustering effects in the trial by performing a sensitivity analysis examining the implications of clustering by the instructor by adding an instructor random effect to our mixed-effects model.

   Final Manuscript: We did not examine the role of clustering effects in the trial because more than 80% of the yoga classes were conducted by one instructor, and more than 80% of the strengthening exercise classes were conducted by another instructor.

2. **Original Protocol**: We stated we will assess the effects of potential confounders or interaction with treatment by covariates, including age, sex, BMI, disease severity, comorbidities, health status, and use of pain medications.
   ANZCTR – Registration: We stated we will also assess the effects of potential confounders or interaction with treatment by covariates, including age, sex, BMI, disease severity, comorbidities, health status, and use of pain medications.
   Open access protocol publication: We stated we will assess the effects of potential confounders or interaction with treatment by covariates, including age, sex, BMI, disease severity, comorbidities, health status, and use of pain medications.
   Final Manuscript: Regarding the additional adjustments for disease severity that was initially specified in our study protocol, we were unable to include these variables in our final models due to inadequate data. However, we adjusted our model for comorbidities, health status, and use of pain medications, and the results were similar to those of the unadjusted model, so we removed it from the model.
3. **Original Protocol**: We stated "the above sample size will allow us to detect a non-inferiority of yoga with strengthening exercise. Non-inferiority would be declared if the mean change in knee pain (assessed using VAS) in the yoga group was not significantly worse than the mean change in the strengthening exercise group, within a pre-stated margin of non-inferiority (Δ), in this case, set at 10 mm".

   Open access protocol publication: We stated "the above sample size will allow us to detect a non-inferiority of yoga with strengthening exercise. Non-inferiority would be declared if the mean change in knee pain (assessed using VAS) in the yoga group was not significantly worse than the mean change in the strengthening exercise group, within a pre-stated margin of non-inferiority (Δ), in this case, set at 10 mm".

   Final Manuscript: Since our study was powered for non-inferiority and has met the criteria for switching the trial objective from superiority to non-inferiority, as outlined by the European Agency for the Evaluation of Medicinal Products (EMEA 2001), we conducted a non-inferiority analysis.
