# Supplementary material for: Yoga or Strengthening Exercise for Knee Osteoarthritis: A Randomized Clinical Trial
Source: JAMA Netw Open. 2025 Apr 8;8(4):e253698. doi: 10.1001/jamanetworkopen.2025.3698 (PMC11979726; doi:10.1001/jamanetworkopen.2025.3698)
Supplement: Supplement 2. — eMethods. Additional details eResults. A priori subgroup analyses eTable 1. Sensitivity analyses based on the per-protocol population on change in study endpoints between the yoga and strengthening groups over 12- and 24-weeks eTable 2. Change in knee symptoms (VAS & WOMAC) from baseline to all time points eTable 3. Change in knee symptoms (VAS & WOMAC) from baseline to all time points in participants with possible neuropathic pain and without neuropathic pain at baseline eTable 4. Change in pain medication and supplements over 12- and 24-weeks eTable 5. Adherence measures over the first 12 and 24 weeks for participants in yoga and strengthening groups eTable 6. Participants' fidelity to intervention protocol across groups, reported as n (%) unless otherwise indicated eFigure 1. Study schedule of the trial eFigure 2. Mean VAS knee pain and WOMAC scores (95% CI) in the yoga and strengthening groups over 12 weeks eFigure 3. Change in VAS and WOMAC scores over 12 and 24 weeks, stratified by baseline PainDETECT score, for the yoga and strengthening groups eFigure 4. Non-inferiority analysis of VAS knee pain score at 12 weeks eAppendix. Yoga and strengthening exercises: intervention description/manual [file jamanetwopen-e253698-s002.pdf]

Supplementary Online Content

Abafita BJ, Singh A, Aitken D, et al. Comparative effectiveness of yoga and strengthening exercise for treating knee osteoarthritis: a randomized clinical trial. *JAMA Netw Open*. 2025;8(4):e253698. doi:10.1001/jamanetworkopen.2025.3698

eMethods. Additional details

eResults. A priori subgroup analyses

eTable 1. Sensitivity analyses based on the per-protocol population on change in study endpoints between the yoga and strengthening groups over 12- and 24-weeks

eTable 2. Change in knee symptoms (VAS & WOMAC) from baseline to all timepoints

eTable 3. Change in knee symptoms (VAS & WOMAC) from baseline to all timepoints in participants with possible neuropathic pain and without neuropathic pain at baseline

eTable 4. Change in pain medication and supplements over 12- and 24-weeks follow-up by treatment groups

eTable 5. Adherence measures over the first 12 and 24 weeks for participants in yoga and strengthening groups

eTable 6. Participants' fidelity to intervention protocol across groups, reported as n (%) unless otherwise indicated

eFigure 1. Study schedule of the trial

eFigure 2. Mean VAS knee pain and WOMAC subscale scores (95% CI) in the yoga and strengthening groups over 12 weeks

eFigure 3. Change in VAS and WOMAC scores over 12 and 24 weeks, stratified by baseline PainDETECT score, for the yoga and strengthening groups

eFigure 4. Non-inferiority analysis of VAS knee pain score at 12 weeks

eReferences.

eAppendix. Yoga and Strengthening Exercises: Intervention Description/Manual

© 2025 Abafita BJ et al. *JAMA Network Open*.

This supplementary material has been provided by the authors to give readers additional information about their work.

© 2025 Abafita BJ et al. *JAMA Network Open*.

eMethods. Additional Details

Additional Details of Eligibility Criteria

Eligibility criteria are detailed in the published protocol.[1] Participants were eligible to participate in the study if aged 40 years or older with knee pain of at least 40 mm on a 100-mm Visual analog scale (VAS) in the last month and met the ACR clinical criteria for the diagnosis of knee OA. Participants were excluded if they were engaged in a strengthening program or yoga program in the past three months or were planning to start an exercise-based treatment program for knee OA in the next six months. Other exclusion criteria included, a significant knee injury that required treatment within the last six months, arthroscopic or open surgery in the index knee in the last six months or were planning to have such procedure in the next 6-8 months, partial or total knee replacement, received corticosteroids injection (last three months) or hyaluronic acid (last six months) in the index knee, pregnant or breastfeeding, participating in any experimental drug or device or exercise clinical trial related to OA, any condition that precluded the safe participation in exercise as determined by Adult Pre-exercise Screening System (APSS) screening tool,[2] unable to walk without a gait aid, unable to provide informed consent in English and any planned absences of >2 weeks maximum during the first 12 week period.

Additional Description of Study Interventions

The group supervised sessions were conducted at the Menzies Institute for Medical Research, a facility equipped to support the intervention. Unsupervised sessions were home-based and carried out by participants in their own residences. All necessary equipment and accessories for yoga and strengthening exercises were provided to participants to ensure consistency and adherence to the intervention protocol.

Description of the Yoga Program

An evidence-based yoga program was designed based on our prior yoga for arthritis research.[3,4] The yoga program was delivered by Registered Yoga Teachers (RYT)-200 or greater. The yoga instructors received orientation to the intervention, prior training addressing potential questions or concerns, and ongoing support throughout the intervention period as needed. Each yoga session began with breathing exercise (pranayama) and chanting (10 minutes), followed by warm-up and moving sequences (asana, 40 minutes). The classes concluded with deep relaxation (savasana) and meditation (10 minutes). The program incorporated yoga philosophy (yama and niyamas) and the practice of replacing negative thoughts with positive ones (pratipaksa bhavanam).[4] The yoga practices were adapted to accommodate the specific needs and limitations of each participant. Additionally, participants had access to online yoga practices designed for this study, with further details provided in the following section.

Description of Strengthening Exercise Program

An evidence-based lower-limb strengthening exercise therapy program was designed based on our prior OA research.[5] The strengthening exercises were delivered as a progressive group-based program by instructors with a Bachelor's degree in Physiotherapy. The physiotherapists received orientation to the intervention, prior training addressing potential questions or concerns, and ongoing support throughout the intervention period as needed. Each session began with a brief assessment (5 minutes), followed by a warm-up (5 minutes) and lower limb strengthening exercises (run as a circuit class for 45 min), and concluded with a 5-minute cool-down period. Detailed information about this intervention is available in the eAppendix.

Adherence Monitoring

Participants in both the strengthening and yoga programs recorded their Rating of Perceived Exertion (RPE) for each prescribed exercise or yoga session. These records were maintained weekly, except for weeks 12 and 24 when participants attended clinic visits for assessments. Data captured included the number of sessions where RPE values met or exceeded the target range (RPE ≥ 5). This participant-reported measure of exertion provided valuable insights into adherence, exercise intensity, and overall fidelity to the intervention protocol.

Additional details of Secondary Outcomes

Assessment of Pain, function, and stiffness

Knee pain was assessed using a 100-mm VAS (0 mm = no pain to 100 mm = worst pain possible) over 12 and 24 weeks through a standard question (eFigure 1): "On this line, how would you rate your overall average knee pain in the last one week?" WOMAC pain (0 to 500 mm, based on 5 questions), WOMAC physical function (0 to 1700 mm, based on 17 questions), and WOMAC stiffness (0 to 200 mm, based on 2 questions) were also assessed at each time point. Each item was rated on a scale of 0 to 100, with higher scores indicating worse pain, function,

© 2025 Abafita BJ et al. JAMA Network Open.

and stiffness. Both the primary and key secondary outcomes were assessed using these valid and reliable measures [6,7].

Core physical performance measures

Physical performance measures were assessed via a 30-second chair stand test, 40 m (10 m x 4) fast-paced walk test, and stair climb test at baseline, week 12 and 24 weeks. Measures were performed as recommended by the Osteoarthritis Research Society International (OARSI) guidelines for clinical trials using the same equipment and same location [8]. The total number of chair stands within a 30-second (minimal clinically important difference, 2 stands) [9], the time to complete a 40 m fast-paced walking speed (m/s) with three turns (minimal clinically important difference, 0.3m/s) [9], and the time taken to ascend and descend a nine-step stair (with a step height of 20 cm and handrail support) were recorded.

Leg muscle strength

Participants stood on the back of a dynamometer platform with their backs against a wall and their knees flexed to 115 degrees and were asked to pull the handle as hard as possible. The muscles measured in this technique were the quadriceps and hip flexors.

Patient Health Questionnaire

Depression was assessed using the Patient Health Questionnaire (PHQ-9) at baseline, 12, and 24. The PHQ-9 is a validated tool for screening, diagnosing, monitoring, and measuring the severity of depression. It consists of nine questions based on the Diagnostic and Statistical Manual of mental disorders (DSM-IV) criteria for the diagnosis of major depressive disorders in patients with medical illnesses [10].

Patient global assessment

Patient global assessment was assessed over 12 and over 24 weeks using standard question, "Considering all the ways in which illness and health conditions may affect you at this time, please indicate on the line below how you are doing?", along with a 0–100mm VAS, where 0 is very well, and 100 is very poor [11].

PainDETECT questionnaire

Neuropathic pain was assessed using painDETECT questionnaire which uses a combination of VAS, Likert-type questions, and body diagram to ask about everyday frequency of symptoms such as "electric shocks" or "painful light touch" [12].

OARSI-OMERACT responder

The OARSI-OMERACT responder criteria were used to assess treatment response. Categorical variable representing responders and non-responders were generated. The responder status was determined based on the observed improvements in WOMAC pain, WOMAC function, and the patient's global assessment. The criteria for categorizing treatment responder included two possible scenarios: 1) an improvement of at least 50% in either pain or function, coupled with an absolute change of a minimum of 20 points on a scale of 0 to 100 in the respective WOMAC pain or function sub-scores or 2) fulfillment of at least two of the following conditions: a minimum of 20% improvement and an absolute change exceeding 10 points on a scale of 0 to 100 in the WOMAC pain score, a minimum of 20% improvement and an absolute change surpassing 10 points on a scale of 0 to 100 in the WOMAC function score, or a minimum of 20% improvement in the Patient Global Assessment score, coupled with an absolute change greater than 10 points on a scale of 0 to 100 [11].

Pain medication use

All participants were permitted to continue their ongoing medication regimens established during their screening visit for the duration of trial. Participants were instructed to maintain their medication as stable as possible throughout the study. However, if a participant necessitated an escalation in analgesic dosage, such adjustments were allowed. Any medication change was documented with reason, drug name, and dose. These changes were categorized into commencement or increased, discontinuation or decreased, or stable use or non-use, with the change in total number of pain medications. Rescue medication, paracetamol was made available if requested. Medications use was documented at baseline and during each follow-up periods.

Details of statistical analysis

Statistical methods

Analyses were performed using Stata version 18 (Stata Corporation, Inc., College Station, TX, USA) and a two-sided P value of 0.05 was deemed statistically significant.

Normality was assessed by examining the distribution of residuals, and homogeneity of variance was evaluated through visual inspection of plots of residuals versus fitted values.

1. Linear Mixed Effects Model (Repeated-measures mixed-effects model)

© 2025 Abafita BJ et al. *JAMA Network Open*.

We chose mixed-effects models for the primary analyses because they are well-suited to handle longitudinal data with repeated measures over time. These models account for the correlation within subjects by including random effects, which allows each participant to serve as their own control and reduces bias arising from individual differences. Moreover, mixed-effects models are flexible in accommodating missing data under the assumption that data are missing at random. Importantly, no imputation procedures for missing data were performed, as these were not pre-specified in the original study protocol.

STATA functions used: -mixed function in STATA (version 18) was used for the modelling of linear mixed effects model. The -margins command was used to calculate the estimated value of the outcome measure at each time point. The -lincom function was used to calculate the within- and between-group differences over time.

The outcomes for which we used linear mixed effects models were visual analog scale (VAS) knee pain, Western Ontario and McMaster University Index (WOMAC) subscales (pain, function, and stiffness), patient global assessment, Depression (as assessed by patient health questionaries), quality of life derived utility values, core physical performance measures (as assessed by 30 s chair stand test, 40 m fast walk test, and stair climb test), neuropathic pain (as assessed by painDETECT questionnaire) and leg muscle strength. First, the main effects are treatment group, time, and interaction of treatment group with time. The presence of time interaction allows us to estimate the treatment effect at different time points (each follow-up). Second, the model was also adjusted for baseline values for each continuous outcome, age, sex, and BMI in our analyses because these baseline variables are known to influence both the severity of knee OA and the response to interventions. Specifically, age and BMI are established risk factors for OA progression and pain perception, while sex differences can affect pain tolerance and functional outcomes. Additionally, we believe that the effects of these baseline variables on the change in outcome measures are not static but vary over time, a phenomenon often referred to as "time-varying effects." By accounting for these time-varying effects, we aim to more accurately model the relationship between the interventions and the outcomes, thereby reducing potential confounding and improving the precision of our estimates. Furthermore, the interaction terms for each confounder with time were added. Third, participant ID was included as a random effect allowing random intercepts. This addresses the correlation within repeated measures. Fourth, the mixed-effects model was fitted using default setting of -mixed function (independent covariance and the restricted maximum likelihood method).

2. Binomial regression

For the binomial regression, -glm function was used to estimate the risk ratio or risk difference by specifying the options family () and link (). Treatment group was included as the univariate predictor of OARSI-OMERACT responders.

- risk ratio by -glm with family (binomial) and link (log),
- risk difference by -glm with family (binomial) and link (identity).

3. Priori subgroup analysis

A pre-specified subgroup analysis based on the absence or presence of neuropathic-like pain was performed to investigate which group better responded to the intervention for primary and key secondary outcomes over 12 and 24 weeks. These sub-group analyses were performed by adding an extra layer of interaction to allow the main effects and interactions to vary based on the presence or absence of neuropathic-like pain. This involved using a three-way interaction between treatment arm, week, and neuropathic pain in the linear mixed-effects model.

Results

A priori subgroup analyses

The results of priori subgroup analyses (eTable 3 & 4) indicate that the effects of yoga and strengthening exercises on the primary outcome of change in mean VAS knee pain were not significantly different in participants with possible neuropathic-like pain versus those without. There were no statistically significant interactions between the intervention and possible neuropathic-like pain for both the VAS knee pain and the WOMAC subscales (pain, function, and stiffness). Over 24 weeks, yoga showed a significant improvement in WOMAC pain, function and stiffness compared to strengthening exercises among participants with unlikely neuropathic pain. Additionally, over 24-weeks, the yoga group showed a significant improvement in WOMAC function among participants with possible neuropathic-like pain, when compared with the strengthening exercises group.

© 2025 Abafita BJ et al. *JAMA Network Open*.

Supplementary Tables

eTable 1. Sensitivity analyses based on the per-protocol population on change in study endpoints between the yoga and strengthening groups over 12- and 24-weeks[a]

**Outcome**	**Mean (95% CI)**					
	**Yoga**		**Strengthening**		Absolute between group difference	P value
	At baseline	Within group change	At baseline	Within group change		
VAS knee pain score (0-100), mm						
Week 12	51.5 (46.9 to 56.2) [n=45]	-18.0 (-22.9 to -13.1)	54.7 (49.6 to 59.9) [n=44]	-16.4 (-21.4 to -11.5)	-1.5 (-8.6 to 5.5)	0.67
Week 24	51.2 (46.3 to 56.2) [n=41]	-24.6 (-29.6 to -19.6)	55.2 (49.5 to 60.8) [n=38]	-19.3 (-24.6 to -14.1)	-5.3 (-12.7 to 2.1)	0.16
WOMAC pain score (0-500), mm						
Week 12	199.6 (173.4 to 225.8) [n=45]	-59.4 (-77.7 to -41.1)	189.2 (162.7 to 215.6) [n=43]	-48.9 (-67.6 to -30.2)	-10.5 (-36.8 to 15.9)	0.44
Week 24	195.9 (168.5 to 223.3) [n=41]	-90.5 (-109.2 to -71.8)	195.6 (166.7 to 224.4) [n=38]	-50.7 (-66.3 to -27.6)	**-39.8 (-67.0 to -12.7)**	**0.004**
WOMAC function (0-1700), mm						
Week 12	627.6 (531.9 to 723.4) [n=45]	-177.9 (-239.6 to -116.3)	613.9 (518.8 to 708.9) [n=42]	-119.2 (-183.0 to -55.3)	-58.8 (-148.1 to 30.6)	0.20
Week 24	609.9 (510.6 to 709.2) [n=41]	-255.8 (-318.8 to -192.9)	645.6 (544.7 to 746.4) [n=38]	-139.8 (-205.4 to -74.1)	**-116.0 (-207.9 to -24.2)**	**0.01**
WOMAC stiffness (0-200), mm						
Week 12	83.8 (71.9 to 95.7) [n=45]	-26.4 (-35.6 to -17.3)	95.1 (84.3 to 105.9) [n=41]	-20.2 (-29.8 to -10.5)	-6.3 (-19.8 to 7.2)	0.36
Week 24	82.1 (69.8 to 94.5) [n=41]	-39.2 (-48.7 to -29.7)	95.1 (83.4 to 106.9) [n=38]	-24.2 (-34.1 to -14.2)	**-15.0 (-30.0 to -1.0)**	**0.04**
Patient global assessment (0-100), mm						
Week 12	33.4 (26.8 to 40.1) [n=45]	-5.9 (-11.1 to -0.6)	29.1 (23.0 to 35.2) [n=41]	-2.4 (-7.9 to 3.1)	-3.5 (-11.2 to 4.2)	0.37

© 2025 Abafita BJ et al. JAMA Network Open.

Week 24	32.1 (25.1 to 39.2) [n=41]	-11.3 (-16.7 to -5.9)	29.4 (22.7 to 36.1) [n=38]	-3.8 (-9.4 to 1.9)	-7.5 (-15.4 to 0.4)	0.06
PainDETECT questionnaire (0-38)						
Week 12	7.2 (5.8 to 8.5) [n=44]	-1.2 (-2.3 to -0.03)	7.5 (6.0 to 9.1) [n=39]	-0.4 (-1.6 to 0.9)	-0.8 (-2.5 to 0.9)	0.34
Week 24	7.2 (5.6 to 8.8) [n=35]	-2.1 (-3.3 to -0.9)	7.3 (5.8 to 8.8) [n=34]	-1.6 (-2.8 to -0.4)	-0.6 (-2.3 to 1.2)	0.53
PHQ-9 score (0-27)						
Week 12	3.7 (2.7 to 4.7) [n=45]	-1.0 (-1.6 to -0.3)	3.4 (2.4 to 4.4) [n=41]	0.1 (-0.6 to 0.8)	**-1.1 (-2.1 to -0.1)**	**0.03**
Week 24	3.7 (2.6 to 4.9) [n=39]	-0.6 (-1.3 to 0.2)	3.4 (2.3 to 4.5) [n=37]	0.02 (-0.7 to 0.8)	-0.6 (-1.6 to 0.5)	0.29
AQol-8D utility score						
Week 12	0.71 (0.67 to 0.76) [n=44]	0.05 (0.03 to 0.07)	0.76 (0.71 to 0.80) [n=40]	0.03 (0.01 to 0.05)	0.02 (-0.01 to 0.05)	0.24
Week 24	0.72 (0.66 to 0.77) [n=36]	0.06 (0.04 to 0.09)	0.76 (0.71 to 0.80) [n=36]	0.03 (0.00 to 0.05)	**0.04 (0.00 to 0.07)**	**0.05**
EQ-5D-5L index score						
Week 12	0.83 (0.80 to 0.86) [n=45]	0.03 (0.01 to 0.05)	0.84 (0.80 to 0.88) [n=40]	0.00 (-0.02 to 0.03)	0.03 (-0.01 to 0.06)	0.13
Week 24	0.83 (0.79 to 0.86) [n=37]	0.05 (0.02 to 0.07)	0.85 (0.81 to 0.89) [n=36]	0.02 (-0.01 to 0.04)	0.03 (-0.01 to 0.07)	0.10
Leg muscle strength (kg)						
Week 12	61.8 (56.1 to 67.6) [n=41]	18.3 (13.3 to 23.2)	59.1 (54.5 to 63.8) [n=45]	21.8 (17.1 to 26.5)	-3.5 (-10.5 to 3.4)	0.32
Week 24	66.7 (54.3 to 79.2) [n=40]	21.9 (16.9 to 26.9)	58.2 (49.7 to 66.7) [n=43]	23.8 (19.0 to 28.6)	-1.8 (-8.9 to 5.2)	0.61
30-s chair stand test						
Week 12	10.7 (9.9 to 11.6) [n=42]	2 (1.2 to 2.7)	11.0 (10.3 to 11.6) [n=45]	1.8 (1.1 to 2.4)	0.2 (-0.8 to 1.2)	0.70
Week 24	10.8 (9.9 to 11.6) [n=43]	3.5 (2.8 to 4.2)	11.0 (10.3 to 11.7) [n=43]	3.2 (2.5 to 3.9)	0.3 (-0.7 to 1.3)	0.56
40-m fast-paced walk test(m/s)						
Week 12	1.82 (1.73 to 1.91) [n=44]	0.07 (0.03 to 0.11)	1.80 (1.73 to 1.86) [n=44]	0.05 (0.01 to 0.09)	0.02 (-0.04 to 0.08)	0.48

© 2025 Abafita BJ et al. *JAMA Network Open*.

Week 24	1.82 (1.74 to 1.91) [n=43]	0.13 (0.09 to 0.17)	1.80 (1.74 to 1.87) [n=42]	0.07 (0.03 to 0.11)	**0.06 (0.00 to 0.12)**	**0.05**
Stair climbs test(s)						
Week 12	10.9 (9.1 to 12.6) [n=42]	-1.6 (-2.1 to -1.1)	10.7 (9.8 to 11.6) [n=45]	-1.3 (-1.7 to -0.8)	-0.3 (-1.0 to 0.4)	0.41
Week 24	11.2 (9.3 to 13.0) [n=43]	-2.3 (-2.8 to -1.8)	10.8 (9.9 to 11.7) [n=43]	-1.8 (-2.3 to -1.4)	-0.4 (-1.1 to 0.3)	0.24
OMERACT-OARSI responders, no. %)						
Week 12	44.4 (38.9 to 50.1)		32.6 (27.3 to 38.2)		11.9 (-8.3 to 32.1)	0.25
Week 24	58.5 (42.1 to 73.7)		50 (33.4 to 66.6)		8.5 (-13.4 to 30.4)	0.45

[a] Sensitivity analyses were performed on complete-case participants with baseline data who completed both the week-12 and week-24 assessments. All values are means, with the 95% confidence intervals except Osteoarthritis Research Society International–Outcome Measure in 4 Rheumatology Clinical Trial (OARSI-OMERACT) responders. For continuous outcome variables, results are analysed by linear mixed-effect model and models were adjusted for the baseline value of the corresponding outcome, age, sex**,** and BMI. Missing data at follow-up were addressed using linear mix-effects modelling (no imputation).

© 2025 Abafita BJ et al. *JAMA Network Open*.

eTable 2. Change in knee symptoms (VAS & WOMAC) from baseline to all timepoints

	Mean (95% CI)					
	**Yoga (n=58)**		**Strengthening (n=59)**		Absolute between group difference [a]	
	At baseline	Within group change [a]	At baseline	Within group change		P value
VAS knee pain score (0-100), mm [b]	[n=57]		[n=58]			
Baseline to 4 weeks	54.2 (52.7 to 55.7)	-10.6 (-15.2 to -5.9)	53.4 (51.7 to 55.0)	-7.3 (-12.0 to -2.6)	-3.3 (-10.0 to 3.4)	0.33
Baseline to 8 weeks	54.2 (52.7 to 55.7)	-14.9 (-19.8 to -10.0)	53.4 (51.7 to 55.0)	-10.5 (-15.3 to -5.7)	-4.4 (-11.4 to 2.5)	0.21
Baseline to 12 weeks	54.2 (52.7 to 55.7)	-17.7 (-22.4 to -13.0)	53.4 (51.7 to 55.0)	-16.7 (-21.4 to -11.9)	-1.1 (-7.8 to 5.7)	0.76
Baseline to 16 weeks	54.2 (52.7 to 55.7)	-23.4 (-28.2 to -18.5)	53.4 (51.7 to 55.0)	-13.2 (-18.3 to -8.0)	**-10.2 (-17.4 to -3.0)**	**0.005**
Baseline to 20 weeks	54.2 (52.7 to 55.7)	-25.2 (-30.3 to -20.2)	53.4 (51.7 to 55.0)	-15.7 (-20.8 to -10.5)	**-9.6 (-16.9 to -2.2)**	**0.01**
Baseline to 24 weeks	54.2 (52.7 to 55.7)	-24.4 (-29.3 to -19.6)	53.4 (51.7 to 55.0)	-18.6 (-23.6 to -13.6)	-5.8 (-12.9 to 1.2)	0.11
WOMAC pain score (0-500), mm [c]	[n=57]		[n=58]			
Baseline to 4 weeks	217.7 (209.1 to 226.3)	-36.3 (-53.7 to -18.9)	197.3 (189.4 to 205.1)	-21.5 (-39.0 to -4.0)	-14.8 (-39.8 to 10.1)	0.25
Baseline to 8 weeks	217.7 (209.1 to 226.3)	-50.7 (-68.9 to -32.4)	197.3 (189.4 to 205.1)	-25.6 (-43.7 to -7.6)	-25.0 (-51.0 to 0.9)	0.06
Baseline to 12 weeks	217.7 (209.1 to 226.3)	-59.8 (-77.4 to -44.2)	197.3 (189.4 to 205.1)	-49.2 (-67.0 to -31.3)	-10.6 (-35.9 to 14.6)	0.41
Baseline to 16 weeks	217.7 (209.1 to 226.3)	-73.9 (-92.0 to -55.7)	197.3 (189.4 to 205.1)	-40.7 (-59.8 to -21.6)	**-33.1 (-59.7 to -6.6)**	**0.02**
Baseline to 20 weeks	217.7 (209.1 to 226.3)	-83.5 (-102.3 to -64.6)	197.3 (189.4 to 205.1)	-40.9 (-60.2 to -21.7)	**-42.5 (-69.7 to -15.3)**	**0.002**
Baseline to 24 weeks	217.7 (209.1 to 226.3)	-92.4 (-110.5 to -74.3)	197.3 (189.4 to 205.1)	-47.9 (-66.6 to -29.2)	**-44.5 (-70.7 to -18.3)**	**0.001**
WOMAC function (0-1700), mm [d]	[n=57]		[n=58]			
Baseline to 4 weeks	702.5 (670.5 to 734.5)	-100.9 (-160.1 to -41.8)	670.1 (641.3 to 698.8)	-57.9 (-117.7 to 1.8)	-43.0 (-127.8 to 41.9)	0.32

© 2025 Abafita BJ et al. JAMA Network Open.

Baseline to 8 weeks	702.5 (670.5 to 734.5)	-111.8 (-173.7 to -49.8)	670.1 (641.3 to 698.8)	-84.2 (-145.6 to -22.7)	-27.6 (-115.8 to 60.5)	0.54
Baseline to 12 weeks	702.5 (670.5 to 734.5)	-183.2 (-243.1 to -123.4)	670.1 (641.3 to 698.8)	-120.1 (-181.8 to -58.9)	-63.1 (-149.3 to 23.0)	0.15
Baseline to 16 weeks	702.5 (670.5 to 734.5)	-225.6 (-287.4 to -163.7)	670.1 (641.3 to 698.8)	-119.4 (-184.6 to -54.3)	**-106.1 (-196.7 to -15.6)**	**0.02**
Baseline to 20 weeks	702.5 (670.5 to 734.5)	-264.9 (-329.1 to -200.6)	670.1 (641.3 to 698.8)	-127.6 (-193.3 to -61.9)	**-137.2 (-229.9 to -44.6)**	**0.01**
Baseline to 24 weeks	702.5 (670.5 to 734.5)	-268.5 (-330.2 to -206.9)	670.1 (641.3 to 698.8)	-129.6 (-193.3 to -65.9)	**-139.0 (-228.3 to -49.7)**	**0.002**
WOMAC stiffness score (0-200), mm [e]	[n=57]		[n=57]			
Baseline to 4 weeks	91.0 (87.1 to 95.0)	-16.8 (-25.5 to -8.2)	94.2 (90.8 to 97.6)	-7.2 (-16.1 to 1.7)	-9.7 (-22.3 to 3.0)	0.14
Baseline to 8 weeks	91.0 (87.1 to 95.0)	-13.8 (-22.9 to -4.6)	94.2 (90.8 to 97.6)	-19.8 (-29.0 to -10.6)	6.1 (-7.2 to 19.3)	0.37
Baseline to 12 weeks	91.0 (87.1 to 95.0)	-26.6 (-35.4 to -17.8)	94.2 (90.8 to 97.6)	-18.4 (-27.6 to -9.3)	-8.2 (-21.1 to 4.7)	0.22
Baseline to 16 weeks	91.0 (87.1 to 95.0)	-31.2 (-40.4 to -22.1)	94.2 (90.8 to 97.6)	-18.3 (-28.0 to -8.6)	-12.9 (-26.5 to 0.7)	0.06
Baseline to 20 weeks	91.0 (87.1 to 95.0)	-35.3 (-44.9 to -25.8)	94.2 (90.8 to 97.6)	-23.9 (-33.7 to -14.2)	-11.4 (-25.3 to 2.5)	0.11
Baseline to 24 weeks	91.0 (87.1 to 95.0)	-40.3 (-49.4 to -31.2)	94.2 (90.8 to 97.6)	-22.6 (-32.1 to -13.2)	**-17.6 (-30.9 to -4.3)**	**0.009**

[a] The within-group change and between-group difference were calculated in participants with baseline data of the outcome. Missing data at follow-up were addressed using linear mixed-effect model (no imputation). Models were adjusted for the baseline value of the corresponding outcome, age, sex and BMI.
[b] Visual analog scale (VAS). Range from 0 to 100; higher scores indicate more severe symptoms. Analysed in participants with data on pain score at baseline (n=117).
[c] Western Ontario and McMaster University Osteoarthritis (WOMAC) pain. Range from 0 to 500; higher scores indicate more severe symptoms. The index relies on a self-administered questionnaire reflecting pain. The pain subscale measures 5 dimensions: walking on a flat surface, going up and down stairs, at night while in bed, sitting or lying, and standing upright. Analysed in participants with data on pain score at baseline (n=117).
[d] Western Ontario and McMaster University Osteoarthritis (WOMAC) function. Range is 0 to 1700; higher scores indicate more severe symptoms. The index relies on a self-administered questionnaire reflecting limitations to physical function. The function subscale measures 17 dimensions. Analysed in participants with data on function at baseline (n=117).
[e] Western Ontario and McMaster University Osteoarthritis (WOMAC) stiffness. Range is 0 to 200; higher scores indicate more severe symptoms. The index relies on a self-administered questionnaire reflecting stiffness. The stiffness subscale measures 2 dimensions: After walking and later in the day. Analysed in participants with data on stiffness at baseline (n=117).

© 2025 Abafita BJ et al. *JAMA Network Open*.

eTable 3. Change in knee symptoms (VAS & WOMAC) from baseline to all timepoints in participants with possible neuropathic pain and without neuropathic pain at baseline

	Mean (95% CI)					
	**Yoga**		**Strengthening**		Absolute between group difference [a]	
	At baseline	Within group change [a]	At baseline	Within group change [a]		P value
Possible Neuropathic pain						
VAS knee pain score (0-100), mm [b]	[n=8]		[n=12]			
Baseline to 4 weeks	65.4 (58.2 to 72.6)	-12.3 (-23.0 to -1.6)	58.3 (47.8 to 68.7)	-18.6 (-27.6 to -9.6)	6.3 (-8.0 to 20.6)	0.39
Baseline to 8 weeks	65.4 (58.2 to 72.6)	-22.5 (-33.8 to -11.1)	58.3 (47.8 to 68.7)	-10.6 (-22.3 to 1.1)	-11.9 (-29.3 to 5.4)	0.18
Baseline to 12 weeks	65.4 (58.2 to 72.6)	-20.9 (-32.2 to -9.6)	58.3 (47.8 to 68.7)	-14.3 (-25.4 to -3.1)	-6.6 (-23.8 to 10.6)	0.45
Baseline to 16 weeks	65.4 (58.2 to 72.6)	-35.1 (-47.3 to -22.9)	58.3 (47.8 to 68.7)	-10.2 (-22.0 to 1.6)	**-24.9 (-43.9 to -5.9)**	**0.01**
Baseline to 20 weeks	65.4 (58.2 to 72.6)	-26.8 (-39.0 to -14.6)	58.3 (47.8 to 68.7)	-16.4 (-29.0 to -3.8)	-10.4 (-30.0 to 9.2)	0.30
Baseline to 24 weeks	65.4 (58.2 to 72.6)	-30.1 (-42.4 to -17.9)	58.3 (47.8 to 68.7)	-21.8 (-35.7 to -8.0)	-8.3 (-29.1 to 12.5)	0.43
WOMAC pain score (0-500), mm [c]	[n=8]		[n=12]			
Baseline to 4 weeks	297.8 (237.5 to 358.0)	-72.1 (-112.4 to -31.8)	234.3 (194.6 to 274.1)	-41.4 (-74.9 to -7.9)	-30.7 (-85.1 to 23.7)	0.27
Baseline to 8 weeks	297.8 (237.5 to 358.0)	-91.3 (-133.3 to -49.2)	234.3 (194.6 to 274.1)	-41.3 (-86.4 to 3.8)	-49.9 (-116.4 to 16.5)	0.14
Baseline to 12 weeks	297.8 (237.5 to 358.0)	-63.9 (-106.0 to -21.7)	234.3 (194.6 to 274.1)	-84.0 (-126.0 to -41.9)	20.1 (-44.7 to 84.8)	0.54
Baseline to 16 weeks	297.8 (237.5 to 358.0)	-128.2 (-173.1 to -83.4)	234.3 (194.6 to 274.1)	-56.3 (-100.3 to -12.3)	**-72.0 (-142.2 to -1.7)**	**0.05**
Baseline to 20 weeks	297.8 (237.5 to 358.0)	-92.8 (-137.7 to -47.8)	234.3 (194.6 to 274.1)	-59.0 (-105.6 to -12.4)	-33.8 (-106.2 to 38.6)	0.36
Baseline to 24 weeks	297.8 (237.5 to 358.0)	-113.2 (-158.4 to -68.0)	234.3 (194.6 to 274.1)	-76.5 (-127.8 to -25.3)	-36.6 (-113.7 to -40.4)	0.35
Negative Neuropathic pain						

© 2025 Abafita BJ et al. JAMA Network Open.

VAS knee pain score (0-100), mm [b]	[n=46]		[n=43]			
Baseline to 4 weeks	51.3 (49.7 to 52.9)	-10.8 (-15.9 to -5.6)	53.0 (51.1 to 54.9)	-4.4 (-9.9 to 1.1)	-6.3 (-14.0 to 1.3)	0.11
Baseline to 8 weeks	51.3 (49.7 to 52.9)	-13.2 (-18.6 to -7.8)	53.0 (51.1 to 54.9)	-10.6(-16.3 to -5.0)	-2.6(-10.6 to 5.4)	0.53
Baseline to 12 weeks	51.3 (49.7 to 52.9)	-19.0 (-24.2 to -13.7)	53.0 (51.1 to 54.9)	-15.3 (-20.7 to -9.8)	-3.7 (-11.4 to 4.0)	0.35
Baseline to 16 weeks	51.3 (49.7 to 52.9)	-23.0 (-28.4 to -17.6)	53.0 (51.1 to 54.9)	-11.9 (-17.7 to -6.1)	**-11.1 (-19.1 to -3.0)**	**0.007**
Baseline to 20 weeks	51.3 (49.7 to 52.9)	-25.4 (-31.0 to -19.7)	53.0 (51.1 to 54.9)	-14.7 (-20.5 to -8.9)	**-10.6 (-18.9 to -2.4)**	**0.01**
Baseline to 24 weeks	51.3 (49.7 to 52.9)	-24.3 (-29.6 to -18.9)	53.0 (51.1 to 54.9)	-16.9 (-22.6 to -11.3)	-7.3 (-15.2 to 0.6)	0.07
WOMAC pain score (0-500), mm [c]	[n=46]		[n=43]			
Baseline to 4 weeks	197.2 (188.4 to 206.1)	-28.1 (-47.2 to -8.9)	188.9 (179.3 to 198.4)	-16.1 (-36.4 to 4.2)	-12.0 (-40.3 to 16.3)	0.41
Baseline to 8 weeks	197.2 (188.4 to 206.1)	-43.5 (-63.4 to -23.5)	188.9 (179.3 to 198.4)	-21.2 (-41.9 to -0.6)	-22.2 (-51.5 to 7.0)	0.14
Baseline to 12 weeks	197.2 (188.4 to 206.1)	-61.4 (-80.7 to -42.1)	188.9 (179.3 to 198.4)	-41.5 (-61.5 to -21.6)	-19.9 (-48.0 to 8.2)	0.17
Baseline to 16 weeks	197.2 (188.4 to 206.1)	-66.6 (-86.4 to -46.7)	188.9 (179.3 to 198.4)	-33.3 (-54.6 to -11.9)	**-33.3 (-62.8 to -3.9)**	**0.03**
Baseline to 20 weeks	197.2 (188.4 to 206.1)	-80.7 (-101.5 to -59.9)	188.9 (179.3 to 198.4)	-34.3 (-55.7 to -12.8)	**-46.5 (-76.7 to -16.2)**	**0.003**
Baseline to 24 weeks	197.2 (188.4 to 206.1)	-88.5 (-108.3 to -68.7)	188.9 (179.3 to 198.4)	-39.2 (-59.9 to -18.6)	**-49.2 (-78.2 to -20.3)**	**0.001**

[a] The within-group change and between-group difference were calculated in participants with baseline data of the outcome. Missing data at follow-up were addressed using linear mixed-effect model (no imputation). Models were adjusted for the baseline value of the corresponding outcome, age, sex and BMI.

[b] Visual analog scale (VAS). Range from 0 to 100; higher scores indicate more severe symptoms. Analysed in participants with data on pain score at baseline (n=117).

[c] Western Ontario and McMaster University Osteoarthritis Index (WOMAC) pain. Range from 0 to 500; higher scores indicate more severe symptoms. The index relies on a self-administered questionnaire reflecting pain. The pain subscale measures 5 dimensions: walking on a flat surface, going up and down stairs, at night while in bed, sitting or lying, and standing upright. Analysed in participants with data on pain score at baseline (n=117).

© 2025 Abafita BJ et al. *JAMA Network Open*.

eTable 4. Change in pain medication and supplements over 12- and 24-weeks follow-up by treatment groups

Medication	Yoga, no./total no. (%)			Strengthening, no./total no. (%)		
	Stable	Commenced or Increased	Discontinued or Decreased	Stable	Commenced or Increased	Discontinued or Decreased
Paracetamol						
Week 12	42/45 (95.6)	2/45 (4.4)	1/45 (2.2)	39/46 (84.8)	2/46 (4.3)	5/46 (10.9)
Week 24	39/43 (90.7)	3/43 (7)	1/43 (2.3)	39/44 (88.6)	1/44 (2.3)	4/44 (9.1)
NSAIDs [a]						
Week 12	39/45 (86.7)	3/45 (6.7)	3/45 (6.7)	41/46 (89.1)	2/46 (4.3)	3/46 (6.5)
Week 24	40/43 (93)	1/43 (2.3)	2/43 (4.7)	40/44 (91)	3/44 (6.8)	1/44 (2.3)
Other agents [b]						
Week 12	43/45 (95.6)	2/45 (4.4)	0/45 (0.0)	46/46 (100)	0/46 (0.0)	0/46 (0.0)
Week 24	40/43 (93)	1/43 (2.3)	2/43 (4.7)	42/44 (95.5)	1/44 (2.3)	1/44 (2.3)
Topical agent						
Week 12	45/45 (100)	0/45 (0.0)	0/45 (0.0)	46/46 (100)	0/46 (0.0)	0/46 (0.0)
Week 24	42/43 (97.7)	0/43 (0.0)	1/43 (2.3)	44/44 (100)	0/44 (0.0)	0/44 (0.0)
Dietary Supplements [c]						
Week 12	43/45 (95.6)	0/45 (0.0)	2/45 (4.4)	43/46 (93.5)	2/46 (4.3)	1/46 (2.2)
Week 24	43/43 (100)	0/43 (0.0)	0/43 (0.0)	42/44 (95.5)	1/44 (2.3)	1/44 (2.3)

[a] NSAIDs, nonsteroidal anti-inflammatory drugs.
[b] Included: opioids, gabapentinoids and steroids
[c] Included: turmeric, fish oil, glucosamines, zingiber officinale, piper nigrum, algae extract, collagen peptides, ashwagandha, garlic and horsedish, ginko biloba and co-enzymes.

© 2025 Abafita BJ et al. JAMA Network Open.

eTable 5. Adherence measures over the first 12 and 24 weeks for participants in yoga and strengthening groups

	Week 1-12 [a]		Week 13-24 [a]		Week 1-24 [b]	
**Adherence Measures**	**Yoga**	**Strengthening**	**Yoga**	**Strengthening**	**Yoga**	**Strengthening**
Total no. of sessions completed; Mean (SD)	25.1±9.8	26.0±8.6	25.3±10.3	21.6±10.6	45.4±22.4	43.4±20.0
Percentage of sessions completed; Mean (SD) %	69.8 (27.2)	71.9 (23.9)	70.2 (28.5)	60.0 (29.5)	63.1 (31.1)	60.3 (27.8)
Total no. of sessions per week; Mean (SD)	2.5±0.82	2.5±0.85	2.3±1.0	2.0±1.1	2.4±0.94	2.3±1.0
Percentage of sessions completed per week; Mean (SD) % [c]	84.1 (26.7)	82.0 (28.5)	76.7 (34.7)	66.7 (37.2)	80.7 (31.3)	75.0 (33.6)

[a] Total no. of exercise sessions completed from week 1-12 and week 13-24 as recorded by participants in a weekly survey (from a maximum of 36 and converted to a percentage).

[b] Total no. of exercise sessions completed from week 1-24 as recorded by participants in a weekly survey (from a maximum of 72 and converted to a percentage).

[c] Number of exercise sessions per week completed as recorded by participants in a weekly survey (from a maximum of 3 and converted to a percentage).

© 2025 Abafita BJ et al. *JAMA Network Open*.

eTable 6. Participants' fidelity to intervention protocol across groups, reported as n (%) unless otherwise indicated

Measure	Yoga	%	Strengthening exercises	%
All prescribed sessions completed from week 1–11(Group supervised sessions)	416/507	82.1	415/507	81.9
All prescribed sessions completed from week 13–23 (Home unsupervised sessions)	264/445	59.3	178/419	42.5
All prescribed sessions completed from week 1–24	680/957	71	593/926	64
Sessions where the RPE was above 5 from week 1–11 (Group supervised sessions)	380/493	77.1	399/488	81.8
Sessions where the RPE was above 5 from week 13–23 (Home unsupervised sessions)	343/434	79	320/392	81.6
Sessions where the RPE was above 5 from week 1–24	723/927	78	719/880	81.7

N.B: The above report is based on weekly surveys, where participants were asked to report:

If they completed all prescribed sessions in the previous week.

If they replaced any missed group sessions.

The data reflect participants' self-reported adherence and are not based on the actual number of sessions attended.

RPE: Rating of Perceived Exertion, recorded by participants for each prescribed exercise/yoga session they attended.

© 2025 Abafita BJ et al. JAMA Network Open.

Supplementary Figures

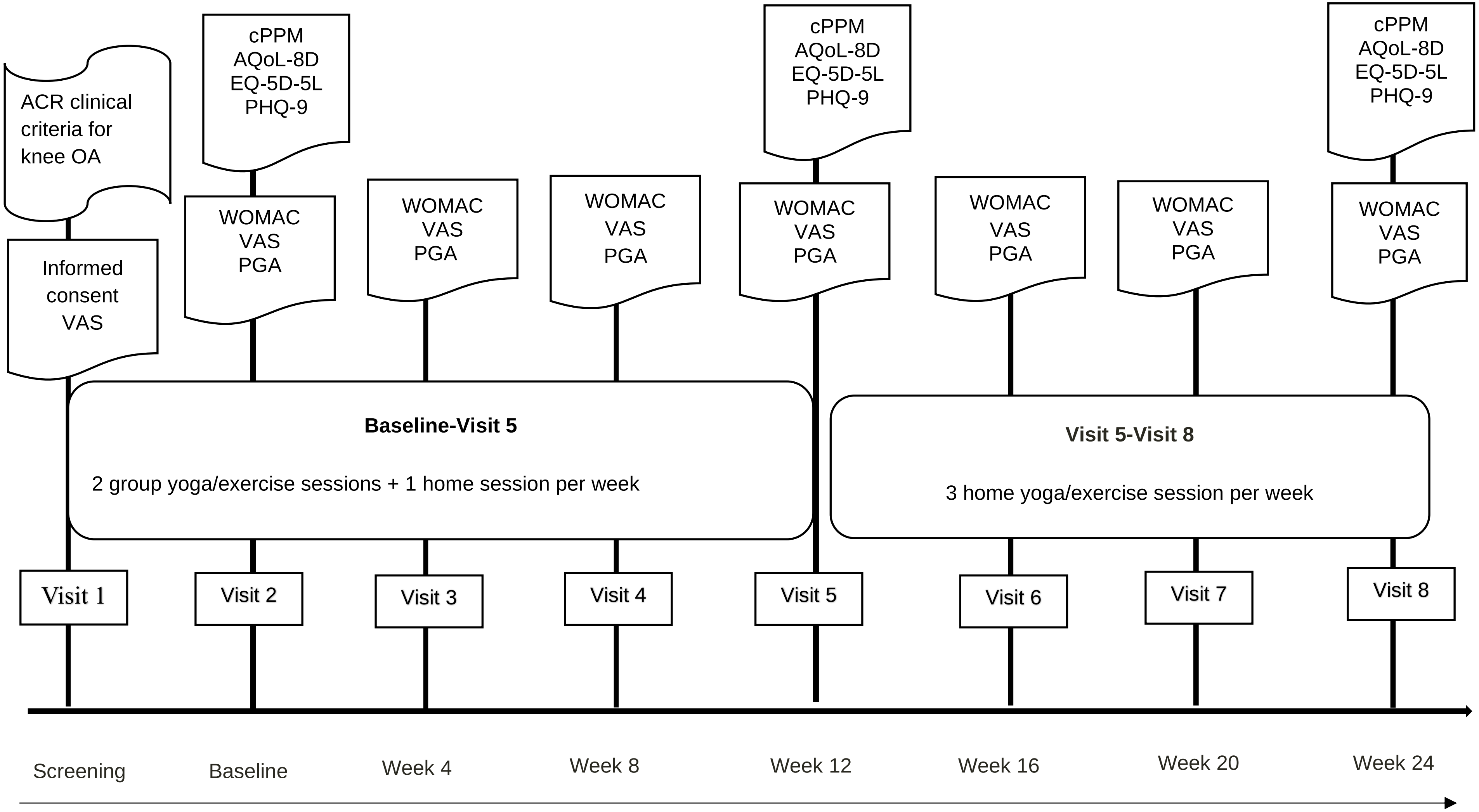

eFigure 1. Study schedule of the trial

Visual analog scale (VAS), Western Ontario and McMaster Universities Osteoarthritis Index (WOMAC), Patient global assessment (PGA), Core physical performance measures (cPPM), Assessment of quality of life (AQoL), EURoQol-5 dimensions-5 levels (EQ-5D-5L), and Patient health questionnaire (PHQ-9).

© 2025 Abafita BJ et al. JAMA Network Open.

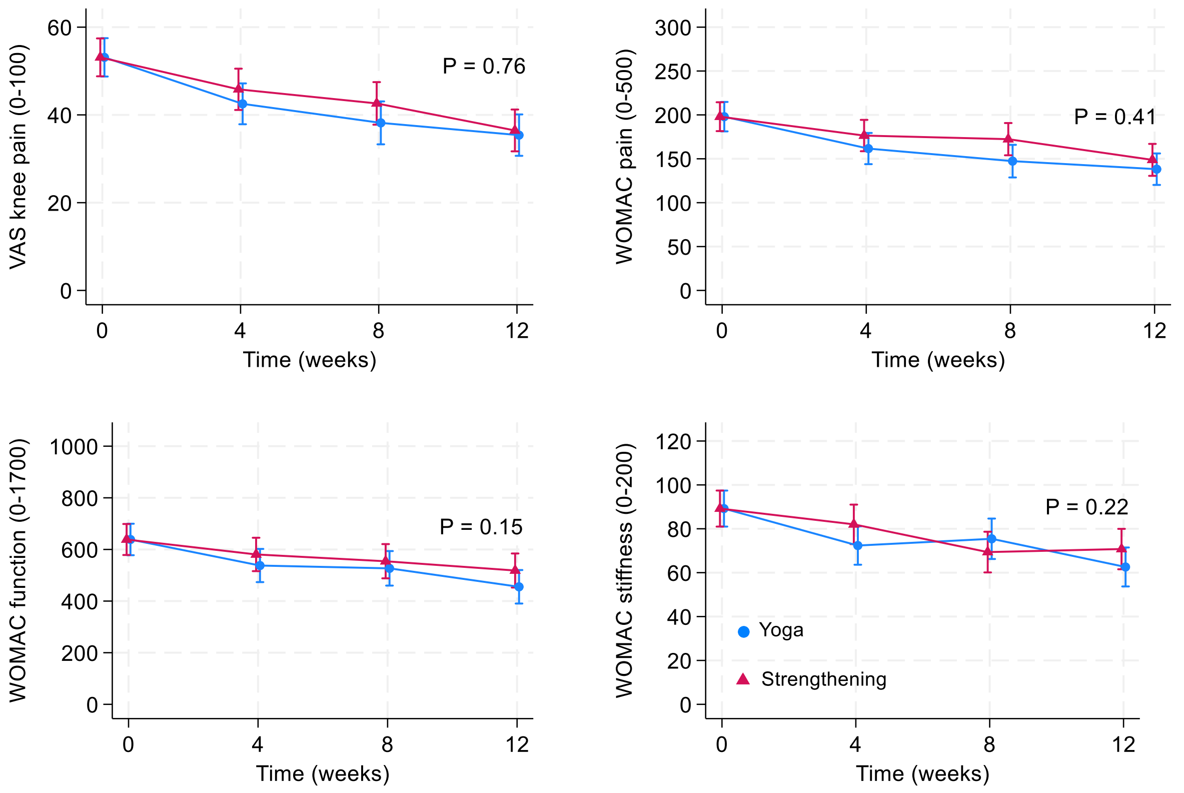

eFigure 2. Mean VAS knee pain and WOMAC subscale scores in the yoga and strengthening groups over 12 weeks

Outcome scores are shown for the yoga group (circle) and the strengthening group (triangles). Data are estimates from linear mixed effect models and all 117 participants were included in the analysis. Values are adjusted means, error bars indicate 95% CIs, and p-values correspond to between-group differences observed at 12 weeks. Measurements were obtained at baseline, 4 weeks, 8 weeks, and 12 weeks. Sores on VAS range from 0 to 100, with higher scores indicating more severe symptoms. Score on WOMAC pain ranged from 0 to 500; on WOMAC function, from 0 to 1700 and on WOMAC stiffness, from 0 to 200, all 3 with higher scores indicating more severe symptoms.

© 2025 Abafita BJ et al. JAMA Network Open.

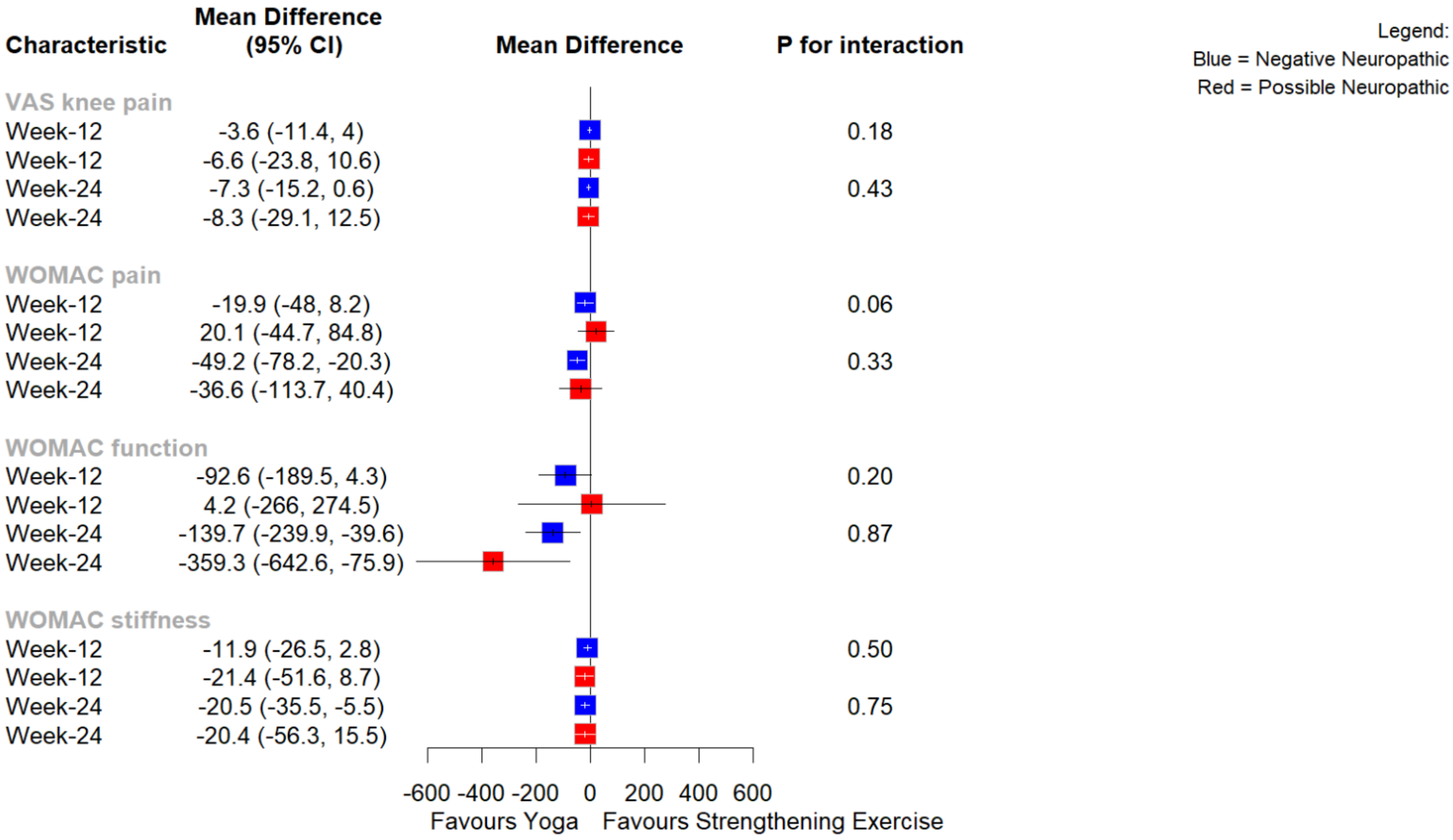

© 2025 Abafita BJ et al. *JAMA Network Open*.

eFigure 3. Change in VAS and WOMAC scores over 12 and 24 weeks, stratified by baseline PainDETECT score, for the yoga and strengthening groups

Continuous outcome variables are analysed using linear mixed-effect model adjusted for age, sex, BMI, and baseline value of the corresponding outcome. Missing data at follow-up were addressed using linear mixed-effect model (no imputation).
Visual analog score (VAS). Ranges from 0(no pain) to 100 (worst pain possible); higher scores indicate worse pain; MCID is 15.
Western Ontario and McMaster Universities Osteoarthritis Index (WOMAC) for pain (range 0-500), function (range 0-1700) and stiffness (range 0-200); higher scores indicate a more severe symptom.
A negative within group change indicates improvement. For between groups difference, a negative difference favors the yoga group.
P-value for interaction were calculated from linear mixed effect model adjusted for baseline value of corresponding outcome, sex, age, and BMI.
Subgroup was based on painDETECT a 13-item screening survey for neuropathic pain. Range from -1 to 38; category: <12 indicates negative neuropathic pain; ≥ 12 indicates possible neuropathic pain

© 2025 Abafita BJ et al. *JAMA Network Open*.

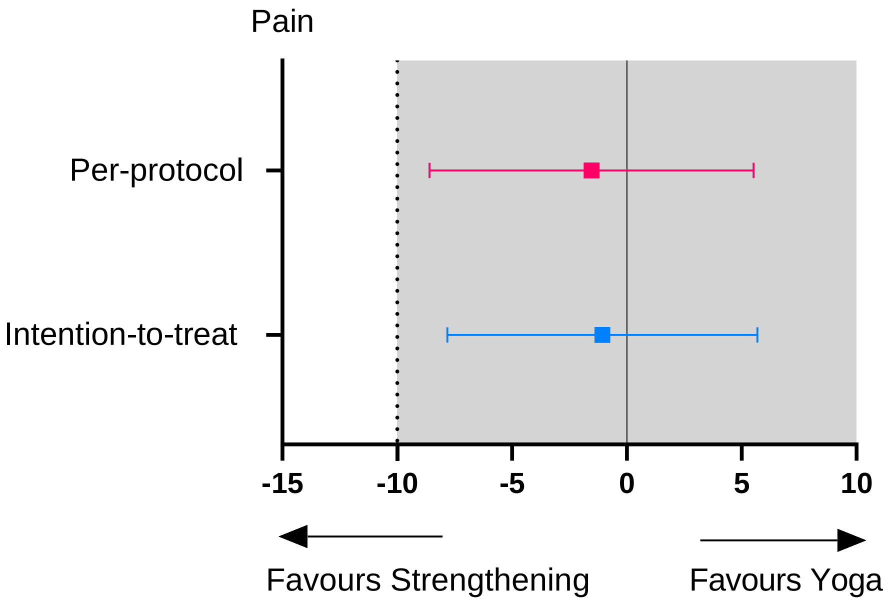

eFigure 4. Non-inferiority analysis of VAS knee pain score at 12 weeks

Data represent between group mean difference in change (95% CI), plotted relative to the non-inferiority margins (dotted lines) of 10 units for knee pain. The grey area represents the non-inferiority zone.

© 2025 Abafita BJ et al. JAMA Network Open.

eReferences

1. Singh A, Aitken D, Moonaz S, et al. A Randomised Controlled Trial of YOGa and Strengthening Exercise for Knee OsteoArthritis: Protocol for a Comparative Effectiveness Trial (YOGA Trial). *Journal of Functional Morphology and Kinesiology*. 2022;7(4):84. doi:10.3390/jfmk7040084
2. Australia ESS. Adult Pre-Exercise Screening

System (APSS). Accessed November, 2020. https://www.essa.org.au/Public/Public/ABOUT_ESSA/Pre-Exercise_Screening_Systems.aspx
3. Moonaz SH, Bingham CO, Wissow L, Bartlett SJ. Yoga in Sedentary Adults with Arthritis: Effects of a Randomized Controlled Pragmatic Trial. *The Journal of Rheumatology*. 2015;42(7):1194-1202. doi:10.3899/jrheum.141129
4. Steffany Moonaz PR, Susan J. Bartlett, PhD, and Clifton O. Bingham III, MD. Yoga for Arthritis. Accessed 03, 2023. https://www.hopkinsarthritis.org/patient-corner/disease-management/yoga-for-arthritis/
5. Bennell KL, Ahamed Y, Jull G, et al. Physical Therapist–Delivered Pain Coping Skills Training and Exercise for Knee Osteoarthritis: Randomized Controlled Trial. *Arthritis Care & Research*. 2016;68(5):590-602. doi:10.1002/acr.22744
6. Bellamy N, Buchanan WW, Goldsmith CH, Campbell J, Stitt LW. Validation study of WOMAC: a health status instrument for measuring clinically important patient relevant outcomes to antirheumatic drug therapy in patients with osteoarthritis of the hip or knee. *J Rheumatol*. Dec 1988;15(12):1833-40.
7. Hawker GA, Mian S, Kendzerska T, French M. Measures of adult pain: Visual Analog Scale for Pain (VAS Pain), Numeric Rating Scale for Pain (NRS Pain), McGill Pain Questionnaire (MPQ), Short-Form McGill Pain Questionnaire (SF-MPQ), Chronic Pain Grade Scale (CPGS), Short Form-36 Bodily Pain Scale (SF. *Arthritis Care & Research*. 2011;63(S11):S240-S252. doi:10.1002/acr.20543
8. Dobson F, Hinman RS, Roos EM, et al. OARSI recommended performance-based tests to assess physical function in people diagnosed with hip or knee osteoarthritis. *Osteoarthritis and Cartilage*. 2013;21(8):1042-1052. doi:10.1016/j.joca.2013.05.002
9. Wright AA, Cook CE, Baxter GD, Dockerty JD, Abbott JH. A Comparison of 3 Methodological Approaches to Defining Major Clinically Important Improvement of 4 Performance Measures in Patients With Hip Osteoarthritis. *Journal of Orthopaedic & Sports Physical Therapy*. 2011;41(5):319-327. doi:10.2519/jospt.2011.3515
10. Löwe B, Kroenke K, Herzog W, Gräfe K. Measuring depression outcome with a brief self-report instrument: sensitivity to change of the Patient Health Questionnaire (PHQ-9). *J Affect Disord*. Jul 2004;81(1):61-6. doi:10.1016/s0165-0327(03)00198-8
11. Pham T, Van Der Heijde D, Altman RD, et al. OMERACT-OARSI Initiative: Osteoarthritis Research Society International set of responder criteria for osteoarthritis clinical trials revisited. *Osteoarthritis and Cartilage*. 2004;12(5):389-399. doi:10.1016/j.joca.2004.02.001
12. Freynhagen R, Baron R, Gockel U, Tölle TR. pain<i>DETECT</i>: a new screening questionnaire to identify neuropathic components in patients with back pain. *Current Medical Research and Opinion*. 2006;22(10):1911-1920. doi:10.1185/030079906x132488

eAppendix. Yoga and Strengthening Exercises: Intervention Description/Manual

© 2025 Abafita BJ et al. JAMA Network Open.

Yoga and Strengthening Exercises: Intervention Description/Manual

© 2025 Abafita BJ et al. *JAMA Network Open*.

Contents

Group yoga program introduction 5

Standing Asanas* 6

Warrior 2 (Class 1) 6

Side Angle Pose (Class 5) 7

Reverse Warrior (Class 7) 8

Triangle (Class 3) 9

Tree (Class 1) 10

King Dancer (Class 3) 11

Warrior 3 (Class 5) 12

Eagle (Class 7) 13

Goddess (Class 8) 14

Half-Moon (Class 11) 15

Floor Asanas* 16

Head to Knee (Class 1) 16

Seated Forward Fold (Class 5) 17

Tailor's Pose (Class 6) 18

Wide Angle Forward Bend (Class 11) 19

Spinal Twist (Class 1) 20

Lying Extended Leg Pose (Class 4) 21

Lying Spinal Twist (Class 4) 22

Bridge (Class 5) 23

Locust/Bow (Class 8) 24

Child/Puppy (Class 8) 25

Restorative Pose Options (Class 12) 26

Savasana 29

- Tense and Release 29
- Progressive Body Scan 29
- Breath and Mind relaxation 29

Closing 30

Chant 30

Meditation 30

Group strengthening program introduction. 31

Exercise dosage and progression 31

Warm up 5 mins 35

Exercise 1: Forwards/backwards exercise 36

Level 1: Sliding 36

© 2025 Abafita BJ et al. *JAMA Network Open*.

Exercise 1: Forwards/backwards exercise 37
Level 2: Sliding with Thera-band... 37
Exercise 1: Forwards/backwards exercise 38
Level 3: Stepping 38
Exercise 1: Forwards/backwards exercise 39
Level 4: Stepping with Thera-band... 39
Exercise 2: Sideways exercise 40
Level 1: Sliding 40
Exercise 2: Sideways exercise 41
Level 2: Sliding with Thera-band... 41
Exercise 2: Sideways exercise 42
Level 3: Sliding with Thera-band and foam... 42
Exercise 2: Sideways exercise 43
Level 4: Sliding with Thera-band and foam and eyes closed 43
Exercise 3: Hip muscle strengthening... 44
Level 1: Side leg raises in standing 44
Exercise 3: Hip muscle strengthening... 45
Level 2: Crab walking + red Thera-band 45
Exercise 3: Hip muscle strengthening... 46
Level 3: Wall push 46
Exercise 3: Hip muscle strengthening... 47
Level 4: Wall push with deeper knee bending 47
Exercise 4: Knee muscle strengthening 48
Level 1: Chair stands... 48
Exercise 4: Knee muscle strengthening 49
Level 2: Chair stands with more weight on arthritis leg 49
Exercise 4: Knee muscle strengthening 50
Level 3: Wall squats... 50
Exercise 4: Knee muscle strengthening 51
Level 4: Wall squats with more weight on arthritis leg 51
Exercise 5: Step-ups... 52
Level 1: Step-ups... 52
Exercise 5: Step-ups... 53
Level 2: Step-ups with weight... 53
Exercise 5: Step-ups... 54
Level 3: Forward touch downs... 54

© 2025 Abafita BJ et al. *JAMA Network Open*.

Exercise 5: Step-ups....55
Level 4: Forward touch downs with weight....55
Exercise 6: Hamstring strengthening....56
Level 1: Seated knee flexion....56
Exercise 6: Hamstring strengthening....57
Level 2: Hamstring curls with resistance....57
Exercise 6: Hamstring strengthening....58
Level 3: Hip Extension with knee straight....58
Exercise 6: Hamstring strengthening....59
Level 4: Hip extension knee bent....59
Exercise 7: Calf strengthening....60
Level 1: Double calf raises....60
Exercise 7: Calf strengthening....61
Level 2: Single calf raises....61
Exercise 7: Calf strengthening....62
Level 3: Double calf raises over edge of step....62
Exercise 7: Calf strengthening....63
Level 4: Single calf raises over edge of step....63
Exercise 8: Seated Knee Extension....64

© 2025 Abafita BJ et al. *JAMA Network Open*.

Group yoga program introduction

Each class will begin with Chanting. This will be followed by 10 mins of warm-up session which will include body scan and focus, pranayama/chanting, and joint mobilization. This will be followed by 10 mins of sun salutations. Next will be 10 mins each of variable standing asanas and floor asanas. This will be followed by 10 mins of savasana comprising, tense and release, progressive body scan, breath and mind relaxation. Classes will end with 5 mins of asanas comprising, introduce awareness and movement, side lying, return to sitting, chant and meditation.

Yoga pose	Duration
Centering and Chanting	5 mins

Centering

This includes a focus on postural alignment, breath awareness, and an inward focus. An intention is set to foster mindfulness and disregard distractions or concerns outside of yoga practice.

Chanting

The chant "Hari Om" is a Sanskrit phrase that is intended to raise vibrational energy and erase suffering. This can be thought of in a universal sense, but can also apply to individual practitioners who suffer from chronic pain.

Yoga pose	Duration
Warm-up	10 mins

- **Body scan and focus**
- **Pranayama**
- **Joint mobilization**

Body Scan

This practice fosters mindful observation of the state of the body and equanimity regarding the relationship to the body as it changes. It also ensures safety in yoga practice by paying attention to the body's experience and sensation.

Pranayama

Pranayama is controlled breath. While there will be attention to coordination of breath and movement throughout the practice, this beginning pranayama focuses on deep abdominal breathing to reduce the stress response and calm the autonomic nervous system.

Joint Mobilization

This sequence briefly mobilizes all major synovial joints of the body to reduce stiffness and increase mobility in preparation for larger movements.

Yoga pose	Duration
Sun Salutations	10 mins

Description

The Sun Salutation sequence includes 10 poses that will each be taught individually in the first class. Fluid movement between these 10 poses makes up the Sun Salutation, which will be practiced on both sides with individualized modifications during every class. Students may use the wall, a chair, or other props to safely execute the sequence without discomfort. The Sun

© 2025 Abafita BJ et al. *JAMA Network Open*.

Salutations will take longer to execute in early classes, but will be reduced to 10 minutes later in the intervention as students become more facile with it.

Yoga pose	Duration
**Standing Asanas***	10 mins

Warrior 2 (Class 1)
Directions
This is the first standing pose to be introduced and feels awkward to many students at the beginning. There are a lot of postural considerations, so take your time experimenting with variations. The stance should be wider than most would imagine. After stepping wide on the mat, hold the arms straight out, with wrists and shoulders in one line. Look at the feet to ensure they are positioned under the palms. You may have to step wider to accomplish this, which will be important for knee alignment later in the pose. Toes on the front foot turn 90 degrees to face front, and toes on the back foot turn 45 degrees to face the corner. Many will not turn this back foot in enough, which will hinder the ability to orient the hips properly. If you have limitations in the ankle, due to fused bones, deformity, or joint pain, put a wedge or block under the back heel. Turn the head to gaze over the front shoulder, being sure not to lower the back arm. Bend the front knee. Be sure the knee does not go past the ankle. If it does, widen the stance. Shoulders and hips are square to the long edge of the mat, even though the gaze is to the front. Relax the shoulders away from the ears and lengthen the fingertips with energy reaching out to both sides. It is an option to perform this posture with the support of a chair.

© 2025 Abafita BJ et al. *JAMA Network Open*.

Side Angle Pose (Class 5)
Directions
Starting in Warrior II, bring the front elbow to rest on the bent knee. Be sure that the knee is stacked over the ankle and not past. To go deeper, you might try lowering that hand to a block, keeping one shoulder stacked over the other. The other arm can extend alongside the body, into cactus, or up alongside the ear. You can also bring the hand to rest on the upper hip. The goal is one continuous diagonal line from the back foot to the extended arm or crown of the head. You can gaze past the extended fingers, past the upper shoulder, or straight ahead. Watch for tension or condensing in the lower shoulder as you lean into the supporting elbow. The hips and shoulders should be in one plane, as though laying against a wall. Release any tension in the hand and fingers of the extended arm. Focus on healthy alignment rather than trying to achieve a certain shape or depth in the pose.

© 2025 Abafita BJ et al. *JAMA Network Open*.

Reverse Warrior (Class 7)
Directions
From the Warrior II stance (either standing or seated), the back hand comes to rest on the outside of the back leg. It is not important how far down the leg this is. The hand can also rest on the back hip if that is more comfortable. Avoid too much pressure on the back leg in order to protect the knee.
The front arm reaches overhead in an arc, straight up, or in cactus. The front hand can also rest on the shoulder. Gaze is traditionally up into the palm, but take care to protect the neck. It is fine to look forward if that feels better.

Bend the knee deeply, as in Warrior I or II, being sure that the knee does not extend past the ankle and that weight is distributed evenly through the front foot. A wedge or other prop can be placed under the back heel or outside edge of the foot as needed.

© 2025 Abafita BJ et al. *JAMA Network Open*.

Triangle (Class 3)
Directions
Begin in the same stance as Warrior II, with the front knee straight but not locked. Stand tall with arms extended to the sides and shift the torso forward toward the front leg, reaching forward with the front arm. From there, begin tilting the torso sideways so that the hand naturally reaches a resting place on your leg, a block, or a chair.

Make sure you are not reaching too far down and compromising the alignment of the torso; keep one shoulder stacked over the other. Think about opening the front of that upper shoulder with the arm reaching toward the ceiling. The hand can also be placed on the upper hip with the elbow reaching upward.

The gaze is traditionally up toward the open palm but can be straight ahead or down for more comfort in the neck.

© 2025 Abafita BJ et al. *JAMA Network Open*.

Tree (Class 1)
Directions
Standing on both feet, perhaps with the support of a chair or wall for balance, begin to shift the weight toward the left foot, coming to the ball of the foot on the right side. Rotate the right leg out from the hip and bring the right foot close to the left ankle. You can stay here, or try bringing the right foot to rest on the inside of the left ankle. Be sure toes point straight down, not forward.

You might want to try sliding the right foot up to the left calf, or even using the right hand to bring the right foot up to the left thigh. Be sure to avoid putting the foot directly on the knee. The right leg acts like a buttress for a building to keep you steady, with pressure of foot and leg against each other creating resistance to maintain the pose.

Keep length in the left side body without sinking into the left hip. Hands can be on the hips, at the heart, in cactus, or overhead. Keep shoulders relaxing down. If you are holding onto the foot, be sure not to hunch upper body forward but to keep a vertical spine. The gaze is up and out, on a focal point of your choice. Breathe deeply and repeat on the other side.

© 2025 Abafita BJ et al. *JAMA Network Open*.

King Dancer (Class 3)
Directions
Most students will want to use a strap or chair for this pose.
To use a strap, begin standing by the wall or a chair for support and wrap the strap around the front of the ankle, holding onto it with one hand or looping it over the same shoulder. Alternatively, place the foot or knee on a chair behind you. Hip/knee/ankle should be in one plane (so the knee is not torqued) and hips are square to the front. If feeling stable, the other arm can be raised overhead or to a cactus position. For greater challenge, the pose can be tipped forward with one line from fingertips to knee. There is a tendency for the angle of the hip to decrease or the back to round when extending forward. It is more important to keep a long spine than to tip forward. The supporting knee should be soft with weight evenly distributed on the whole foot.

A seated version of the pose begins in Seated Mounted with one knee lowering to the side of the chair. Be sure to avoid a pulling sensation in the front of the knee, so the stretch is felt more in the middle of the thigh and/or hip flexors.

© 2025 Abafita BJ et al. *JAMA Network Open*.

Warrior 3 (Class 5)
Directions
You may want to begin preparations for Warrior III at a chair or wall for support. This is a challenging pose, but it can be modified for broader accessibility. Begin by shifting weight into the left foot and extending the right foot back so that just the toes are touching the floor behind you. You can try lifting the right hand off of the chair/wall and extending it overhead or to cactus position. This may be your Warrior III.

Consider a long line of energy extending from the left leg to the right arm and maintain the alignment of that line. If you like, you can begin lifting the left toes and tipping the torso forward. It is more important to maintain the line from fingertips to the heel of the extended leg than it is to tip forward. In fact, you might stay relatively upright, which is fine.
Be sure that as you go deeper into the pose, the supporting knee remains soft with weight distributed through the whole foot. Gaze can be down or straight ahead. Gently return to Mountain and repeat on the other side.

© 2025 Abafita BJ et al. *JAMA Network Open*.

Eagle (Class 7)
Directions
One knee crosses over the other, bringing them as close as possible, which is easier with a deep bend in knees and hips or seated in a chair. It can also be done against a wall for stability and support.
For those with arthritis in hips, knees, ankles, or feet, it is not necessary to try bringing the foot around the back of the calf, as is done traditionally. It may be more comfortable to rest the foot on a block or other prop.

The elbows cross in the opposite manner. It is fine if the elbows don't touch. More important is the idea of opposing twists. The lower body twists and crosses in one direction, while the upper body twists and crosses in the other direction, bringing the body back to center.
Whether or not the palms are touching, fingers should be elongated and pointed upward, aiming to keep the eyes between the hands or the hands between the eyes.

© 2025 Abafita BJ et al. *JAMA Network Open*.

Goddess (Class 8)
Stand with the legs wide, as with Wide Angle Forward Bend. Place hands on the hips with the shoulders back and down. Rotate the legs outward from the hip so the toes are pointing slightly away from forward. Ensure adequate core support by engaging the abdominal muscles slightly while continuing to breath freely. Shoulders should be aligned directly over the hips with a lengthened spine. Bend the knees being sure to keep weight on the outside of the feet (without rolling in on the arch) and the knees bending directly over the feet. The bend can be very slight. Ensure that the knees do not bend past the toes. If there is sufficient stability, arms may be placed in "cactus" position with fingers spread wide apart and palms facing forward.

© 2025 Abafita BJ et al. *JAMA Network Open*.

Half-Moon (Class 11)

Directions
This is a fairly advanced pose and is not introduced until late in the session, perhaps moving from a standing pose or other balance. I recommend using a block (at any height) or a chair to support the pose without reaching for the floor.

There is an intention toward creating one line from head to the heel of the extended leg. The other hand can be on the hip or reaching toward the ceiling within the limits of comfort for the shoulder.
Gaze can be upward toward an extended arm, straight forward, or down toward the floor, depending on the comfort of the neck. This is best done against a wall at first, lying back against the wall to feel the expansiveness of the pose. It can then be replicated just an inch away from the wall to challenge balance, if desired.

*All poses are highly adapted to individual needs and abilities using variations and props. Poses as executed in the intervention may not closely resemble traditional images or drawings.

© 2025 Abafita BJ et al. *JAMA Network Open*.

Yoga pose	Duration
**Floor Asanas***	10 mins

Head to Knee (Class 1)
Directions
Starting in Staff pose on the floor or a chair, bend one knee toward the chest, planting the foot on the floor or chair. Let the knee open out to the side with the sole of the foot touching the inside of the opposite leg (like in Tree). You may want some support, like a block or blanket, under the knee.
Knee and toes of the straight leg point up toward the ceiling. Sitting up tall here might be your full pose. If you have room to hinge forward, keep a long spine as you walk the hands down the sides of the extended leg. Work to center the sternum over the thigh so that the shoulders are somewhat even. Taking a look at the elbows can assist with this. They should be the same distance from the floor.
A strap can be wrapped under the sole of the foot, holding onto the strap with both hands or looping the arms through the strap. Keep a long spine unless folding to 45 degrees, at which point you can release forward and surrender.

© 2025 Abafita BJ et al. *JAMA Network Open*.

Seated Forward Fold (Class 5)

Directions

Start in Staff Pose, sitting up on a blanket or bending knees if the hamstrings are tight. Arms reach overhead and torso leans forward from the hips. A strap can be wrapped under the soles of the feet, holding on with both hands to pull further into the pose.

If the hamstrings are loose and the student does not have difficulty grasping, the first two fingers can be wrapped around the big toe. Elbows open out to the side as the arms bend to draw the torso closer to the legs.

For those with difficulty grasping, the arms can just rest on or beside the legs.

© 2025 Abafita BJ et al. *JAMA Network Open*.

Tailor’s Pose (Class 6)
Directions
Knees are bent out to the sides, soles of the feet touching. Legs are pulled in as close to the body as possible, which may not be very far. Again, sitting up on a blanket or in a chair may help with being able to lengthen the spine.

Keep the spine lengthened, bringing the sternum closer to the feet. Elbows can lower toward the knees, applying a bit of pressure to increase the stretch if available. If uncomfortable, blocks can be placed under the knees for support.

Gaze is out past the feet, shoulders are relaxed away from the ears. If the torso moves past 45 degrees, you can allow the back to round slightly as you release forward into the pose. At this point, you may want to extend the arms forward or relax them to the sides.

© 2025 Abafita BJ et al. *JAMA Network Open*.

Wide Angle Forward Bend (Class 11)
Directions
Start with legs as wide as they would be for Warrior II, but with both pointing straight ahead. Take your arms out to the sides for reference and consider bringing the feet as wide as the hands . Internally rotate the legs slightly (heels out, toes in). Hands are placed on the hips, with shoulder blades drawn together and down. Fold forward at the hips, trying not to lean back into the heels. If you feel unstable with the hands on the hips, they can be placed on floor, block, or chair.

© 2025 Abafita BJ et al. *JAMA Network Open*.

Spinal Twist (Class 1)
Directions

Start in Staff pose, either on the floor or in a chair, perhaps sitting on a prop (blanket, pillow, bolster). On the floor, pull one knee in as if preparing for Head-to-Knee pose. You might choose to leave the foot planted on the floor there, or lift and cross it over to the outside of the extended leg.

In either case, imagine making a footprint on the floor. Drawing the foot in close to the body may help you sit up taller, as long as it feels okay for the hip and knee.

In the floor version, wrap the opposite arm around the bent knee, hugging it toward the body as you sit tall and keep sitz bones planted evenly on the floor. In a chair, you may choose to keep the feet firmly planted or to cross one leg over the other. Sit up tall and begin to twist from the base of the spine toward the crossed leg, looking gently over your shoulder.

© 2025 Abafita BJ et al. *JAMA Network Open*.

Lying Extended Leg Pose (Class 4)
Directions

Lying on your back, pull one leg into the chest, holding behind the knee instead of on the shin. Allow the extended leg to relax on the floor. Alternatively, you could bring the sole of that foot to the floor for stability and ease in the lower back, with the knee pointing up toward the ceiling.

Most students will want to use a strap, wrapping it around the sole of the foot to extend the leg upward toward the ceiling. You can also hold the back of the leg as it extends. The head can be lifted briefly toward the knee, pulling in with the abdominal muscles.

Continue by bringing both ends of the strap into one hand on the side of the lifted leg. The other hand should be placed on the hip of the lower leg or alongside the body. The upper leg then opens out to the side, being sure to keep both hips on the floor. The leg will most likely not go to the floor, and that's okay because it can rest on a block.

If the hips are stable, the other arm can be extended out to the side, and possibly the head can turn in that direction. Switching the strap to the other hand, you can then move into an extended leg version of Lying Spinal Twist (see next page).

© 2025 Abafita BJ et al. *JAMA Network Open*.

Lying Spinal Twist (Class 4)
Directions

With or without a strap and moving from Extended Leg pose, the leg can be brought across the body to the floor on the other side. The other arm extends out to the side, and the head turns in that direction.

Although there is some active stretching of the hamstrings by holding the strap or leg, the spinal twist is passive. The leg should be relaxed on the floor as the opposite shoulder reaches toward the floor. It is fine if the shoulder doesn't touch, and the hip of the top leg should be off the floor so the twist extends through the whole spine.
The spinal twist can also occur after Bridge, starting with both knees bent into the chest and dropping the knees over to one side. Both arms are extended out to the sides, and the head turns away from the knees.

In any spinal twist, if arthritis in the spine is hurting, the legs can rest on a block or bolster to decrease the intensity of the pose. If turning the head is bothersome, that step can be left out.

© 2025 Abafita BJ et al. *JAMA Network Open*.

Bridge (Class 5)
Directions
Lying on the back, soles of the feet are placed on the floor, about one foot from the hips. Feet should be hip-distance apart, and knees pointing straight upward. Arms are extended alongside the body. Pressing into the feet, hips are lifted off the floor.

You may not have enough strength to lift the hips at first, which is fine. Simply engage the muscles as if you were going to lift, and eventually, you will build up enough strength for that to happen. There is as much benefit in doing this as there would be for someone who could lift the hips fully.
It is important to continue breathing and not strain to accomplish the movement. There is a tendency for the knee positioning to be compromised, and for the angle of the feet to change as you move into the pose. Toes should point straight forward.

You can also use a strap to keep the knees from opening outward. For more of a challenge (or when doing several rounds), you can try reaching the hands for the heels and/or lifting one leg at a time toward the ceiling.

© 2025 Abafita BJ et al. *JAMA Network Open*.

Locust/Bow (Class 8)
Directions

Begin lying on the abdomen, arms alongside the body. Rest the forehead on the floor or a folded blanket for a few breaths. Try rocking the body side to side, tucking the hands under the thighs, palms facing up.

You may be able to tuck the elbows inside the hip bones, but if neither of these positions is comfortable, the arms can be to the sides of the body. Having the palms face down may provide more stability.

For half locust, lift one leg, keeping the hips level. The forehead or chin can remain on the floor to support the cervical spine. Switch to the other leg.
If you want to try full locust, bring both legs together and lift simultaneously. This is challenging, and the legs may not lift very high. Be sure to keep the knees lengthened since there is a tendency to bend the knees for greater height.

Once again, it is not about the height of the pose but the intention, muscle engagement, breath, and integrity of the alignment. Focus on the engagement of the core muscles and be sure there is no aggravation of the low back or neck.

© 2025 Abafita BJ et al. *JAMA Network Open*.

Child/Puppy (Class 8)
Directions
Child's pose is supposed to be grounding and restful, but that may not be true for those with arthritis in the knees or hips. A variety of variations are offered here to bring ease and grounding into the pose.
You might start standing on knees, just like for Camel pose, perhaps with cushioning under the knees. Sit back on heels, a block between the feet, or a blanket. Bring forehead to the floor with arms extended out in front or alongside the body.

If all of these poses are uncomfortable, hips can be raised to decrease knee angle. Alternatively, try sitting on a chair and folding forward to support the forehead with cushioning on another chair.
Props can be used creatively to soften this pose, so experiment with options until you find what works for you.

© 2025 Abafita BJ et al. *JAMA Network Open*.

Restorative Pose Options (Class 12)

Directions

WIDE ANGLE FORWARD BEND – Sitting on the floor, perhaps on a prop, open the legs out to the sides. Place bolsters, blocks, or other props in front of you. Hinge at the hips and lower forward, relaxing the forehead or torso on those props.

This can also be done seated in a chair, with another chair in front of you to rest on. Alternatively, you can use a standing version that isn't truly restorative but may still be grounding and relaxing. For that version, start with the feet wide and then pigeon-toe the feet slightly, bringing heels out and toes in, about 20 degrees.

Bring hands to the hips and hinge forward, keeping a lengthened spine. Hands can be placed on a chair, block, or the floor. The head hangs without tension in the neck or shoulders.
LYING SUPPORTED SPINAL TWIST – Lying on your back with knees curled into the chest or flat on the floor, open arms out to both sides. Allow the knees to fall to one side while the head turns to the other.

Knees should rest on blankets and/or blocks for greatest comfort and ease. A blanket can also be placed under the opposite shoulder.

© 2025 Abafita BJ et al. *JAMA Network Open*.

Restorative continued
Directions
SUPPORTED CHILD – Standing on knees, with knees angled out and toes angled in, use cushioning under the knees as needed. A blanket can be placed behind the knees to decrease the angle.
You can also place a block or blanket between the legs to sit on, although some may find this uncomfortable. A stack of blankets should be in front of the body so that the upper body rests on that stack. The number of blankets necessary may vary considerably, and it may be necessary to place blocks under the blankets for additional height.

If none of these positions is suitable, hips can remain high while the upper body rests at a lower level.
SUPPORTED FORWARD BEND – Sitting in Staff pose, on blankets if necessary for a vertical spine. Place a stack of blankets or bolsters across the thighs, high enough to rest arms and head. Lean over the blankets, resting the chin on the hands or turning the head to one side. Alternatively, rest the forehead on a chair in front of you.

© 2025 Abafita BJ et al. *JAMA Network Open*.

Restorative Continued
SUPPORTED RECLINING BOUND ANGLE
Sit in Tailor's pose with the soles of the feet touching, knees supported as needed. You might try wrapping a strap around the outside of the feet, holding onto one side of the strap with each hand to slowly lower back.

Lean back over a stack of blankets to recline. Blankets should be folded narrowly so that the shoulders fall out to either side.
You can continue holding onto the strap (with little muscle effort) for more of a stretch in the hips, or allow the arms to open out to the sides for more chest opening.

*All poses are highly adapted to individual needs and abilities using variations and props. Poses as executed in the intervention may not closely resemble traditional images or drawings.

© 2025 Abafita BJ et al. *JAMA Network Open*.

Yoga pose	Duration
**Savasana**	10 mins

- **Tense and Release**
- **Progressive Body Scan**
- **Breath and Mind relaxation**

<u>Savasana</u>

Savasana (Corpse pose) is traditionally done lying on the back without any propping. For a supported Corpse, you can experiment with what will be most comfortable, relaxing and sustainable for the duration of the progressive relaxation. Because Corpse lasts several minutes and the focus should be on restoration and relaxation, it is important to find a position without strain. You may want to put a rolled blanket under the knees, rest the lower legs on a chair, place a narrowly folded blanket along the spine, support the lumbar region, or place something under the head. These are a few examples but take your time and make adjustments until you feel satisfied with the position. Even then, it is okay to change position during Savasana if it is no longer comfortable or is hindering relaxation.

© 2025 Abafita BJ et al. *JAMA Network Open*.

Yoga pose	Duration
**Closing**	5 mins

- **Introduce awareness and movement**
- **Side lying**
- **Return to sitting**
- **Chanting**
- **Meditation**

Chant
The closing chant will use the phrase "Om Shanti" which is a chant for peace. This can be thought of as universal peace or as individual peace within.

Meditation
Yoga programs often include brief periods of meditation before or after the asana practice. During these times, some instructors give guidance on how to approach meditation. During meditation one remains quiet and focus the mind, relieving it of the unnecessary clutter of trivial thoughts that stream in and out during the day. This discipline of the mind is said to provide relief from the day's stresses. Meditation can have any focus, such as the breath, an image, an idea or affirmation, a sound, or a personal prayer. In this intervention, we will allow each individual participant to choose a focus that feels comfortable and meaningful to them, with the goal of returning to that focus when noticing distractions.

© 2025 Abafita BJ et al. *JAMA Network Open*.

Group strengthening program introduction.
Group sessions will be held 3 times per week for 12 weeks. Each group session will be 60 minutes and will comprise:

1. Assessment – 5 mins
2. Warm up – 5 mins
3. Lower limb strengthening exercises run as a circuit class – 45 mins
4. Cool down – 5 mins

Information – At the start of the first session before commencing the program (and reinforced in subsequent sessions), the instructor should advise patients that some degree of knee discomfort or pain is to be expected with the exercises and that this generally reduces after the first few weeks, following which they should expect to see improvements in their knee pain and function. However, exercises should be performed within tolerable levels of pain. Any pain should also subside to usual levels by the next day with no increase in swelling following the exercise session. Patients should also be informed that when they start new exercises, there can be some soreness in the muscles which indicates that the muscles have been working. This is normal and will subside over a few days.

Assessment – At the start of each session, the instructor should ask the group how they found the previous exercise session and whether anyone had experienced any issues. This information can be used to modify the program for individuals.

Patient safety – This includes:

1. Asking patients to wear appropriate clothing to exercise
2. Bringing a drink bottle and towel
3. Letting the instructor know if they feel light-headed or dizzy during the exercises
4. Using hand support via a wall or chair for standing exercises if needed
5. Explaining that the exercises should be performed smoothly and slowly with quality of the movement emphasised.

Exercise dosage and progression
In the first week, the emphasis should be on learning the movements and using lower resistance. One set of exercises can be performed. The number of sets can be increased up to 2 and 3 over the weeks. After the first week, patients should be challenged by the exercises. During all exercises, their level of effort should be 5-7 out of 10 on the Modified Borg Rating of Perceived Exertion (RPE) scale shown on the following page.

Table: Modified Rating Perceived Exertion Scale (Day et al J Strength Conditioning Res 2004)

Rating	Descriptor
0	Rest
1	Very, Very Easy
2	Easy
3	Moderate
4	Somewhat Hard
5	Hard
6	-
7	Very Hard

© 2025 Abafita BJ et al. *JAMA Network Open*.

8	-
9	-
10	Maximal

Progression of exercises is an essential component of the program and is determined by the instructor's assessment of the quality of the exercise performance and on the patient's RPE score for each exercise. A recommended progression rate is indicated for each exercise below. This is to be used as a guideline and the instructor may vary this rate of progression for an individual if they feel it is indicated. For example:

1. If the patient is not able to perform the task with good form after 3 weeks, they may remain on that level for another week.
2. If the patient is experiencing increased pain and swelling lasting more than one day after their exercises, they can go back to a previous level or do fewer repetitions of their current level.
3. If the patient is not sufficiently challenged by a level, they may be progressed earlier than indicated in the table below.

Table: Guide to progression for group strengthening exercise program

**Level**	**Dosage**	**Progression(weeks)**
**Warm Up**		
1. Hip Circles	Perform 20 circles in each direction. Switch legs. Progressively increase the size of the circles as you become more flexible.	-
2. Arm Circles	Gently perform 20 circles in each direction.<br>Progressively increase the size of the circles as you become more flexible.	-
3. High Stepping	Perform "high-stepping" five times on each leg as you walk forward.	-
4. Heel-Toe Walk	Perform five times on each leg.	-
5. Marching on spot or walking around the room	Perform for 2 minutes	-
**Exercise 1. Forwards/ backwards**		
1. Sliding	1-3 sets of 10 with break of 30-60 seconds<br>between sets	Weeks 1, 2 and 3
2. Sliding with Thera-band	2-3 sets of 10 with break of 30-60 seconds<br>between sets	Weeks 4, 5 and 6
3. Stepping	2-3 sets of 10 with break of 30-60 seconds<br>between sets	Weeks 7, 8 and 9

© 2025 Abafita BJ et al. *JAMA Network Open*.

4. Stepping with Thera-band	2-3 sets of 10 with break of 30-60 seconds between sets	Weeks 10, 11 and 12.
**Exercise 2. Sideways exercise**		
1. Sliding	1-3 sets of 10 with break of 30-60 seconds between sets	Weeks 1, 2 and 3
2. Sliding with Thera-band	2-3 sets of 10 with break of 30-60 seconds between sets	Weeks 4, 5 and 6
3. Sliding with Thera-band and foam	2-3 sets of 10 with break of 30-60 seconds between sets	Weeks 7, 8 and 9
4. Sliding with Thera-band, foam and eyes closed	2-3 sets of 10 with break of 30-60 seconds between sets	Weeks 10, 11 and 12.
**Exercise 3. Hip muscle strengthening**		
1. Side leg raises in standing	1-3 sets of 10 with break of 30-60 seconds between sets	Weeks 1, 2 and 3
2. Crab walk	Total of 30 steps in each direction. [May do all 30 before changing direction or may do in smaller groups depending on available space	Weeks 4, 5 and 6
3. Wall push	20 second holds with short break between efforts. Two sets of 5 with break of 30-60 seconds between sets.	Weeks 7, 8 and 9
4. Wall push with knee bending	Short break between efforts. Two sets of 5 with break of 30-60 seconds between sets.	Weeks 10, 11 and 12.
**Exercise 4. Knee muscle strengthening**		
1. Chair stands	1-3 sets of 10 with break of 30-60 seconds between sets	Weeks 1, 2 and 3
2. Chair stands with more weight on arthritis leg	2-3 sets of 10 with break of 30-60 seconds between sets	Weeks 4, 5 and 6
3. Wall squats	2-3 sets of 10 with break of 30-60 seconds between sets	Weeks 7, 8 and 9
4. Wall squats with more weight on arthritis leg /	2-3 sets of 10 with break of 30-60 seconds between sets	Weeks 10, 11 and 12.

© 2025 Abafita BJ et al. *JAMA Network Open*.

deeper angle		
**Exercise 5. Step-ups**		
1. Step-ups	1-3 sets of 10 with break of 30-60 seconds between sets	Weeks 1, 2 and 3
2. Step-ups with 2kg weight	2-3 sets of 10 with break of 30-60 seconds between sets	Weeks 4, 5 and 6
3. Forward touch downs	2-3 sets of 10 with break of 30-60 seconds between sets	Weeks 7, 8 and 9
4. Forward touch downs with 2kg weight	2-3 sets of 10 with break of 30-60 seconds between sets	Weeks 10, 11 and 12.
**Exercise 6. Hamstring strengthening**		
1. Seated knee flexion	1-3 sets of 10 with break of 30-60 seconds between sets	Weeks 1, 2 and 3
2. Hamstring curls with resistance	2-3 sets of 10 with break of 30-60 seconds between sets	Weeks 4, 5 and 6
3. Hip Extension with knee straight	2-3 sets of 10 with break of 30-60 seconds between sets	Weeks 7, 8 and 9
4. Hip Extension with knee bent	2-3 sets of 10 with break of 30-60 seconds between sets	Weeks 10, 11 and 12.
**Exercise 7. Calf strengthening**		
1. Double calf raises	1-3 sets of 10 with break of 30-60 seconds between sets	Weeks 1, 2 and 3
2. Single calf raises	2-3 sets of 10 with break of 30-60 seconds between sets	Weeks 4, 5 and 6
3. Double calf raises from edge of step	2-3 sets of 10 with break of 30-60 seconds between sets	Weeks 7, 8 and 9
4. Single calf raises from edge of step	2-3 sets of 10 with break of 30-60 seconds between sets	Weeks 10, 11 and 12.
**Exercise 8. Seated Knee Extension**		
1. Seated knee extension with resistance	1-3 sets of 10 with break of 30-60 seconds between sets	Weeks 1- 12

© 2025 Abafita BJ et al. *JAMA Network Open*.

Cool Down		
Hamstring stretch	3 x 30 second hold each leg	
Calf stretch	3 x 30 second hold each leg	
Quads stretch	3 x 30 second hold each leg	

Warm up 5 mins
1. Hip Circles
- Stand on one leg, using a wall or chair for support, and gently swing the opposite leg in circles out to the side. Perform 20 circles in each direction. Switch legs. Progressively increase the size of the circles as you become more flexible.

2. Arm Circles
- Stand with feet shoulder-width apart and hold arms out to the sides, palms down, at shoulder height. Gently perform 20 circles in each direction. Progressively increase the size of the circles as you become more flexible.

3. High-Stepping
- Stand with feet parallel to each other and at shoulder-width apart. Step forward with the left leg and raise the right knee high up toward your chest (use a wall for balance, if needed) and use both hands (or one, if using the other for balance) to pull the knee up farther. Pause and bring right leg back down; repeat with the other side and continue "high-stepping" five times on each leg as you walk forward.

4. Heel-to-Toe Walk
- Stand with feet shoulder-width apart and take a small step forward by placing the heel of the right foot on the ground and rolling forward onto the ball of your foot, rising as high as possible (as if standing on tip-toe), while bringing the left foot forward and stepping in the same heel-to-toe roll. Repeat five times on each leg.

5. Marching on spot or walking around the room
- 2 minutes

Cool Down 5 mins

Stretches – 3 x 30 second hold each leg
Hamstring stretch
Calf stretch
Quads stretch

© 2025 Abafita BJ et al. *JAMA Network Open*.

Exercise 1: Forwards/backwards exercise
Level 1: Sliding
Starting position:
Standing on your arthritis leg with the non study leg on a sliding surface.
Sliding can be achieved by using a towel on smooth flooring or a plastic bag on the foot for carpet. Use hand support for balance.
Exercise:

Slowly slide backwards and forwards with the ‘sliding leg’ while bending and straightening the arthritis leg.

Start with sliding just a few inches forwards and backwards and progress to larger slides as you gain control.

Keep your weight on the arthritis leg.

Concentrate on the alignment of your arthritis leg hip, knee and ankle – **position your knee over your foot** throughout. Do 3 sets of 10 repetitions with a break of 30-60 seconds between sets.

© 2025 Abafita BJ et al. *JAMA Network Open*.

Exercise 1: Forwards/backwards exercise
Level 2: Sliding with Thera-band
<u>Starting position:</u>

Place a loop of Thera-Band around your arthritis knee and the leg of a table. This will provide a pull outwards on your knee that you must resist by aligning your knee over your foot through the whole exercise.

Your non study side foot should be on a sliding surface.

Sliding can be achieved by using a towel on smooth flooring or a plastic bag on the foot for carpet.

<u>Exercise:</u>

Slowly slide backwards and forwards with the 'sliding leg' while bending and straightening the arthritis leg.

Start with sliding just a few inches forwards and backwards and progress to larger slides as you gain control.

Keep your weight on the arthritis leg.

Concentrate on the alignment of your arthritis leg hip, knee and ankle – **position your knee over your foot** against the pull of the Thera-band throughout.

Do 3 sets of 10 repetitions with a break of 30-60 seconds between sets.

© 2025 Abafita BJ et al. *JAMA Network Open*.

Exercise 1: Forwards/backwards exercise
Level 3: Stepping
Starting position:

Standing on your arthritis leg with your non-study leg behind.

Use a hand support for balance.

Exercise:

Instead of sliding forwards and backwards, slowly take a step forwards with your nonstudy leg to touch the floor. Then take a step backwards to the starting position.

Keep your arthritis knee slightly bent and your weight on your arthritis leg through the whole exercise.

Start with a small step and progress to larger steps as you gain control.

Concentrate on the alignment of your arthritis leg hip, knee and ankle – position your knee over your foot throughout.

Do 3 sets of 10 repetitions with a break of 30-60 seconds between sets.

© 2025 Abafita BJ et al. *JAMA Network Open*.

Exercise 1: Forwards/backwards exercise
Level 4: Stepping with Thera-band
<u>Starting position:</u>

Place a loop of Thera-Band around your arthritis knee and the leg of a table. This will provide a pull outwards on your knee that you must resist by aligning your knee over your foot through the whole exercise.

Start standing on your arthritis leg with non-study leg behind.

Exercise:

Slowly, take a step forwards with your non-study leg to touch the floor. Then take a step backwards to the starting position.

Keep your arthritis knee slightly bent and your weight on your arthritis leg through the whole exercise.

Start with a small step and progress to larger steps as you gain control.

Concentrate on the alignment of your arthritis leg hip, knee and ankle – position your knee over your foot against the pull of the Thera-band throughout.

Do 3 sets of 10 repetitions with a break of 30-60 seconds between sets.Use hand support for balance.

© 2025 Abafita BJ et al. *JAMA Network Open*.

Exercise 2: Sideways exercise
Level 1: Sliding
Starting position:

Standing on your arthritis leg with the other leg on a sliding surface.

Sliding can be achieved by using a towel on smooth flooring or a plastic bag on the foot for carpet.

Use hand support for balance.

Exercise:

Slowly slide out sideways with the ‘sliding leg’ while bending the arthritis leg. Then slide back to the starting position.

Start with sliding just a few inches and progress to larger slides as you gain control.

Keep your weight on your arthritis leg through the whole exercise.

Concentrate on the alignment of your arthritis leg hip, knee and ankle – **position your knee over your foot** throughout.

Do 3 sets of 10 repetitions with a break of 30-60 seconds between sets.

© 2025 Abafita BJ et al. *JAMA Network Open*.

Exercise 2: Sideways exercise
Level 2: Sliding with Thera-band
Starting position:

Place a loop of Thera-Band around your arthritis knee and the leg of a table. This will provide a pull outwards on your knee that you must resist by aligning your knee over your foot through the whole exercise.

Standing on your arthritis leg with the other leg on a sliding surface.

Sliding can be achieved by using a towel on smooth flooring or a plastic bag on the foot for carpet.

Exercise:

Slowly slide out sideways with the non-study leg while bending your arthritis knee.

Start with sliding just a few inches and progress to larger slides as you gain control.

Keep your weight on your arthritis leg through the whole exercise.

Concentrate on the alignment of your arthritis leg hip, knee and ankle – position your knee over your foot against the pull of the Thera-band throughout.

Do 3 sets of 10 repetitions with a break of 30-60 seconds between sets.

© 2025 Abafita BJ et al. *JAMA Network Open*.

Exercise 2: Sideways exercise
Level 3: Sliding with Thera-band and foam
Starting position:

Place a loop of Thera-Band around your arthritis knee and the leg of a table. This will provide a pull outwards on your knee that you must resist by aligning your knee over your foot through the whole exercise.

Standing on your arthritis leg on your foam cushion, with your non-study leg on a sliding surface.

Sliding can be achieved by using a towel on smooth flooring or a plastic bag on the foot for carpet.

Exercise:

Slowly slide out sideways with the non-study leg while bending your arthritis knee.

Start with sliding just a few inches and progress to larger slides as you gain control.

Keep your weight on your arthritis leg through the whole exercise.

Concentrate on the alignment of your arthritis leg hip, knee and ankle – position your knee over your foot against the pull of the Thera-band throughout.

Do 3 sets of 10 repetitions with a break of 30-60 seconds between sets.

© 2025 Abafita BJ et al. *JAMA Network Open*.

Exercise 2: Sideways exercise
Level 4: Sliding with Thera-band and foam and eyes closed
Starting position:

Place a loop of Thera-Band around your arthritis knee and the leg of a table. This will provide a pull outwards on your knee that you must resist by aligning your knee over your foot through the whole exercise.

Standing on your arthritis leg on your foam cushion, with your non-study leg on a sliding surface. Sliding can be achieved by using a towel on smooth flooring or a plastic bag on the foot for carpet.

Close your eyes.

Exercise:

Slowly slide out sideways with the non-study leg while bending your arthritis knee. Start with sliding just a few inches and progress to larger slides as you gain control.

Keep your weight on your arthritis leg through the whole exercise.

Concentrate on the alignment of your arthritis leg hip, knee and ankle – position your knee over your foot against the pull of the Thera-band throughout.

Do 3 sets of 10 repetitions with a break of 30-60 seconds between sets.

© 2025 Abafita BJ et al. *JAMA Network Open*.

Exercise 3: Hip muscle strengthening
Level 1: Side leg raises in standing
Starting position:

Use the back of a chair or a wall to provide support.

Keep your back straight and facing forward. Don't twist as this will mean the wrong muscles are being exercised.

Loop your elastic band around your ankles.

Exercise:

Keep your back straight. Try not to tilt to the side.

Keep your knee straight and your toes pointing forward.

Lift your arthritis leg out a small way to the side, leading with the heel.

Hold for 5 seconds and then lower slowly.

"Slowly out, hold, 2, 3, 4, 5, slowly in".

© 2025 Abafita BJ et al. *JAMA Network Open*.

Exercise 3: Hip muscle strengthening
Level 2: Crab walking + red Thera-band
Starting position:

Place a loop of red Thera-Band around both ankles so that there is tension when ankles are separated 10cm. Slightly bend both knees.

For safety, you should stand facing a table, a kitchen bench or a wall which you can reach if you lose balance.

Exercise:

Step sideways against the pull of the Thera-band.

Do not twist or turn your body or legs. Your feet must point forwards while you

are stepping sideways.

Concentrate on the alignment of both your arthritis and non-study legs – position your knee over your foot throughout.

Do a total of 30 steps in each direction. For example, you can do all 30 in one direction around a table before changing direction. Or you can do 5 in one direction along a kitchen bench, change direction and do 5 back to the start and repeat this 6 times to reach your total of 30 in both directions.

© 2025 Abafita BJ et al. *JAMA Network Open*.

Exercise 3: Hip muscle strengthening
Level 3: Wall push
<u>Starting position:</u>

Stand sideways to a wall with non-study leg against the wall.

Slightly bend arthritis knee to about 15-20°.

Lift the non-study leg just off the floor so that hip, thigh and knee are touching the wall.

<u>Exercise:</u>

Push your non-study leg into the wall and hold for 20. Return you foot to the floor and rest for a few seconds.

Concentrate on the alignment of your arthritis leg hip, knee and ankle – position your knee over your foot throughout.

Do 2 sets of 5 repetitions with a break of 30-60 seconds between sets.

© 2025 Abafita BJ et al. *JAMA Network Open*.

Exercise 3: Hip muscle strengthening
Level 4: Wall push with deeper knee bending
Starting position:

Stand sideways to a wall with non-study leg against the wall.

Slightly bend arthritis knee to about 15-20°.

Lift the non-study leg just off the floor so that hip, thigh and knee are touching the wall.

Activity:

Push non-study leg into the wall.

While continuing to push into the wall, slowly bend your arthritis knee to a maximum of 45°.

Straighten your knee and return your foot to the floor and rest for a few seconds.

Concentrate on the alignment of your arthritis leg hip, knee and ankle – position your knee over your foot throughout.

Do 2 sets of 5 repetitions with a break of 30-60 seconds between sets.

© 2025 Abafita BJ et al. *JAMA Network Open*.

Exercise 4: Knee muscle strengthening
Level 1: Chair stands
Starting position:

Sit on a standard height (eg. kitchen) chair, with your feet parallel and hip width apart.

Reach your hands out in front.

Exercise:

Stand up from the chair slowly (count 4 seconds) without using your hands.

Slowly return to sitting (count 4 seconds).

"Up, 2, 3, 4, down, 2, 3, 4"

Concentrate on the alignment of both your arthritis leg and your non-study leg – position your knee over your foot throughout.

Do 3 sets of 10 repetitions with a break of 30-60 seconds between sets.

© 2025 Abafita BJ et al. *JAMA Network Open*.

Exercise 4: Knee muscle strengthening
Level 2: Chair stands with more weight on arthritis leg
Starting position:

Sit on a standard height (eg. kitchen) chair, with your feet hip width apart.

Take more weight on your arthritis leg by either (a) placing your non-study leg further forward, or (b) shifting your feet sideways so your arthritis leg is in front of your body.

Reach your hands out in front.

Exercise:

Stand up from the chair slowly (count 4 seconds) without using your hands.

Slowly return to sitting (count 4 seconds).

"Up, 2, 3, 4, down, 2, 3, 4"

Concentrate on the alignment of your arthritis leg hip, knee and ankle – position your knee over your foot throughout. You must have more weight on your arthritis leg through the whole exercise.

Do 3 sets of 10 repetitions with a break of 30-60 seconds between sets.

© 2025 Abafita BJ et al. *JAMA Network Open*.

Exercise 4: Knee muscle strengthening
Level 3: Wall squats
Starting position:

Stand with your back to a wall, feet 10cm apart and 15cm away from the wall.

You may like a towel behind your hips to help you slide down the wall.

Exercise:

Slide slowly down the wall until your knees are bent about 30°, then slowly slide up again.

"Down, 2, 3, hold, 2, 3, up, 2, 3"

Your knees should stay in line with your feet.

Concentrate on the alignment of both your arthritis and non-study legs – position your knee over your foot throughout.

Do 3 sets of 10 repetitions with a break of 30-60 seconds between sets.

© 2025 Abafita BJ et al. *JAMA Network Open*.

Exercise 4: Knee muscle strengthening
Level 4: Wall squats with more weight on arthritis leg
Starting position:

Stand with your back to a wall, feet 10cm apart and arthritis leg 15cm away from the wall. Your non-study side can be (a) further forward or (b) level with the arthritis leg but with your body shifted over the arthritis leg. You must have more weight on your arthritis leg through the whole exercise.

You may like a towel behind your hips to help you slide down the wall.

Exercises:

Slide slowly down the wall until your arthritis knee is bent about 30°, then slowly slide up again.

"Down, 2, 3, hold, 2, 3, up, 2, 3"

Your knees should stay in line with your feet.

You may use some scales under arthritis leg to check you keep more weight on that side.

Concentrate on the alignment of your arthritis leg hip, knee and ankle – position your knee over your foot throughout.

Do 3 sets of 10 repetitions with a break of 30-60 seconds between sets.

© 2025 Abafita BJ et al. *JAMA Network Open*.

Exercise 5: Step-ups
Level 1: Step-ups
Starting position:

Place your arthritis leg onto a step in front of you.

Use a hand support (back of chair or handrail) for balance.

Exercise:

Step up onto the step slowly, carefully controlling the movement of your arthritis knee.

Just lightly touch your non-study leg to the step, and then step it back down slowly to the start position.

Your weight should be on your arthritis leg through the whole exercise.

Concentrate on the alignment of your arthritis leg hip, knee and ankle – position your knee over your foot throughout.

Do 3 sets of 10 repetitions with a break of 30-60 seconds between sets.

© 2025 Abafita BJ et al. *JAMA Network Open*.

Exercise 5: Step-ups
Level 2: Step-ups with weight
Starting position:

Hold 2kg of weight (a) against your chest, (b) in each hand, (c) in one hand while holding on for balance with the other, or (d) in a backpack.

Place your arthritis leg onto a step in front of you. Weight can be a 2L milk bottle filled (2kg) or half filled (1kg) with water.

Exercise:

Step up onto the step slowly, carefully controlling the movement of your arthritis knee.

Just lightly touch your non-study leg to the step, and then step it back down slowly to the start position.

Your weight should be on your arthritis leg through the whole exercise.

Concentrate on the alignment of your arthritis leg hip, knee and ankle – position your knee over your foot throughout.

Do 3 sets of 10 repetitions with a break of 30-60 seconds between sets.

© 2025 Abafita BJ et al. *JAMA Network Open*.

Exercise 5: Step-ups
Level 3: Forward touch downs
<u>Starting position:</u>

Stand on the step.

Use a hand support (back of chair or handrail) for balance.

<u>Exercise:</u>

Controlling the movement of your arthritis knee, reach your non-study

side towards the floor in front. If you can reach the floor, just touch it lightly. Return to the starting position.

Your weight should be on your arthritis leg through the whole exercise.

Concentrate on the alignment of your arthritis leg hip, knee and ankle – position your knee over your foot throughout.

Do 3 sets of 10 repetitions with a break of 30-60 seconds between sets.

© 2025 Abafita BJ et al. *JAMA Network Open*.

Exercise 5: Step-ups
Level 4: Forward touch downs with weight
<u>Starting position:</u>

Stand on the step.

Hold 2kg of weight (a) against your chest, (b) in each hand, (c) in one hand while holding on for balance with the other, or (d) in a backpack.

Weight can be a 2L milk bottle filled (2kg) or half filled (1kg) with water.

<u>Exercise:</u>

Controlling the movement of your arthritis knee, reach your non-study side towards the floor in front. If you can reach the floor, just touch it lightly. Return to the starting position.

Your weight should be on your arthritis leg through the whole exercise.

Concentrate on the alignment of your arthritis leg hip, knee and ankle – position your knee over your foot throughout.

Do 3 sets of 10 repetitions with a break of 30-60 seconds between sets.

© 2025 Abafita BJ et al. *JAMA Network Open*.

Exercise 6: Hamstring strengthening
Level 1: Seated knee flexion
Starting position:

Sit upright in a chair. Move forward so you are sitting toward the front of the chair (so you have enough room to bend your knee under the chair). Place one end of an elastic band securely around a stable object (e.g. a heavy table leg). Loop the other end around the ankle of your arthritis leg.

Exercise:

Keeping your opposite foot on the floor, pull against the elastic band and bend your knee more. Your foot should move back further, under the chair.

“Bend, hold, 2, 3, 4, 5, return”.

Do 3 sets of 10 repetitions with a break of 30-60 seconds between sets.

© 2025 Abafita BJ et al. *JAMA Network Open*.

Exercise 6: Hamstring strengthening
Level 2: Hamstring curls with resistance
<u>Starting position:</u>

Place one end of an elastic band securely around the ankle of your arthritis leg. Place the other end of the elastic around your opposite foot so you are standing on it.

Stand and lean forward on your forearms over a table.

<u>Exercise:</u>

Slowly bend your arthritis knee so that your heel comes up toward your bottom.

Hold for 5 seconds and lower slowly. “Slowly up, hold, 2, 3, 4, 5, slowly down”.

Do 3 sets of 10 repetitions with a break of 30-60 seconds between sets.

© 2025 Abafita BJ et al. *JAMA Network Open*.

Exercise 6: Hamstring strengthening
Level 3: Hip Extension with knee straight
Starting position:

Place one end of an elastic band securely around the ankle of your arthritis leg. Place the other end of the elastic around your opposite foot so you are standing on it. Stand and lean forward on your forearms over a table.

Exercise:

Lift your arthritis leg backwards behind you, keeping your knee straight. Lead with your heel, so you are extending at your hip joint. Keep the leg straight.

Hold for 5 seconds then slowly, return to the starting position.

"Move behind, hold, 2, 3, 4, 5, slowly return".

Keep both legs straight.

Keep your hips facing forwards.

Don't twist your hips. Be careful not, to over arch your back. If you feel discomfort in your lower back don't move the leg back as far.

Do 3 sets of 10 repetitions with a break of 30-60 seconds between sets.

© 2025 Abafita BJ et al. *JAMA Network Open*.

Exercise 6: Hamstring strengthening
Level 4: Hip extension knee bent
Starting position:

Stand and lean forward on forearms over a table or bench.

Exercise:

Bend your arthritis knee to around 90 degrees.

Push your foot backwards behind you so you are extending at your hip joint, while keeping your knee in the bent position.

Hold for 5 seconds then slowly return to the starting position.

Do 3 sets of 10 repetitions with a break of 30-60 seconds between sets.

© 2025 Abafita BJ et al. *JAMA Network Open*.

Exercise 7: Calf strengthening
Level 1: Double calf raises
Starting position:

Stand holding onto back of the chair.

Exercise:

Slowly rise up onto your toes.

Hold for 5 seconds.

Slowly lower.

"Slowly up, hold, 2, 3, 4, 5, slowly down".

Do 3 sets of 10 repetitions with a break of 30-60 seconds between sets.

© 2025 Abafita BJ et al. *JAMA Network Open*.

Exercise 7: Calf strengthening
Level 2: Single calf raises
Starting position:

Stand holding onto back of the chair.

Stand on your arthritis leg.

Exercise:

Slowly rise up onto your toes. Hold for 5 seconds.

Slowly lower.

“Slowly up, hold, 2, 3, 4, 5, slowly down”.

Do 3 sets of 10 repetitions with a break of 30-60 seconds between sets.

© 2025 Abafita BJ et al. *JAMA Network Open*.

Exercise 7: Calf strengthening
Level 3: Double calf raises over edge of step
<u>Starting position:</u>

Stand on a step with your heels over the edge.

Hold onto the back of a chair for support.

<u>Exercise:</u>

Slowly rise up onto your toes. Hold for 5 seconds.

Slowly lower down, so your heels are below the step.

“Slowly up, hold, 2, 3, 4, 5, slowly lower all the way down”..

Do 3 sets of 10 repetitions with a break of 30-60 seconds between sets.

© 2025 Abafita BJ et al. *JAMA Network Open*.

Exercise 7: Calf strengthening
Level 4: Single calf raises over edge of step
<u>Starting position:</u>

Stand on your arthritis leg on a step with your heel over the edge.

Hold onto the back of a chair for support.

<u>Exercise:</u>

Slowly rise up onto your toes. Hold for 5 seconds.

Slowly lower down, so your heel is below the step.

“Slowly up, hold, 2, 3, 4, 5, slowly lower all the way down”.

Do 3 sets of 10 repetitions with a break of 30-60 seconds between sets.

© 2025 Abafita BJ et al. *JAMA Network Open*.

Exercise 8: Seated Knee Extension
Starting position:

Sit in a firm chair (one that is higher if possible).

Exercise:

Slowly lift your foot up and straighten the knee until it is fully straight.

Keep the back of your thigh on the chair. Hold for 5 seconds and lower slowly.

"Slowly up, hold, 2, 3, 4, 5, slowly down".

Progression:

Tie your elastic band into a loop.

Place the looped elastic band around the back leg of a chair.

Sit on the chair and put your leg into the loop with the band around the front of your foot.
Change colour of elastic band – red through to black.

Do 3 sets of 10 repetitions with a break of 30-60 seconds between sets.

© 2025 Abafita BJ et al. *JAMA Network Open*.
