# Supplementary material for: Yoga or Strengthening Exercise for Knee Osteoarthritis: A Randomized Clinical Trial
Source: JAMA Netw Open. 2025 Apr 8;8(4):e253698. doi: 10.1001/jamanetworkopen.2025.3698 (PMC11979726; doi:10.1001/jamanetworkopen.2025.3698)
Supplement: Supplement 3. — Data Sharing Statement [file jamanetwopen-e253698-s003.pdf]

Data Sharing Statement

Abafita. Yoga or Strengthening Exercise for Knee Osteoarthritis. *JAMA Netw Open*. Published April 08, 2025. doi:10.1001/jamanetworkopen.2025.3698

Data
Additional Information: Australian New Zealand Clinical Trials Registry (ANZCTR)
https://www.anzctr.org.au/Trial/Registration/TrialReview.aspx?id=380369
ACTRN12621000066886
Data available: Yes
Data types: Deidentified participant data
How to access data: Benny.EathakkattuAntony@utas.edu.au
When available: beginning date: 08-11-2025, end date: 08-11-2027

Supporting Documents
Document types: Statistical/analytic code
How to access documents: Benny.EathakkattuAntony@utas.edu.au
When available: beginning date: 08-11-2025, end date: 08-11-2027

Additional Information
Who can access the data: Researchers whose proposed use of the data has been approved: Data will be shared only with qualified researchers who submit a proposal detailing their intended use of the data, subject to approval by a review committee or data access board.
Types of analyses: For analysis of specific endpoints or outcomes related to the study.
Mechanisms of data availability: After approval of a proposal: Interested parties must submit a research proposal outlining the intended use of the data, which will need to be approved by a review committee or data access board.
